# Evolution of Intra-specific Regulatory Networks in a Multipartite Bacterial Genome

**DOI:** 10.1371/journal.pcbi.1004478

**Published:** 2015-09-04

**Authors:** Marco Galardini, Matteo Brilli, Giulia Spini, Matteo Rossi, Bianca Roncaglia, Alessia Bani, Manuela Chiancianesi, Marco Moretto, Kristof Engelen, Giovanni Bacci, Francesco Pini, Emanuele G. Biondi, Marco Bazzicalupo, Alessio Mengoni

**Affiliations:** 1 Department of Biology, University of Florence, Florence, Italy; 2 Department of Genomics and Biology of Fruit Crops, Research and Innovation Centre, Fondazione Edmund Mach (FEM), San Michele all’Adige, Italy; 3 Dipartimento di Biotecnologie Agrarie, Sezione di Microbiologia, University of Florence, Florence, Italy; 4 Department of Computational Biology, Research and Innovation Centre, Fondazione Edmund Mach (FEM), San Michele all’Adige, Italy; 5 Consiglio per la Ricerca e la Sperimentazione in Agricoltura, Centro di Ricerca per lo Studio delle Relazioni tra Pianta e Suolo (CRA-RPS), Rome, Italy; 6 Interdisciplinary Research Institute USR3078, CNRS-Universit Lille Nord de France, Villeneuve d’Ascq, France; Johns Hopkins University, UNITED STATES

## Abstract

Reconstruction of the regulatory network is an important step in understanding how organisms control the expression of gene products and therefore phenotypes. Recent studies have pointed out the importance of regulatory network plasticity in bacterial adaptation and evolution. The evolution of such networks within and outside the species boundary is however still obscure. *Sinorhizobium meliloti* is an ideal species for such study, having three large replicons, many genomes available and a significant knowledge of its transcription factors (TF). Each replicon has a specific functional and evolutionary mark; which might also emerge from the analysis of their regulatory signatures. Here we have studied the plasticity of the regulatory network within and outside the *S. meliloti* species, looking for the presence of 41 TFs binding motifs in 51 strains and 5 related rhizobial species. We have detected a preference of several TFs for one of the three replicons, and the function of regulated genes was found to be in accordance with the overall replicon functional signature: house-keeping functions for the chromosome, metabolism for the chromid, symbiosis for the megaplasmid. This therefore suggests a replicon-specific wiring of the regulatory network in the *S. meliloti* species. At the same time a significant part of the predicted regulatory network is shared between the chromosome and the chromid, thus adding an additional layer by which the chromid integrates itself in the core genome. Furthermore, the regulatory network distance was found to be correlated with both promoter regions and accessory genome evolution inside the species, indicating that both pangenome compartments are involved in the regulatory network evolution. We also observed that genes which are not included in the species regulatory network are more likely to belong to the accessory genome, indicating that regulatory interactions should also be considered to predict gene conservation in bacterial pangenomes.

## Author Summary

The influence of transcriptional regulatory networks on the evolution of bacterial pangenomes has not yet been elucidated, even though the role of transcriptional regulation is widely recognized. Using the model symbiont *Sinorhizobium meliloti* we have predicted the regulatory targets of 41 transcription factors in 51 strains and 5 other rhizobial species, showing a correlation between regulon diversity and pangenome evolution, through upstream sequence diversity and accessory genome composition. We have also shown that genes not wired to the regulatory network are more likely to belong to the accessory genome, thus suggesting that inclusion in the regulatory circuits may be an indicator of gene conservation. We have also highlighted a series of transcription factors that preferentially regulate genes belonging to one of the three replicons of this species, indicating the presence of replicon-specific regulatory modules, with peculiar functional signatures. At the same time the chromid shares a significant part of the regulatory network with the chromosome, indicating an additional way by which this replicon integrates itself in the pangenome.

## Introduction

Regulation of gene expression is recognized as a key component in the cellular response to the environment. This is especially true in the microbial world, for two reasons: bacterial cells are often under severe energy constraints, the most important being protein translation [1] and they usually face a vast range of environmental and physiological conditions; being able to efficiently and readily react to ever changing conditions can most certainly give a selective advantage over competitors and give rise to specific regulatory networks.

Transcription is mainly regulated by proteins, called transcription factors (TF), which usually contain a protein domain capable of binding to specific DNA sequences, called TF binding sites (TFBS). Depending on the position of the TFBS with respect to the transcriptional start site of the regulated gene, the TF can act either as a transcriptional activator or a repressor, mostly because of its interaction with the RNA polymerase and sigma factors [2, 3]. The binding of the TF to its cognate TFBS is based on non-covalent interactions whose strength is indicated by the so-called affinity constant. Since TFBS can have variations around a preferred sequence, the affinity of a TF for its TFBSs covers a continuous range of values; however, since the TF binding strength appears to follow a sigmoid behaviour, it is possible to distinguish between ‘weak’ and ‘strong’ TFBSs [4].

As opposed to eukaryotic species, prokaryotic TFBSs are usually distinguishable from the ‘background DNA’, and they tend to have a simpler structure and a close proximity to the transcription start site [5]. The application of information theory concepts to TFBS identification and analysis, revealed that specificity of the TF for a certain TFBS depends on the length, variability and composition of the TFBS itself with respect to the overall genomic background (i.e. the sequence composition). Intuitively, the minimum information content able to provide specific recognition of the TFBS by the TF mostly depends on the genome size and its composition; increasing the size of the genome clearly increases the number of putatively non-functional TFBSs, and when the TFBS bases composition is close to the background DNA composition it may be impossible to discern a true functional TFBS from the surrounding DNA. Transcription factors recognizing TFBS characterized by low information content usually control the transcription of many genes across the genome; alternative sigma factors usually belong to this class, and their TFBSs also show larger variability between species [5]. Gene targets of these TFs are harder to reliably predict, for the presence of many non-functional sites along the genome. The high gene density of bacterial genomes and its organization in operons results in specific expression or repression of whole functional pathways in response to stimuli. Furthermore, the presence of several TFBSs in the upstream region of a gene can result in a complex transcriptional response that recall the behaviour of logic gates [6].

Prediction of TFBSs in a genome usually relies on the availability of a position specific scoring matrix (PSSM) storing the frequency of each nucleotide at each position of a TFBS. PSSM modelling the variability of a TFBS can be built by identifying enriched DNA patterns in promoter regions of genes that are known to be under the control of the TF under analysis, better if guided by other assays, like the binding of the TF to synthetic nucleotides. Several algorithms have been developed to use such PSSM to search for TFBSs in nucleotide sequences, such as the MEME suite [7], RSAT [8–10] and the Bio.motif package [11]. A recent alternative method relies on the construction of a hidden markov model (HMM) from an alignment of nucleotide sequences, which can then be used to scan a query nucleotide sequence [12–14]. Since all these methods and their implementations have different weaknesses, it has been advised to use their combination to run predictions [15].

Regulatory networks evolve rapidly, making the comparisons between distant organisms difficult [16–19]. At broad phylogenetic distances, it has been shown that the conservation of a TF is lower than its targets [16]. Additionally, species with similar lifestyles tend to show conservation of regulatory network motifs, despite significant variability in the gene composition of the network, suggesting an evolutionary pressure towards the emergence of certain regulatory logics [16].

The fluidity of most transcriptional regulatory connections is well known and documented, not only at large phylogenetic distances, but also at the level of intra-species comparisons too [20–23]. Experiments have shown that Bacteria have high tolerance towards changes in the regulatory circuitry, making them potentially able to exploit even radical changes to the regulatory network, without extensive changes in phenotypes [24]. However, this is strongly dependent on which regulatory interaction undergoes changes, since there are also examples where a single change determines an observable difference in phenotype [25, 26]. Bacteria have therefore a mixture of robust and fragile edges in their regulatory networks and evolution can play with them at different extent to explore: i) the function of new genes, by integrating them in the old gene regulatory network, and ii) if genes that are part of the gene regulatory network can be removed without harm to the physiology of the cell. The extent of variability and evolution of the regulatory network inside a species is, however, still poorly understood.

The aim of this study is a comparative genomics analysis of regulatory networks, to understand the impact of regulatory network variability on pangenome evolution. We decided to use the *Sinorhizobium meliloti* species, the nitrogen-fixing symbiont of plants from the genus *Medicago*. *S. meliloti* has been deeply investigated as a model for symbiotic interaction and an extensive knowledge on its TFs is present in the literature [27, 28]. This species presents a marked genomic difference with respect to other well-know bacterial model species, such as *Escherichia coli*, since *S. meliloti* genome comprises three replicons of comparable size: a chromosome, a chromid [29] and a megaplasmid, characterized by functionally and evolutionary distinct signatures [30, 31]. This arrangement raises the question of how TF targets are distributed over the replicons. Recent reports have shown that there are only two genes essential for growth in minimal media and soil encoded in the *S. meliloti* chromid [32], even though the chromid harbours many genes shared by all sequenced strains of *S. meliloti* species. Moreover, *S. meliloti* has several genomes sequenced to date [23, 30, 33–39] and the potential for biotechnological and agricultural applications, which could benefit from this analysis. At the comparative genomics level, different strains show quite a high level of variation. Indeed, the pangenome (the collection of all genes from different strains [40]) of this species has an abundant fraction of genes common to all members of the species (termed core genome, as opposed to the strain-exclusive and/or partially shared fraction, called accessory genome) of around 5000 gene families; approximately 40% of the genome belongs to the accessory fraction [31, 35]. A preliminary analysis revealed that some of the TFs of the core genome also control genes of the accessory genome [23]. This allowed to propose that, when comparing the same regulon in different strains, we can define a *panregulon*, including a set of core (shared) target genes and an accessory (variable) regulon fraction [23]. It should be noticed that while the core regulon is necessarily formed by genes belonging to the core genome, the opposite can also be true (i.e. that a gene belonging to the core genome belongs to the accessory regulon). However, the dynamics of the panregulon in relation to the evolutionary rules controlling the variability of the accessory regulon fraction are still not understood.

We have therefore constructed the regulatory network of the *S. meliloti* species, using the PSSMs of 41 TFs collected from the literature and public databases. We have applied a combination of TFBS prediction methods, combining their output with information about the core and accessory gene families. We have also predicted the presence of the same TFBSs in five other closely related rhizobial species (termed ‘outgroups’: *Rhizobium leguminosarum* bv. *viciae*, *Rhizobium etli*, *Mesorhizobium loti*, *Sinorhizobium fredii* and *Sinorhizobium medicae*). This regulatory network has been used to highlight the different behaviours that are present within and between species. Our predictions and other comparative genomics observations are publicly available (https://github.com/combogenomics/rhizoreg/).

## Results

### General features of the predicted regulatory network of *S. meliloti*

Based on COG annotations, all the 51 *S. meliloti* strains analysed in this study, have been found to encode a similar number of predicted TFs (an average of 522); a similar number has been also found in the five outgroups (an average of 533). This is in accordance with previous reports correlating genome size with the number of TFs [41]. Rhizobia belonging to the *Alphaproteobacteria* class (alpha-rhizobia), which are known to have larger genomes compared to other bacteria from the same class [42], have then one of the largest collection of TFs in the known bacterial kingdom. As the accessory genome accounts for about 40% of the proteome size [31, 35], it is reasonable to expect that a similar proportion of TFs will belong to the accessory genome. Indeed, about 70% of the TFs encoded in the *S. meliloti* pangenome belong to the core genome, while the remaining TFs are present in 1–3 genomes only; this orthologous genes distribution is similar to the one observed for the whole pangenome [43] (S1 Fig). However, most of the 41 TFs analyzed in this study were found to belong to the core genome (37), with the only notable exception represented by RhrA, the activator of the rhizobactin regulon, which is absent in 35% of the strains under study, confirming previous analysis [23, 44, 45]. More interestingly, recent reports have demonstrated how the presence of the rhizobactin operon confers competitive advantage over other *S. meliloti* strains in iron limited environments [32]; we could therefore speculate that a significant fraction of the *S. meliloti* strains have a competitive disadvantage in environments with limitation in iron bioavailability. Surprisingly, an ortholog of FixJ (the component of the global two-component system FixJL, which turns on nitrogen-fixation genes in microaerobiosis during symbiosis) was not predicted in two *S. meliloti* strains (A0643DD and C0438LL); the absence of the gene was further confirmed by PCR. Even though such an important regulator has been found to be absent in these two strains, another gene with similar domains (orthologous group SinMel7252, containing gene SMa1686 from the reference strain Rm1021) was found to belong to the core genome. SMa1686 was shown to be regulated by RirA [46], but to the best of our knowledge no indications of its relationships with microaerophilic growth conditions and symbiosis are present. Consequently, we cannot a priori exclude that the regulatory functions of FixJ may be carried on by homologs (as for instance orthologs of SMa1686) in strains A0643DD and C0438LL. Indeed, previous works have indicated that several target genes of FixJ lack a direct symbiotic function, suggesting the presence of functional redundancy in the genome [47].

Sixteen TFs were absent in at least one of the outgroups. Of these, 6 are encoded by pSymA, the symbiotic megaplasmid, including two copies of NodD, FixJ, RctR, SyrM and RhrA (S1 Fig). Such difference between intraspecific and interspecific TF gene content may anticipate a similar difference at the downstream regulatory network, for the absence of cross-regulatory links.

**Fig 1:**
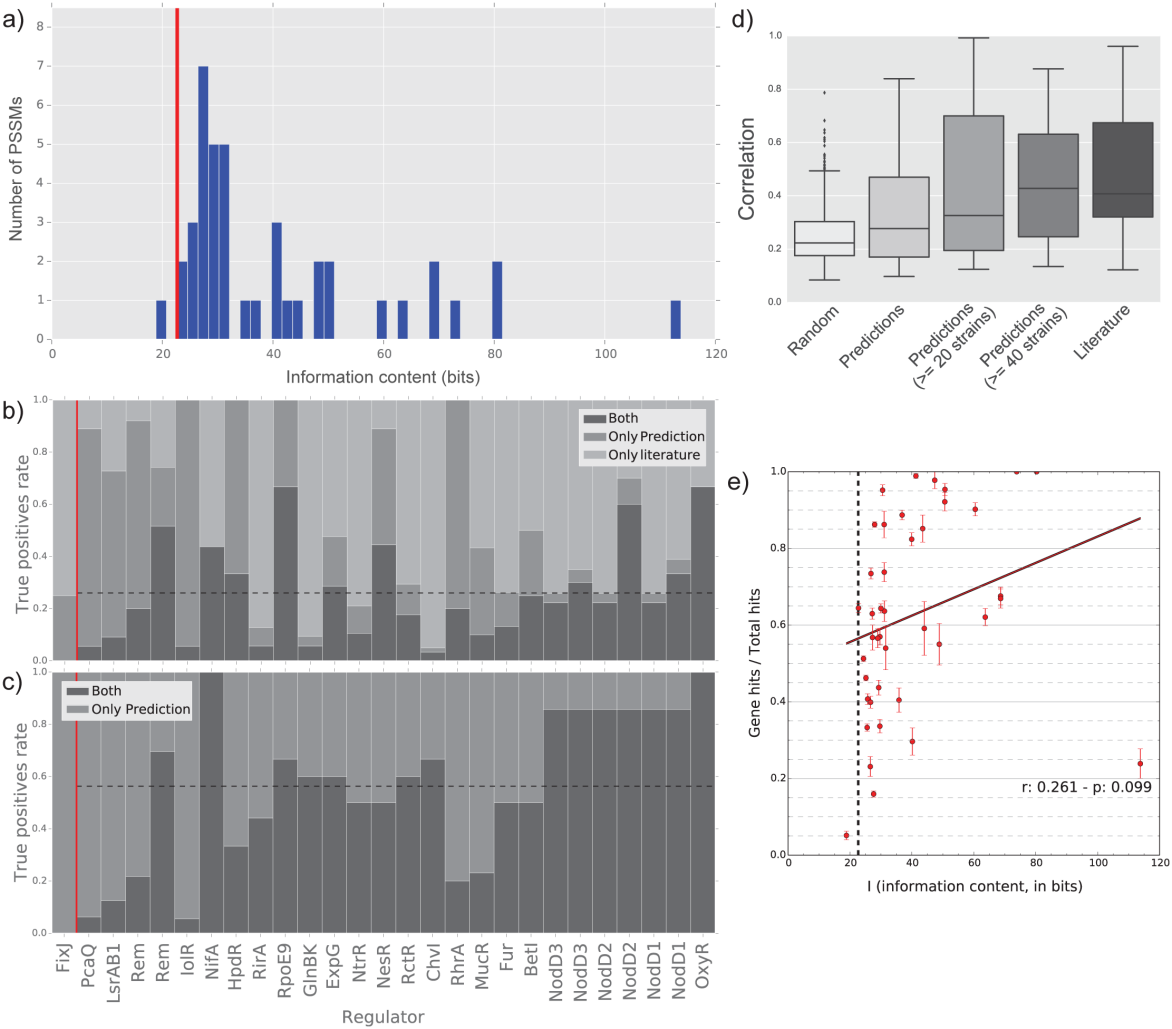
General characteristics of the presented TF predictions and quality control. a) Information content frequencies for the 41 analysed TFs: vertical line indicates the minimum information content, as measured for *S. meliloti* strain Rm1021; b-c) comparison between TFBS predictions and the reported experimental results in strain Rm1021: the dashed horizontal line indicates the mean value for the TFs with information content higher than the minimum value; d) correlations with the COLOMBOS expression compendium for *S. meliloti* Rm1021; e) correlation between the TFs information content and the signal-to-noise ratio, measured as the proportion of prediction in genes upstream regions over the total number of predictions: vertical bars indicate the error level measured in all the strains.

To minimize the number of false positives in our predictions, we selected PSSMs with relatively high information content (over the reference strain minimum information content, see Materials and Methods) A wide range of information gain for PSSMs was observed; of the starting 83 TFBSs retrieved from literature and databases, 41 have been found to have enough information content to reliably predict their TFBSs (Fig 1a, S1 Table). For FixJ, two separate motifs acting together have been described [48], one above and one slightly below the threshold: both motifs have been used.

We have applied a novel TFBS prediction approach to overcome common problems associated with the prediction algorithms and to maximize accuracy and sensitivity [3], including operon predictions to recover most of the downstream regulated genes (see Materials and methods). The predictions accuracy was determined with a comparison with the downstream regulons reported in the literature, when available (Fig 1b and 1c); the average accuracy of the predictions was found to be around 55%, with a tendency to positively correlate with the motif information gain (S2 Fig). This behaviour may be explained by the fact that most regulons have been defined on the basis of gene expression data and therefore contain both direct and indirect targets of the TF; our strategy is then not able to recover the indirect targets which might explain the relatively low accuracy. An example of a known regulatory interaction predicted by our approach is rem (SMc03046), a putative transcriptional regulator involved in the control of motility in *S. meliloti* Rm1021 [49], which was predicted to be under the control of MucR in our analysis (S1 Material).

To provide additional validation to our predictions, we used a compendium of *S. meliloti* gene expression data from the Colombos database [50] (see Materials and Methods). The full compendium contained 424 conditions and was used to calculate average correlation coefficients among the genes of i) the same predicted regulons, ii) the regulons reported in the literature and iii) random groups of genes sampled from the genome (Fig 1d and S2 Material). We have selected the conditions maximising the average correlation for a group of genes using a genetic algorithm (see Materials and Methods). Correlations for our predictions were not significantly different from the experimentally defined regulons; genes belonging to predicted regulons had a slight tendency to be higher than the random regulons, but if this difference was not significant (p = 0.09). We further experimentally confirmed some of the predictions on a subset of predicted promoters of the NodD regulon (S2 Table).

Predicted TFBSs in upstream regions against TFBSs predicted in coding regions were considered as signal to noise ratio (upstream hits on total hits) to measure the predictions quality (Fig 1e); for more than 70% of the analysed TF the observed ratio was above 50%, with a very poor correlation with the motif information content.

Taken together these results show that our predictions are of fairly good quality.

**Fig 2:**
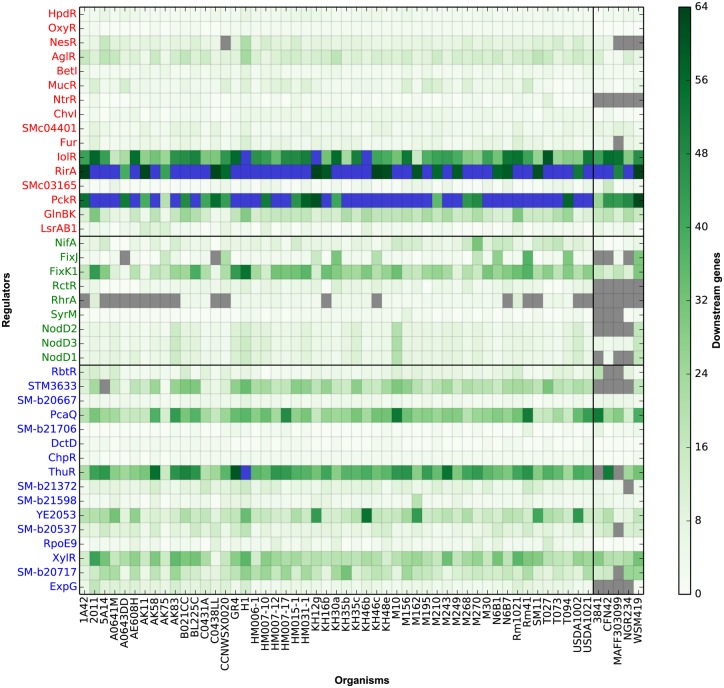
Variability in regulon size. Color intensity indicates the number of downstream regulated genes in each strain; gray squares indicate the TF absence in the genome of that particular strain. Blue squares indicate that there are more than 64 genes predicted to be under the control of the TF. TFs are colored according to the replicon they belong to: red for chromosome, green for the pSymA megaplasmid and blue for the pSymB chromid.

Little variability in the number of genes under the control of each TF was observed among different strains (Fig 2 and Table 1). Each TF was predicted to control the transcription of 12 genes on average, with RirA showing the largest regulon (with an average of 71.6 genes) and SyrM the smallest one (with an average of 1.1 genes). TFs with lower information content TFBSs showed a tendency to control a larger number of genes (S2 Fig), which confirms the influence of the information content on motif recognition. The predicted regulons were found to have comparable sizes in the outgroups; therefore the regulon is conserved in size between different species; this might be the result of the conservation across the species of the TFBS or of more general energy constraints on transcription/translation.

**Table 1:** Regulon downstream genes.

		*S. meliloti*		Outgroups	
Regulator	*Replicon* ^a^	Mean regulon size	*MAD* ^b^	Mean regulon size	*MAD* ^b^
HpdR	Chromosome	3.10	1.0	2.4	1.0
OxyR	Chromosome	1.71	0.0	0.0	0.0
NesR	Chromosome	8.24	1.0	6.0	NA
AglR	Chromosome	11.69	2.0	6.4	4.0
BetI	Chromosome	3.22	0.0	2.2	0.0
MucR	Chromosome	5.20	1.0	2.4	0.0
NtrR	Chromosome	1.57	2.0	NA	NA
ChvI	Chromosome	3.53	1.0	0.6	0.0
SMc04401	Chromosome	4.04	1.0	6.4	9.0
Fur	Chromosome	4.45	2.0	4.5	1.0
IolR	Chromosome	41.80	10.0	45.4	7.0
RirA	Chromosome	71.55	8.0	78.2	4.0
SMc03165	Chromosome	1.69	0.0	4.4	1.0
PckR	Chromosome	69.80	13.0	45.0	3.0
GlnBK	Chromosome	15.35	4.0	16.2	2.0
LsrAB1	Chromosome	2.86	2.0	3.4	3.0
NifA	pSymA	8.02	2.0	2.6	3.0
FixJ	pSymA	5.86	1.0	16.5	NA
FixK1	pSymA	24.27	6.0	16.8	5.0
RctR	pSymA	4.59	2.0	NA	NA
RhrA	pSymA	3.79	1.0	NA	NA
SyrM	pSymA	1.10	0.0	1.0	NA
NodD1	pSymA	6.77	2.0	10.0	NA
NodD2	pSymA	6.41	2.0	17.0	NA
NodD3	pSymA	6.71	2.0	6.4	2.0
RbtR	pSymB	5.67	2.0	9.67	17.0
STM3633	pSymB	19.72	4.0	17.0	NA
SM-b20667	pSymB	3.18	1.0	4.0	0.0
PcaQ	pSymB	27.82	5.0	31.2	10.0
SM-b21706	pSymB	0.80	0.0	3.0	2.0
DctD	pSymB	1.24	1.0	0.0	0.0
ChpR	pSymB	1.88	0.0	0.4	0.0
ThuR	pSymB	37.63	7.0	36.0	28.0
SM-b21372	pSymB	7.33	2.0	3.5	0.0
SM-b21598	pSymB	3.51	1.0	3.4	1.0
YE2053	pSymB	19.47	4.0	13.0	6.0
RpoE9	pSymB	3.35	1.0	0.0	0.0
XylR	pSymB	22.61	5.0	24.2	2.0
SM-b20717	pSymB	15.24	4.0	19.25	11.0
SM-b20537	pSymB	7.77	3.0	12.0	1.0
ExpG	pSymB	6.0	2.0	2.0	NA

Regulatory network general statistics over the strains used in this study.

^*a*^ Position according to the Rm1021 reference strain;

^*b*^ Mean Absolute Deviation;

NA: not defined.

Besides similar regulon sizes, we found that an average 40% of genes belonging to a regulon belong to the accessory genome (Table 2); this implies that although variable, each TF recruits a similar number of genes under its control, at least in the species analysed here. Obviously, the variability of the regulons is related with both the variability in upstream regions of core genes and the presence of genes from the accessory genome (whose presence varies across and between the species) in the regulons.

**Table 2:** Regulon conservation.

Regulator	Replicon	*S. meliloti*	*Outgroups* ^a^
HpdR	Chromosome	0.56	0.95
OxyR	Chromosome	1.00	1.00
NesR	Chromosome	0.57	0.52
AglR	Chromosome	0.57	0.48
BetI	Chromosome	0.33	0.50
MucR	Chromosome	0.89	0.71
NtrR	Chromosome	0.56	NA
ChvI	Chromosome	0.98	0.86
SMc04401	Chromosome	0.56	0.74
Fur	Chromosome	0.49	0.73
IolR	Chromosome	0.59	0.52
RirA	Chromosome	0.58	0.56
SMc03165	Chromosome	0.68	0.65
PckR	Chromosome	0.60	0.56
GlnBK	Chromosome	0.58	0.63
LsrAB1	Chromosome	0.56	0.71
NifA	pSymA	0.57	0.74
FixJ	pSymA	0.56	0.63
FixK1	pSymA	0.56	0.63
RctR	pSymA	0.57	NA
RhrA	pSymA	0.56	NA
SyrM	pSymA	0.57	0.00
NodD1	pSymA	0.56	0.65
NodD2	pSymA	0.56	0.66
NodD3	pSymA	0.57	0.69
RbtR	pSymB	0.57	0.46
STM3633	pSymB	0.57	0.63
SM-b20667	pSymB	0.57	0.53
PcaQ	pSymB	0.57	0.51
SM-b21706	pSymB	0.96	0.75
DctD	pSymB	1.00	1.00
ChpR	pSymB	0.57	1.00
ThuR	pSymB	0.58	0.47
SM-b21372	pSymB	0.57	0.51
SM-b21598	pSymB	0.57	0.70
YE2053	pSymB	0.58	0.50
RpoE9	pSymB	0.99	0.60
XylR	pSymB	0.57	0.54
SM-b20717	pSymB	0.56	0.52
SM-b20537	pSymB	0.56	0.50
ExpG	pSymB	0.56	0.45

Regulatory network conservation in *S. meliloti* and near rhizobial species. For each regulator the number of conserved downstream genes over the average regulon size is reported.

^*a*^ *S. meliloti* strain Rm1021 is also considered.

NA: not defined.

Predictions for TFs with low information content TFBSs showed a very poor accuracy and precision when compared to experimental data found in the literature; an efficient search strategy for such TFBSs using PSSM has still to be developed. However, from an evolutionary point of view, since those TFs are predicted to bind rather aspecifically to many sites along the genome, this would result in even a larger divergence of regulons between strains, as recently reported in comparison among species [51].

### Upstream sequences and accessory genome changes are correlated with regulon diversity

To clarify if the patterns of variability of the regulatory network are related to the phylogenetic distance among strains a comparison between divergence of panregulons and divergence of pangenomes was performed.

Following the pangenome analysis, we calculate three sets of distance matrices among the genomes under analysis (see Materials and Methods): the first was obtained from the alignment of core genes (hereinafter the *core distance*), the second from alignments of the upstream regions of the core genes (the *upstream distance*), and the third is instead based on the presence/absence profiles of accessory genes (*gene content distance*). The three distances were then compared with the *regulatory network distance* of the corresponding strains/species, which was calculated with the same metric defined by Babu and collaborators [16]. Intuitively, the divergence in upstream regions should be paralleled by divergence in the regulatory network, since the former will at some point determine a loss/gain of TFBSs affecting the structure of the regulatory network. Similarly, a larger difference in gene content should also be mirrored by a higher variability in the regulatory network, since new genes may be recruited in the regulatory network and/or TFs may be lost/gained. On the other hand, we don’t expect to observe a strong correlation between core and regulatory network distances; this is also due to the lower divergence at the coding level between strains, implying that regulon diversity inside a species could be driven by gene content variability and upstream sequences variability.

**Fig 3:**
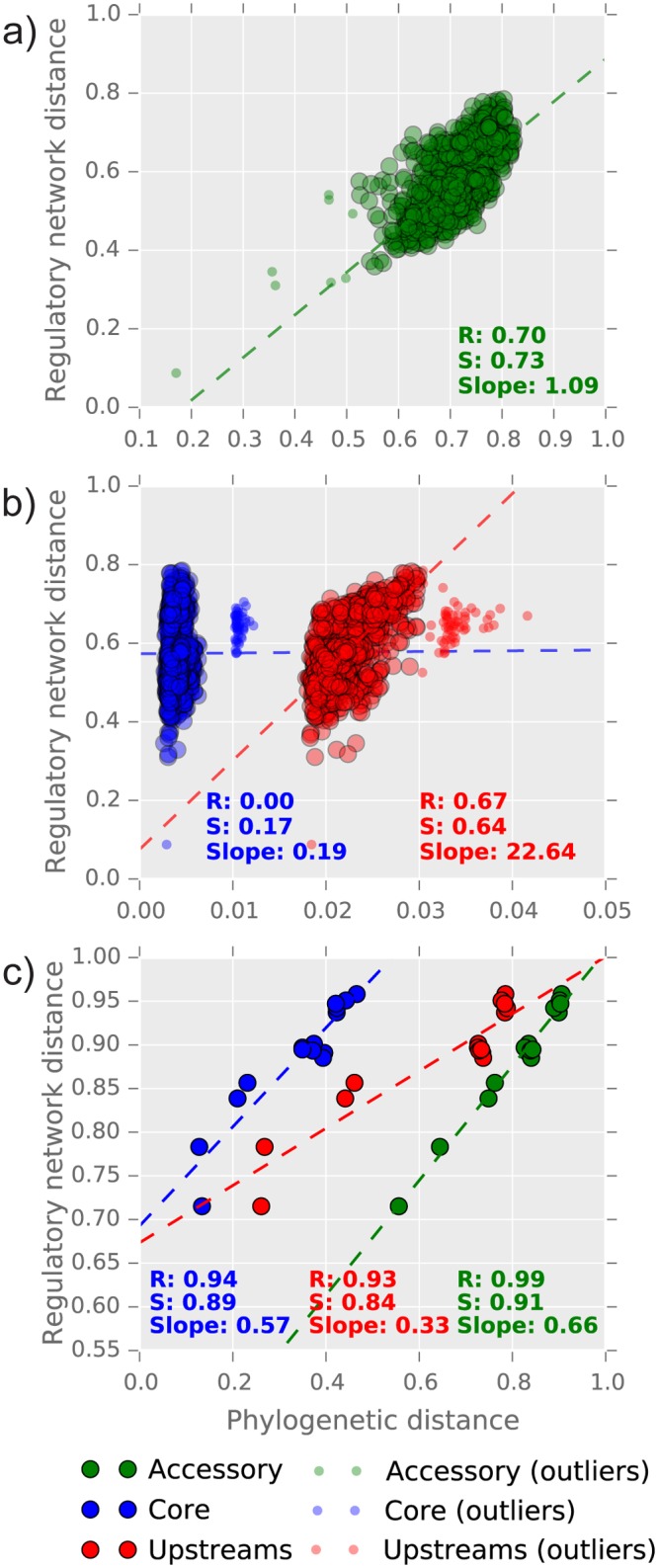
Correlations between pangenome diversity and regulatory network distances. R and S indicate the Pearson’s and Spearman’s correlation coefficients between the regulatory network and each pangenome partition distances (see Materials and Methods for the definition of the distances metrics used here). Outliers have been defined using a Z-score threshold of 3.5 on the mean absolute deviation of the distances. a) correlations within the *S. meliloti* species for the accessory genome; b) correlations within the *S. meliloti* species for coding and upstream regions; and c) correlation between the outgroups.

These hypotheses on patterns of correlations between pangenome differences and regulatory divergence were confirmed at the species level (Fig 3a and 3b). The comparison between *S. meliloti* strains showed that the regulatory network distance is correlated with both the upstream distance and with gene content distance. The core distance showed no significant correlation with the regulatory network distance (Fig 3b). When considering the outgroup species, all three distances were found to be similarly correlated with the regulatory network distance (Fig 3c). Since the divergence in coding sequences cannot directly influence transcriptional regulation (with the exception of non-synonymous mutations in the DNA binding domain of a TF), we propose that the most likely explanation of the observed correlations is the overall genome divergence between species, which is ultimately reflected by a higher divergence at the regulatory network level. This is also confirmed by the high correlation coefficients among the three distances. We then concluded that the patterns of regulatory network variation are paralleled, at the species level, by changes in promoter sequences and by the variation in the accessory genome composition, at least in *S. meliloti*. These two fractions of the pangenome could then be used as *bona fide* predictors of the extent of rewiring in regulatory networks. However, from these data we cannot confirm a direct causative explanation for the observed regulatory network variation, as this analysis has been focused on the whole pangenome. The striking difference between the slow rate of coding sequence evolution versus the much larger difference in the regulatory networks is however worth noting.

### Evolutionary dynamics of regulatory networks

**Fig 4:**
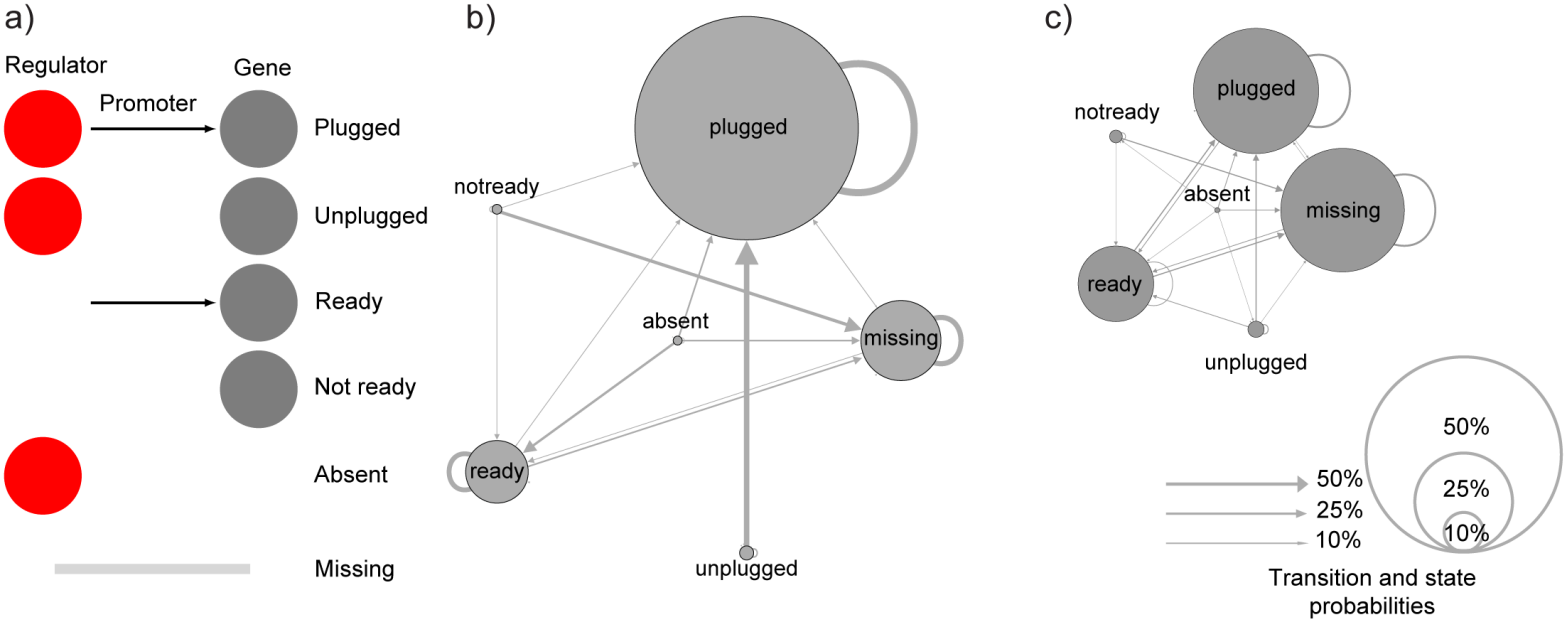
Regulatory network dynamics. a) Graphical representation of the six states in which each regulatory link (a gene found with a TFBS in at least one genome) can be found in the *S. meliloti* species and between the outgroup species; b) states probabilities and states transitions probabilities inside the *S. meliloti* species: nodes and edges sizes are proportional to the probability in the model. For each state, the sum of transition probabilities is one; transition probabilities below 0.1 are not shown; c) states probabilities and states transitions probabilities between the outgroup species.

Regulatory network evolutionary dynamics showed interesting differences within and between species. Each observed regulatory interaction in the two datasets (*S. meliloti* and the outgroups) and its state across all strains was used to build a hidden markov model to infer the preferred state transitions in our predictions (see Materials and methods), that corresponds to the ways the gene regulatory network can grow and shrink. The possible states of a target gene depend on the presence of the TF, the target gene itself and the upstream TFBS. Therefore, each target gene can be found in one of six different states (Fig 4a). The “plugged” state being the only functional one, which corresponds to a target gene with a TFBS in its promoter region when the TF is present in the genome. The other five are non-functional states but may represent transitory states during the evolution of gene regulatory networks. Each of these states lack: i) the TFBS (“unplugged”), ii) the TF (“ready”), iii) both the TF and the TFBS (“not ready”), iv) the regulated gene (“absent”) or v) both the TF and the gene itself (“missing”). This HMM can be used to estimate the probability for state transitions, that is the probability of observing a change from one state to another between two strains. This results in a model that is able to provide a general description of the evolution of regulatory networks within and between bacterial species. Since the models is based on observed states in the available strains, we consider it as a “snapshot” of the regulatory network evolution, and not an equilibrium model.

According to the model, the most represented state in the *S. meliloti* regulatory network is the “plugged” one, indicating conservation of regulatory interactions at the species level (Fig 4b and S3 Table). More interestingly, the model predicts that the “unplugged” genes are mostly seen recruited by the regulatory network and that the regulatory link is then maintained with high probability. Very little probability was given to the “plugged” to “missing” and “plugged” to “absent” transitions, indicating that genes belonging to the gene regulatory network are rarely removed from the genome. On the other hand, genes with no TFBS and its cognate TF are more frequently found to undergo loss (“not ready” to “missing”), suggesting that regulatory interactions are important for gene conservation at the species level. When considering a wider phylogenetic level (the outgroups), the broader variability in TF gene targets resulted in the “plugged” and “missing” state as equally probable, indicating that regulons might evolve by adding and removing new elements to a conserved kernel of gene targets (Fig 4c and S3 Table). This is also reflected in a smaller probability that a target gene i) remains in the “plugged” state when compared to the *S. meliloti* species level, and ii) that it acquires a TFBS. On the other hand, the same probability as within the *S. meliloti* species was observed for the transition “not ready” to “missing”, which seems to confirm the importance of regulatory features in explaining the accessory genome fraction evolution. Consequently, a different evolutionary dynamics of regulatory circuitry changes seems to be present in relation to the taxonomic ranks; at the species level, robust networks are formed and they tend to include new genes from the species pangenome, which then may be conserved. On the contrary, when comparing wider taxonomic ranges, regulatory networks are less conserved and genes are apparently included in each species’ genome directly with their regulatory features (in a sort of plug-and-play model).

### Replicon-specific regulation and cross-regulation

**Fig 5:**
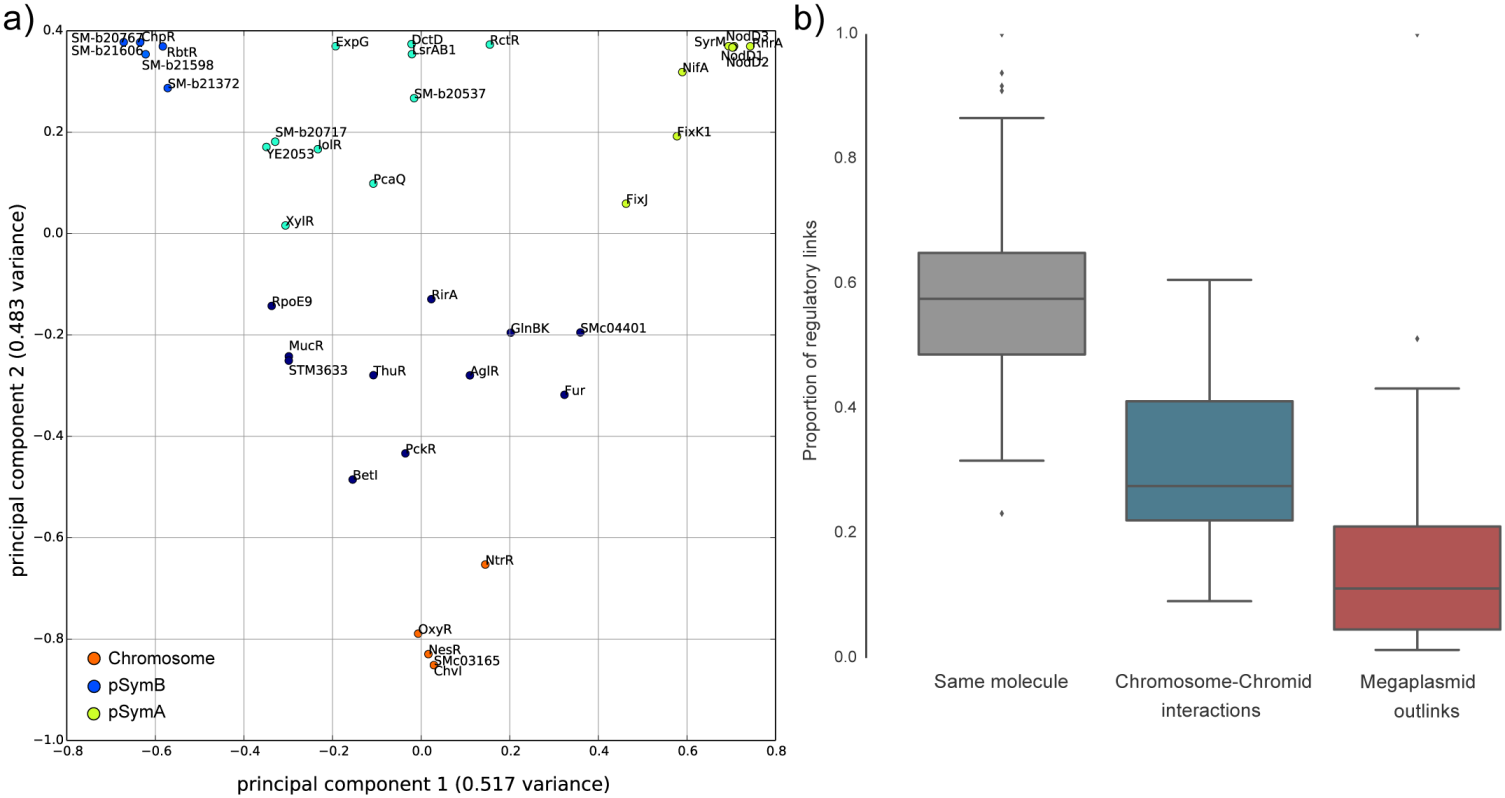
TFs preferentially associated with a replicon. a) K-means clustering of the normalized proportion of genes regulated in each of the three main replicons of *S. meliloti*, visualized in a two-dimensional PCA. The dark blue and cyan clusters contain TFs with no clear replicon preference; b) Variability in the number of regulatory links in the same replicon and between replicons. All differences are significant (t-test p-value < 0.05).

Transcription factors with replicon preference were found to have functional signatures in accordance with the functions encoded in the three main replicons of *S. meliloti*. This aspect has been evaluated by mapping each draft genome on the *S. meliloti* replicons (see Materials and methods) and considering the presence of each gene in the replicons for each of the 51 strains analysed here. Using a clustering approach on normalized gene hits on each replicon we have found that 19 TFs preferentially regulate genes belonging to one of the three replicons: five to the chromosome (NtrR, OxyR, NesR, ChvI and SMc03165), six to the pSymB chromid (SM-b21706, SM-b20667, ChpR, RbtR, SM-b21598 and SM-b21372) and eight to the symbiotic megaplasmid pSymA (SyrM, NodD3, RhrA, NodD1, NodD2, FixJ, FixK1 and NifA) (Fig 5a); these TFs are also encoded by the same replicon.

The six TFs encoded by the pSymB chromid (whose regulon is also preferentially located on pSymB) appear to mostly regulate the transport and metabolism of various carbon and nitrogen sources, including ribitol (RbtR), tagatose, sorbitol and mannitol (SM-b21372), ribose (SM-b21598), lactose (SM-b21706) and tartrate, succinate, butyrate and pyruvate (SM-b20667). The eight TFs present in the symbiotic megaplasmid pSymA (with regulons preferentially located on pSymA) were found to be involved in the regulation of key symbiotic processes, including nitrogenase synthesis and functioning through micro-aerophilia (FixJ, FixK1 and NifA), nod-factors biosynthesis (SyrM, NodD1, NodD2 and NodD3), and iron scavenging (RhrA).

A functional enrichment analysis using COG annotations (S3 Fig) on genes belonging to the regulons of the replicon-biased TFs confirmed this general observation: no functional category was enriched in the chromosome. The G category (*carbohydrate metabolism and transport*) was enriched in genes regulated by pSymB encoded TFs, in agreement with the role of chromid pSymB in providing metabolic versatility to *S. meliloti*. The C (*energy production and conversion*), U (*intracellular trafficing and secretion*) and T (*Signal Transduction*) categories were enriched in genes under the control of pSymA-harboured TFs, which show some relationship with the establishment on the plant symbiosis. This analysis allowed us to depict a scenario where a significant part of the regulatory network is replicon-specific, with a tendency to maintain the functional signature of the host replicon, thus confirming earlier reports on the evolutionary independence of chromids and megaplasmids in *S. meliloti* [29, 31, 32].

Interestingly, a fraction of TFs have target genes which span over different replicons, and show a preference for cross regulation between the chromosome and the chromid (Fig 5b). The presence of cross-replicon regulons, may indeed allow a stabilization of genomic structure, genetically and metabolically connecting chromosome encoded functions with those present in the other two *S. meliloti* replicons. In the evolutionary model of the chromid [29, 31, 32], its stabilization within the host genome is related to the acquisition of essential (core) genes in a previously introgressed megaplasmid which gained niche-specific genes. Here, we found that for TFs encoded on the chromosome (as AglR, GlnBK, IolR, BetI, LsrAB, MucR, PckR, RirA, NesR) a variable number of target genes are present on pSymB (S1 Material). The preference for cross-regulation between the chromosome and the chromid, as opposed to the megaplasmid uncovers an additional mechanism by which a chromid integrates itself in bacterial pangenomes.

## Discussion

Regulatory networks are key components of cell’s response to environmental and physiological changes. In the past years, several works have highlighted a high transcriptomic variability in strains or individuals from the same species [52, 53], in addition to genomic variation. Consequently, regulatory network variation might have profound impact on local adaptation and fitness of organisms. Recent studies have confirmed that bacterial regulatory networks are able to tolerate the addition of new genes [24], which in turn can serve as raw material for selection to operate. Using our original combined search strategy, we indeed found variability in regulon composition within the *S. meliloti* species, which in fact accounted on average on 40% of the regulon of each strain. On the other hand the regulon size was found to be conserved even outside the species boundary. This could suggest that even though the genes under the control of a TF vary between strains, there is a general constraint on the size of the transcriptional response. Whether this is due to energy constraints or being simply an effect due to the genome base composition is yet to be clarified.

We found that the regulatory network distance (as defined in [16]) correlates with the upstream distance and also with the gene content distance. This correlations may suggest that regulatory network composition is influenced by both promoter variability and accessory genome variability. Indeed, we may speculate that the sequence divergence in upstream regions can result in the appearance or disappearance of TFBSs, thus changing the regulatory network content. Moreover, gene content dynamics may also have a strong impact on the regulatory network, with the introduction of new gene cassettes containing TFBS recognized by resident TFs. We can consequently hypothesize that the evolution of bacterial regulatory networks, as that of the pangenome, may be influenced by mechanisms of gene acquisitions, such as lateral gene transfer, and it’s not only linked to mutations in upstream regions.

The observed changes in the regulatory network also show interesting features with respect to pangenome composition. Indeed, even if a significant difference in the state transitions of regulatory links inside and outside the species boundary has been shown, for genes that lack both a TFBS and their cognate TF, we have observed a similar tendency to disappear from the pangenome. This observation may suggest that the dynamics governing pangenome evolution within a species could depend in part on a ‘gene fitness’ related to being wired into the regulatory network. We can then propose that regulatory networks have an important role in shaping the bacterial gene content and can contribute to gene fitness, which in turn may be linked to environmental adaptation.

Moreover, the preference of nineteen TFs for target genes on one of the three replicons of *S. meliloti* indicates that in multipartite bacterial genomes, similarly to replicon-dependent patterns of evolution in gene and functions content [31], a replicon-specific transcriptional regulation is to be expected. At the same time, a significant number of cross-links between the chromosome and the chromid suggest for the first time an additional mechanism by which new replicons can be integrated into a bacterial pangenome.

## Materials and Methods

### Genome sequences

The 51 genomic sequences belonging to *Sinorhizobium meliloti* and the five genomic sequences from closely related symbiotic species are listed in S4 Table.

### Orthology

The orthology relationships inside the 51 *S. meliloti* strains has been computed using the Blast-BBH algorithm implemented in the DuctApe suite (version 0.13.0) [54], using default parameters. The same analysis has been conducted on the five closely related species with the addition of the Rm1021 reference strain, using the BLOSUM62 scoring matrix to account for their greater sequence diversity.

### Regulators estimation

The number of regulators present in each genome has been estimated using COG annotations. The similarity of each protein against the COG database has been measured with a rpsblast scan [55], using an E-value threshold of 1e-10. Each protein mapped to the COG category K (Transcription) has been considered as a putative regulator.

### Confirmation of the absence of the *fixJ* gene

To confirm the absence of the *fixJ* gene in strains A0643DD and C0438LL, PCR primers amplifying a large portion (from nucleotide position 32 nt to 595 out of 615 nt total) of the coding sequence of *fixJ* gene have been designed on the basis based on the ortholog sequence in strain BL225C (SinmeB_6173) with Primer3Plus (fw: 5′-ACGAAGAGCCGGTCAGGAAGTCGCTGGCATTCATGCTG-3′; rv 5-CGGCGAGAGCCATGCGAACGAGATGGGGGAGGCTC-3) [56]. PCR has been performed with the Maxima Hot Start Green Master Mix (Thermo Fisher) in 20 microL total volume by using 10 ng of DNA, purified from liquid culture with FAST DNA Kit (QBiogene) and 10 pmols of each primer. Cycling conditions were as follows: 5′ 94°C, followed by 30” 94°C, 30” 55°C, 1′ 72°C repeated for 35 cycles. PCR products were resolved after agarose gel electrophoresis (1.5 w/v) in TAE buffer with ethidium bromide (10 microg/ml) as staining agent.

### Regulatory motifs collection

The 83 regulators whose PSSM has been extracted from the various sources are listed in S1 Table. For those PSSMs retrieved from the literature, we collected the upstream regions of the regulated genes and (when available), the consensus binding sites from bibliographical records; the upstream regions have then been analysed with the *meme* program [7](version 4.9.0), using the model that retrieved the PSMM with higher similarity to literature. Twenty-two motif files have been generated using the information retrieved from the RhizoRegNet database [27]. Fifteen motif files have been generated using the information retrieved from the RegTransBase database [57]. For the 5 regulators having more than one predicted motif, for instance those having a variable length (FixJ, RpoD, RpoE2, RpoH1 and RpoH2), one motif file for each motif length has been generated. All the retrieved PSSMs have been converted to HMM models using the *hmmbuild* program from the HMMer suite [12–14](version 3.1b1), using the alignments present in the MEME motif file. It has been previously shown that in bacterial genomes TFBS can be reliably distinguished from background DNA only if their information content is higher than the minimum information content for the target genome, which depends on the genome size and composition [5] (this simplification of course ignores other factors such accessibility or proximity of the RNA polymerase). The information gain of the TFBS with respect to the genome is calculated using the Kullback-Leibler divergence between the corresponding nucleotide frequencies [58], and it has been shown to correlate with the motif length and base composition of the motif with respect to the surrounding genome sequence. TF motifs with sufficient information content also tend to show less variability in their regulon composition between species [51]; by focusing our analysis on such TFs we ensured a more precise analysis. The information content of each motif has been calculated as suggested by Wunderlich et al [5], using the Rm1021 reference genome for the calculation of the minimum information content; given the dependence of this variable on genome size and the fact that all the *S. meliloti* strains have similar genome size, there has been no need to calculate a strain specific threshold. PSSMs whose information content was found to be lower the minimum information content have been discarded with exception of FixJ, which has two distinct PSSM, one of which is above the threshold. In the presence of more than one source for a regulator (literature, RhizoRegNet or RegTransBase), the PSSM having the highest information content has been considered in the final analysis.

### Search of regulatory motifs occurrences

For each genome, background k-mers frequencies have been calculated using the *fasta-get-markov* program from the MEME suite (version 4.9.0) [7], using 3 as the maximum value for k. Each regulatory motif has been searched inside each genomic sequence using four scanning algorithms. The *mast* program from the MEME suite (version 4.9.0) [7] has been used with an E-value threshold of 100 and the use of a genome-specific background file. The *matrix-scan* program from the RSAT suite [8–10] has been used with a P-value threshold of 0.001, the background file and a pseudocount of 0.01, as suggested by Nishida et al. [59]. The *Bio.motifs* package from the Biopython library (version 1.62b) [11] has been used with a false negative rate threshold of 0.05 and a pseudocount of 0.01, as suggested by Nishida et al. [59]. The *nHMMer* program from the HMMer suite (version 3.1b1) [12–14] has been used with an E-value threshold of 100 and with all the heuristic filters turned off. Each regulatory motif hit has been parsed, separating the hits being present in the upstream region of a gene from the others. The upstream region has been defined as the intergenic region (not overlapping any coding sequence) in front of the first codon with a maximum size of 600 bp. In the case of a palindrome motif, the motif orientation has been ignored.

The distributions of the raw scores has been tested using a normality test, as implemented in the SciPy library (version 0.13.3) [60][61]. The score threshold has been determined through the calculation of the raw scores quartiles (Q1 and Q3) and defining the score threshold (*τ*_*S*_ in Eq 1) in order to consider only the upper outliers [62].

$$\begin{array}{c} \tau_{S}=Q3+\left(1.5\left(Q3-Q1\right)\right). \end{array}$$ (1)

For the Biopython method the bit score has been used, while for the RSAT, HMMer and MEME methods the negative base 10 logarithm of the E-value has been considered. The regulatory motifs predicted by at least three methods have been considered for further analysis.

### Validation of the predictions

The compendium of gene expression data for *S. meliloti* str. Rm1021 from the Colombos database [50] was used to calculate correlation coefficients among genes in the regulons reported in the literature, our predictions and random sets of genes. Random regulons were produced by random sampling groups of genes of size 5, 10 and 15, for which 500 sets were produced. Correlation was quantified by the squared uncentered correlation coefficient, which was calculated using Matlab, as the square of 1 − *cos distance*. Values plotted in Fig 1d are averages over the entire set of genes under analysis. We have implemented a strategy allowing to select the conditions maximizing the average squared correlation within a group of genes, since many of the conditions of the compendium are likely not related to our predictions. Selection of the conditions was performed using the genetic algorithm implemented in the GA Matlab function, with default tolerances (TolCon = 10^−6^, TolFun = 10^−6^). We let the algorithm select the conditions minimizing $\frac{1}{R^{2}}$ where *R* is the uncentered correlation averaged over all pairwise comparisons made within the group of genes under analysis. Since we noticed that correlations are strongly and inversely correlated with the number N of included conditions, especially when *N* ≤ 20, we discarded all cases where the number of conditions was less than 20 (final *N* = 950). All conditions containing missing data in at least one of the genes under analysis were discarded before starting the procedure. For some of the known and predicted regulons, correlations were not calculated as the available number of conditions after removing missing data was less than 30 before the optimization.

### Experimental confirmation of promoters

Upstream sequences from selected putative target genes of NodD regulon were analysed (see S2 Table). Sequences (approximately 400 nt upstream the translation start site of the gene) were amplified from crude lisates of *S. meliloti* strains with AccuPrime *Pfx* DNA Polymerase (Thermo Fisher) and cloned into pTO2 vector (which carries GFPuv as reporter gene [63]) by using *SalI* and *KnpI* restriction sites. Recombinant clones of *E. coli* S17-1 strain were selected by gentamycin resistance and verified by sequencing of inserted fragments. Positive clones were used for transferring recombinant pOT2 vectors to *S. meliloti* Rm1021 by bi-parental conjugation by using previously described protocols [64][65]. *S. meliloti* Rm1021 recombinant strains were then tested for GFP fluorescence after incubation of a 5 ml culture grown at the mid-exponential phase with 1 microM luteolin (Sigma-Aldrich) in liquid TY medium at 30°C for 3h. GFP fluorecence was measured on a Infine200 Pro plate reader (Tecan). Measures were taken in triplicate and normalized to cell growth estimates as absorbance to 600nm.

### Operon prediction

The operons belonging to the 56 genomes of this study have been predicted using the Operon Prediction Software (OFS, version 1.2) [66], using a beta threshold of 0.7 and a probability threshold of 0.5. The number and length of the predicted operons in each strain are listed in S5 Table.

### Replicon mapping

Each contig of the 44 *S. meliloti* draft genomes has been mapped to the seven complete genomes using CONTIGuator (version 2.7.3) [67], using a 15% coverage threshold and considering blast hits over 1000 bp in length. A contig has been considered mapped to a replicon when it has been found mapped to the replicon in at least five complete genomes, or when it has been mapped to the replicon in at least one complete genome and to no replicon in the others. Knowing that very few portions of the *S. meliloti* genome are shuffled between replicons [31], we assessed the quality of this mapping procedure by checking whether the *S. meliloti* orthologs were found to be mapped to more than one replicon; for each orthologous group the genes not mapped to any replicon have been removed, and the relative abundance of the most representative mapped replicon has been computed. A relative abundance of 1 means that the orthologs have all been mapped to the same replicon in all the strains. The vast majority of the orthologous groups was found to map to a single replicon (S4 Fig).

The number of average gene hits has been divided for each replicon (either from a complete genome or a draft genome) and normalized by the number of genes belonging to each replicon in the Rm1021 reference strain. Regulators with preferential regulatory hits in a specific replicon have been highlighted performing a k-means clustering (k = 5, selected using an elbow test [68]) and plotted using the two principal components of the proportion of hits in each replicon, using the scikits-learn package (version 0.14.1) [69]. Only the three main replicons (chromosome, pSymB and pSymA) have been considered. COG categories enrichments have been tested using a Fisher’s exact test, as implemented in the DendroPy package [70].

### Phylogenetic distance

Phylogenetic distance inside the *S. meliloti* pangenome and the pangenome of the five related species has been computed as described in a previous work [31]. The pangenome has been divided in three fractions, allowing the use of three distinct phylogenetic distances. The “core” distance has been calculated through the alignment of all the nucleotide sequences of each core gene, discarding those genes where at least one sequence was 60bp shorter or longer with respect to the other sequences. The “upstream” distance has been calculated through the alignment of the core genes upstream regions, discarding sequences below 5bp in length. The alignments have been calculated using MUSCLE (version 3.8.31) [71] and the bayesian tree has been inferred using MrBayes (version 3.2.0) [72]. The distance matrix for both distance categories has been computed from the phylogenetic tree using the textitBio.Phylo package inside the Biopython library (version 1.62b) [73]. The “accessory” distance has been calculated through the construction of a presence/absence binary matrix for all the accessory genome OGs; the distance between each strain has been then calculated using the Jaccard distance measure, as implemented in the SciPy library (version 0.13.3) [61].

### Regulatory network distance

The distance between each strain inside the *S. meliloti* and the other five related species regulatory network has been computed using the distance in the presence/absence of regulatory interactions as suggested in the work of Babu and collaborators [16]. The distance between strain A and B is computed using Eq 2.

$$\begin{array}{c} D_{AB}=1-\frac{core_{AB}}{total_{AB}}, \end{array}$$ (2)

where *core*_*AB*_ and *total*_*AB*_ represent the number of conserved and total regulatory interactions, respectively.

Pearson and Spearman correlation coefficients between the pangenome and the regulatory network distance have been calculated using the implementations of the SciPy library (version 0.13.3) [61], removing the outliers using a Z-score threshold of 3.5 on the mean absolute deviation of the distances.

### Regulatory network transistions

The state transitions of the regulatory network has been inferred by encoding them in a hidden markov model. Each one of the regulatory links observed in at least one strain has been tested for their state in each organism, following the labelling of Fig 4a. Specifically, each regulatory link in the network of each organism could belong to one of the following categories:

- **Plugged:** regulator, gene and TFBS present
- **Unplugged:** regulator and gene present, TFBS absent
- **Ready:** gene and TFBS present, regulator absent
- **Not ready:** gene present, regulator and TFBS absent
- **Absent:** regulator present, gene and TFBS absent
- **Missing:** regulator, gene and TFBS absent

The hidden markov model has been constructed using the Baum-Welch algorithm [74], as implemented in the GHMM python library. For each observed regulatory link in the regulatory network, the observed transition between each permutation of pairs of strains has been used to train the HMM and then compute the states and transitions probabilities. The transition probability has been defined for each state as the probability of observing the transition between two strains. Since each state has different transition probabilities and their sum is one for each state, we do not observe symmetrical probabilities.

### Results analysis and visualization

Regulatory motifs data has been analysed and visualized using the NumPy [75] and matplotlib [76] libraries inside the iPython environment [77]. Regulatory networks have been built using the networkx library [78] and visualized using Gephi [79].

### Data and methods availability

Genomic sequences, regulatory motif files and search and analysis scripts are available as separate git repositories. The rhizoreg repository (https://github.com/combogenomics/rhizoreg/), contains the input data; the regtools repository (https://github.com/combogenomics/regtools/) contains the main scripts used to conduct the analysis.

## Supporting Information

S1 Material Inter and intra-regulation in the 51 S. meliloti strains. (ZIP)

S2 Material Single regulons correlations with the COLOMBOS expression compendium. (ZIP)

S1 Table Sources and information content of the TF PSSM of this study. (XLS)

S2 Table Experimental validation of NodD targets. (CSV)

S3 Table State transitions probability for the regulatory networks. (CSV)

S4 Table Genomic sequences used in this study. (CSV)

S5 Table Predicted operons statistics. (CSV)

S1 Fig Total TFs encoded in the pangenome. a) TFs frequency (expressed as the number of strains having the TF encoded in their genome) in S. meliloti and the other rhizobial genomes; b) TF presence/absence matrix in the strains analysed in this study: red indicates the TF absence. TFs are colored according to the replicon they belong to: red for chromosome, green for the pSymA megaplasmid and blue for the pSymB chromid.
(TIF)

S2 Fig Correlation between predictions quality and TF information content. Vertical dashed line indicates the minimum information content for S. meliloti strain Rm1021. a) Correlation between predictions true positive rate and information content; b) Correlation between the number of predicted regulated genes and information content.
(TIF)

S3 Fig COG categories enrichment in the replicons. For each replicon, the proportion of regulated downstream genes belonging to each category is compared with the genes belonging to other replicons. Purple categories indicate a statistically significant enrichment.
(TIF)

S4 Fig Replicon mapping quality control. For each orthologous group in the S. meliloti pangenome, the abundance of the most mapped replicons has been computed as a proxy for the consistency of the replicon mappings.
(TIF)
