# Supplementary material for: Evolution of Intra-specific Regulatory Networks in a Multipartite Bacterial Genome
Source: PLoS Comput Biol. 2015 Sep 4;11(9):e1004478. doi: 10.1371/journal.pcbi.1004478 (PMC4560400; doi:10.1371/journal.pcbi.1004478)
Supplement: S2 Material — (ZIP) [file pcbi.1004478.s002.zip › literature/SMc05009.pdf]

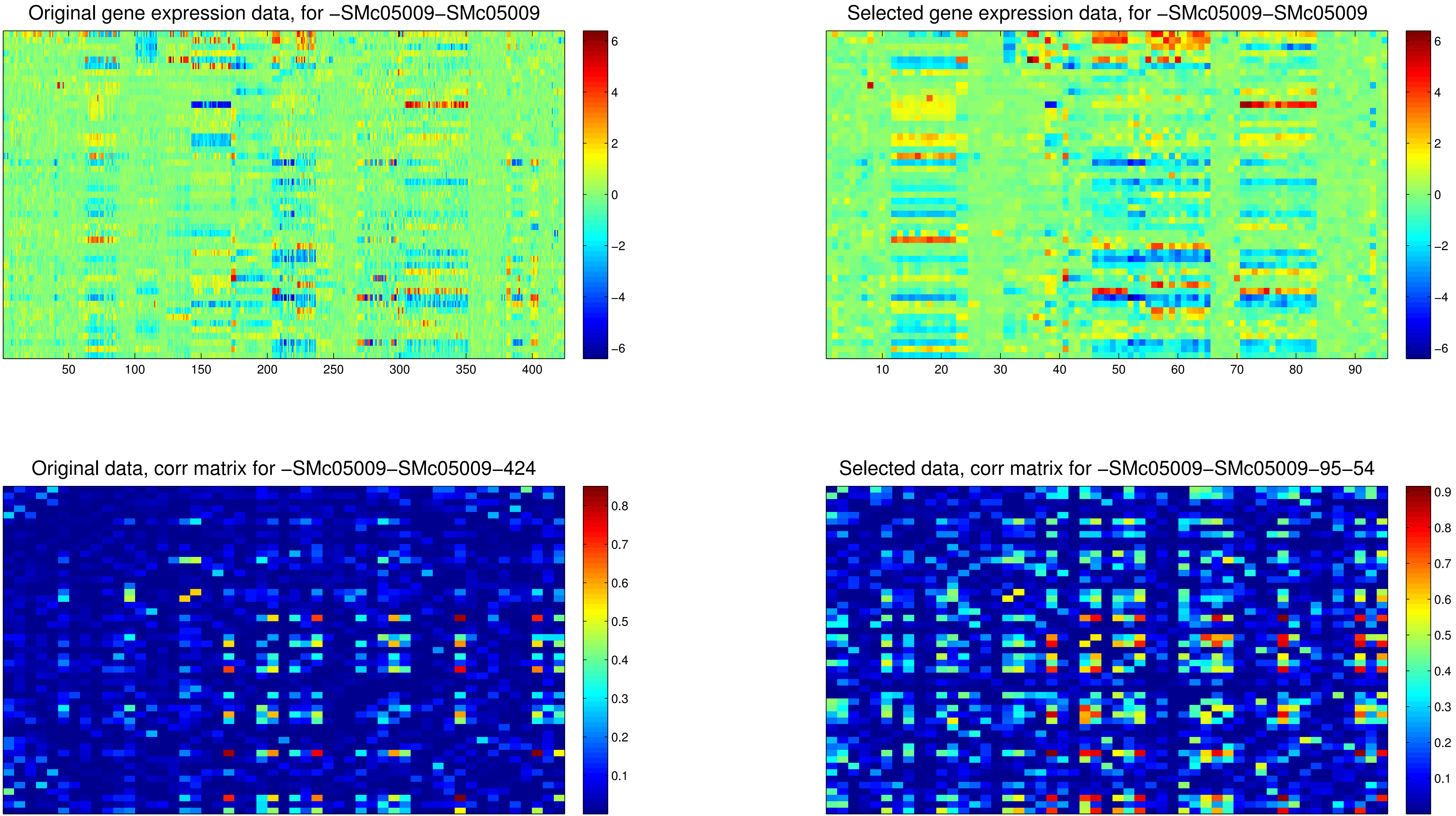
Original gene expression data, for –SMc05009–SMc05009
50
100
150
200
250
300
350
400
6
4
2
0
−2
−4
−6
Selected gene expression data, for –SMc05009–SMc05009
10
20
30
40
50
60
70
80
90
Original data, corr matrix for –SMc05009–SMc05009–424
0.8
0.7
0.6
0.5
0.4
0.3
0.2
0.1
Selected data, corr matrix for –SMc05009–SMc05009–95–54
0.9
