# Supplementary material for: Evolution of Intra-specific Regulatory Networks in a Multipartite Bacterial Genome
Source: PLoS Comput Biol. 2015 Sep 4;11(9):e1004478. doi: 10.1371/journal.pcbi.1004478 (PMC4560400; doi:10.1371/journal.pcbi.1004478)
Supplement: S2 Material — (ZIP) [file pcbi.1004478.s002.zip › literature/SMc02560.pdf]

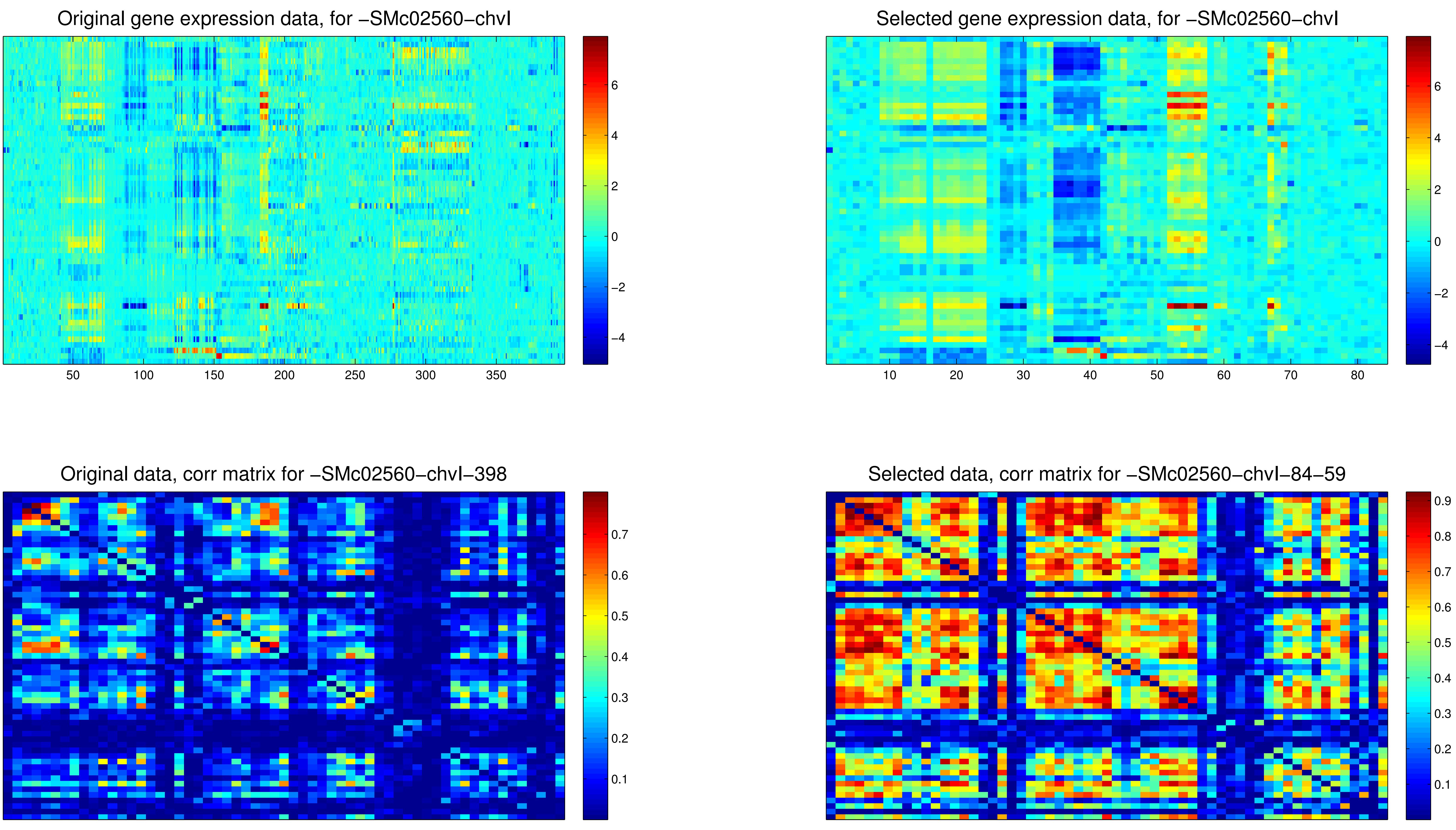
Original gene expression data, for −SMc02560−chvI
50 100 150 200 250 300 350
6 4 2 0 −2 −4
Selected gene expression data, for −SMc02560−chvI
10 20 30 40 50 60 70 80
6 4 2 0 −2 −4
Original data, corr matrix for −SMc02560−chvI−398
0.7 0.6 0.5 0.4 0.3 0.2 0.1
Selected data, corr matrix for −SMc02560−chvI−84−59
0.9 0.8 0.7 0.6 0.5 0.4 0.3 0.2 0.1
