# Supplementary material for: Evolution of Intra-specific Regulatory Networks in a Multipartite Bacterial Genome
Source: PLoS Comput Biol. 2015 Sep 4;11(9):e1004478. doi: 10.1371/journal.pcbi.1004478 (PMC4560400; doi:10.1371/journal.pcbi.1004478)
Supplement: S2 Material — (ZIP) [file pcbi.1004478.s002.zip › literature/SMc02140.pdf]

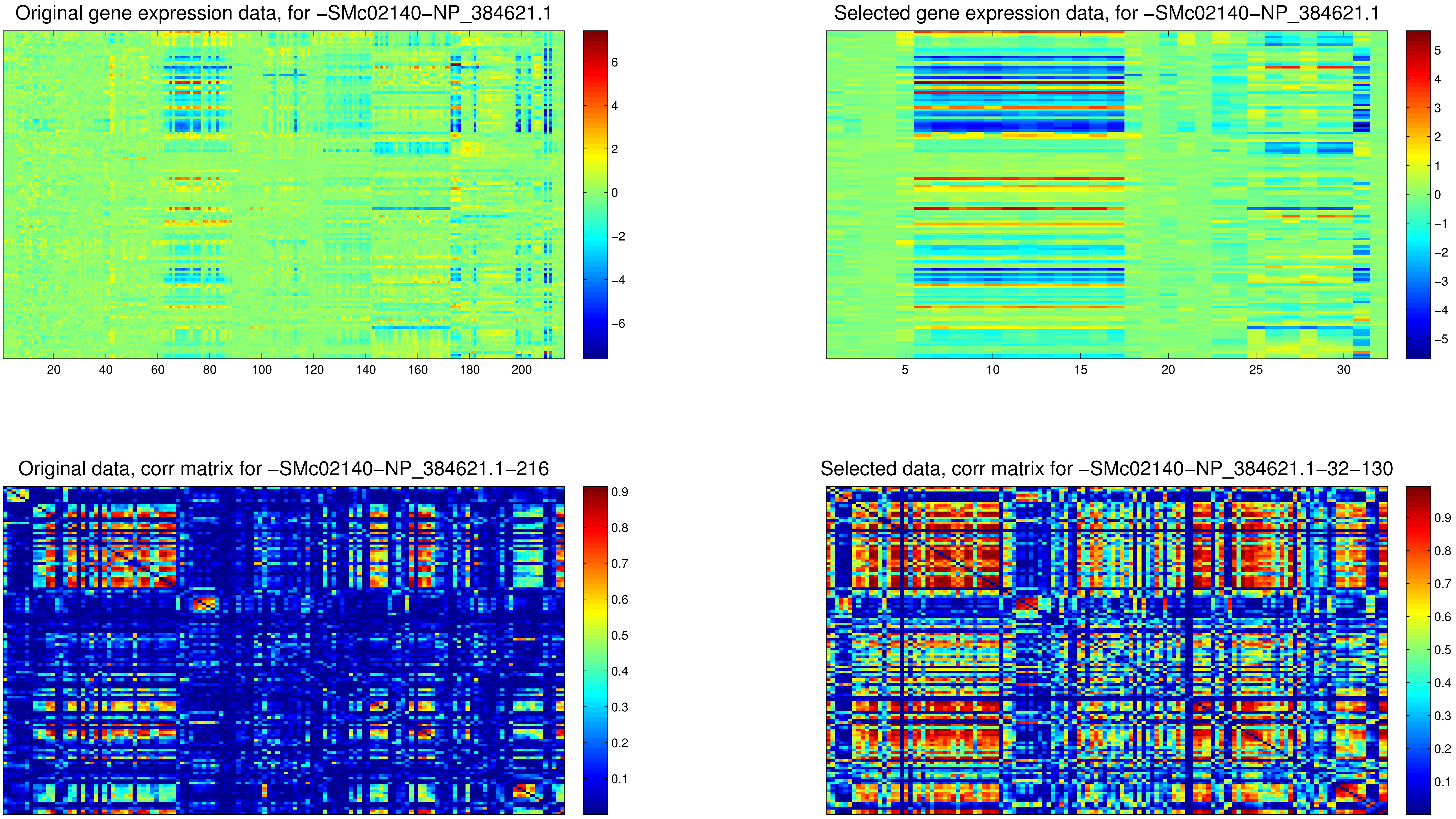

Original gene expression data, for −SMc02140−NP_384621.1
20 40 60 80 100 120 140 160 180 200
6 4 2 0 −2 −4 −6
Selected gene expression data, for −SMc02140−NP_384621.1
5 10 15 20 25 30
5 4 3 2 1 0 −1 −2 −3 −4 −5
Original data, corr matrix for −SMc02140−NP_384621.1−216
0.9 0.8 0.7 0.6 0.5 0.4 0.3 0.2 0.1
Selected data, corr matrix for −SMc02140−NP_384621.1−32−130
0.9 0.8 0.7 0.6 0.5 0.4 0.3 0.2 0.1
