# Supplementary material for: Evolution of Intra-specific Regulatory Networks in a Multipartite Bacterial Genome
Source: PLoS Comput Biol. 2015 Sep 4;11(9):e1004478. doi: 10.1371/journal.pcbi.1004478 (PMC4560400; doi:10.1371/journal.pcbi.1004478)
Supplement: S2 Material — (ZIP) [file pcbi.1004478.s002.zip › literature/SMc01506.pdf]

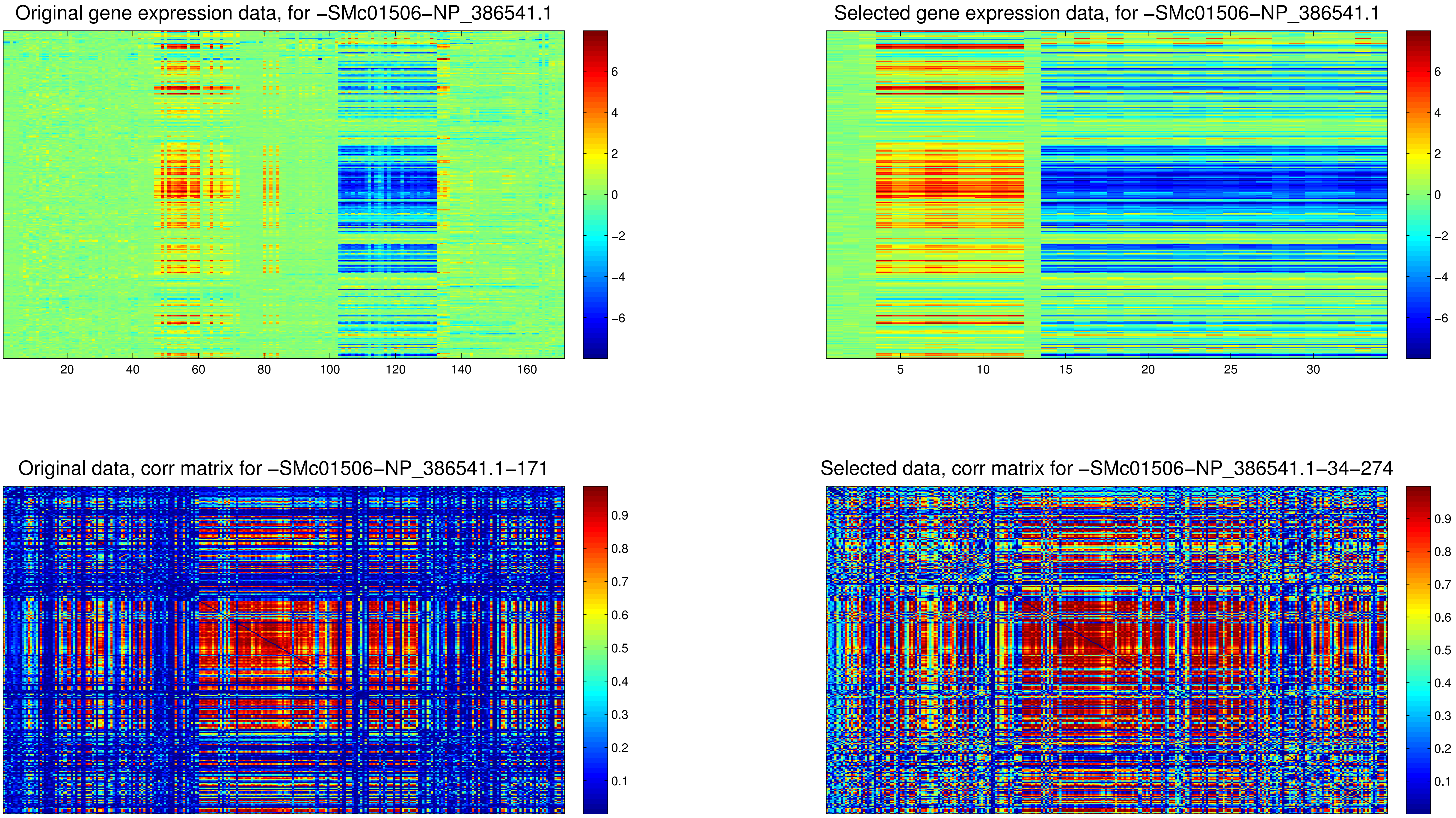
Original gene expression data, for −SMc01506−NP_386541.1
Selected gene expression data, for −SMc01506−NP_386541.1
Original data, corr matrix for −SMc01506−NP_386541.1−171
Selected data, corr matrix for −SMc01506−NP_386541.1−34−274
20 40 60 80 100 120 140 160
5 10 15 20 25 30
6 4 2 0 −2 −4 −6
0.9 0.8 0.7 0.6 0.5 0.4 0.3 0.2 0.1
