# Supplementary material for: Evolution of Intra-specific Regulatory Networks in a Multipartite Bacterial Genome
Source: PLoS Comput Biol. 2015 Sep 4;11(9):e1004478. doi: 10.1371/journal.pcbi.1004478 (PMC4560400; doi:10.1371/journal.pcbi.1004478)
Supplement: S2 Material — (ZIP) [file pcbi.1004478.s002.zip › literature/SMc01139.pdf]

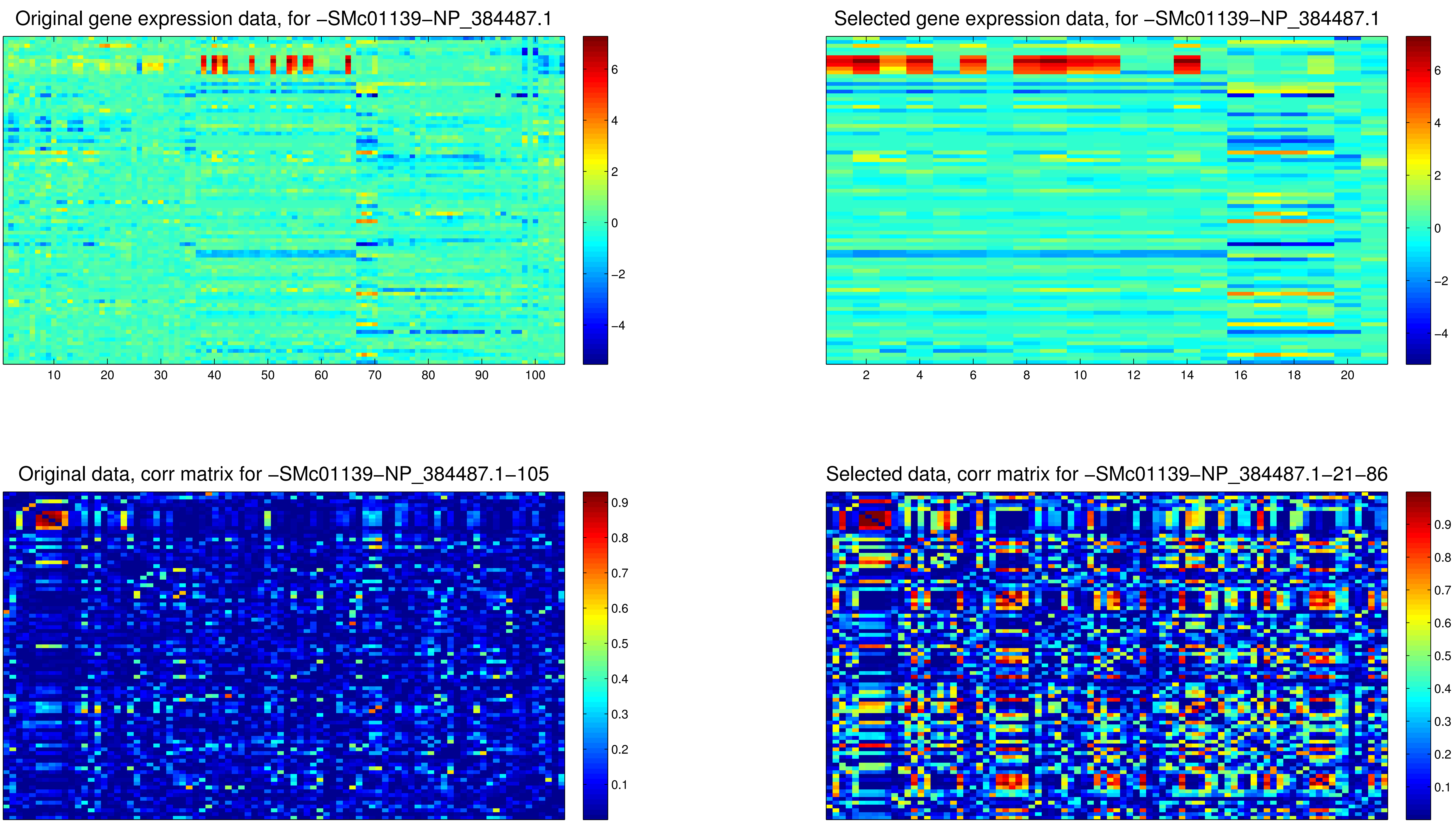
Original gene expression data, for −SMc01139−NP_384487.1
10 20 30 40 50 60 70 80 90 100
6 4 2 0 −2 −4
Selected gene expression data, for −SMc01139−NP_384487.1
2 4 6 8 10 12 14 16 18 20
6 4 2 0 −2 −4
Original data, corr matrix for −SMc01139−NP_384487.1−105
0.9 0.8 0.7 0.6 0.5 0.4 0.3 0.2 0.1
Selected data, corr matrix for −SMc01139−NP_384487.1−21−86
0.9 0.8 0.7 0.6 0.5 0.4 0.3 0.2 0.1
