# Supplementary material for: Evolution of Intra-specific Regulatory Networks in a Multipartite Bacterial Genome
Source: PLoS Comput Biol. 2015 Sep 4;11(9):e1004478. doi: 10.1371/journal.pcbi.1004478 (PMC4560400; doi:10.1371/journal.pcbi.1004478)

Original gene expression data, for –SMc00170–NP_385944.1

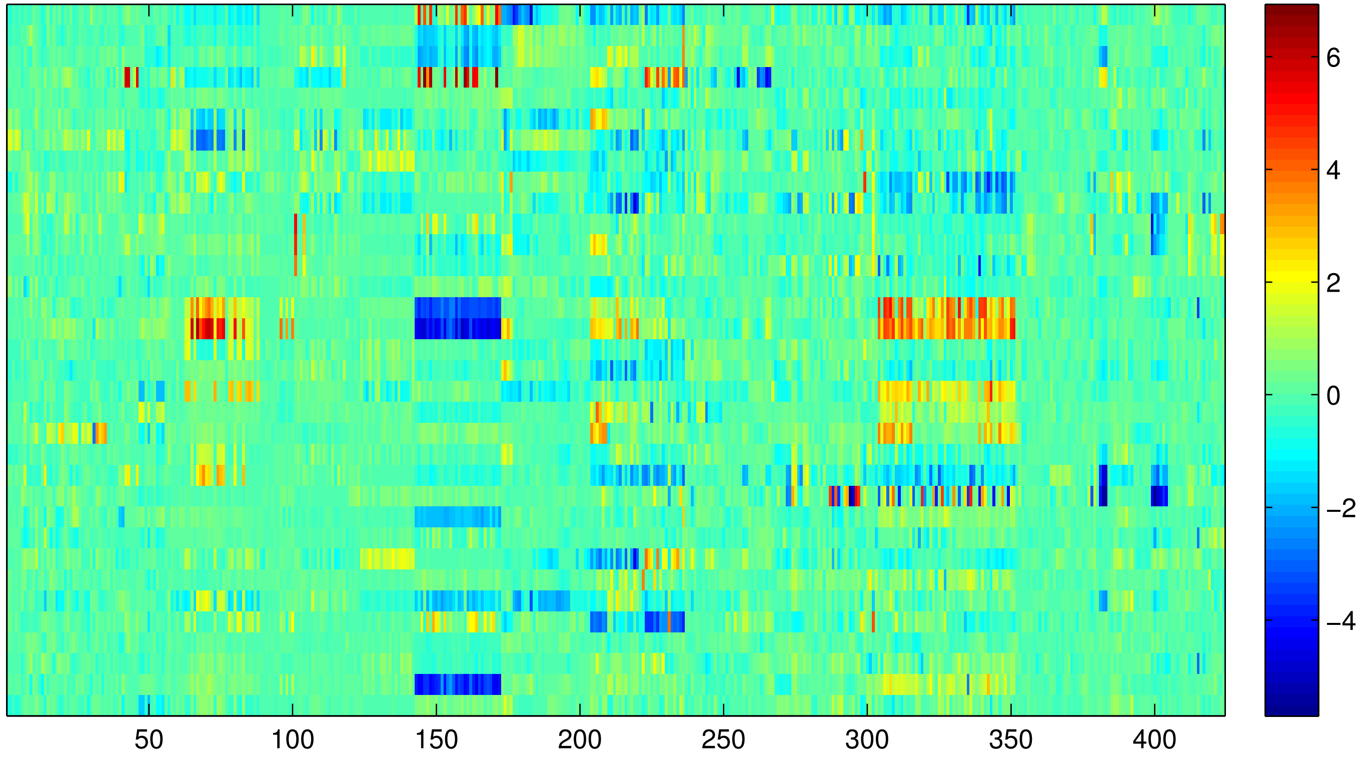

Selected gene expression data, for –SMc00170–NP_385944.1

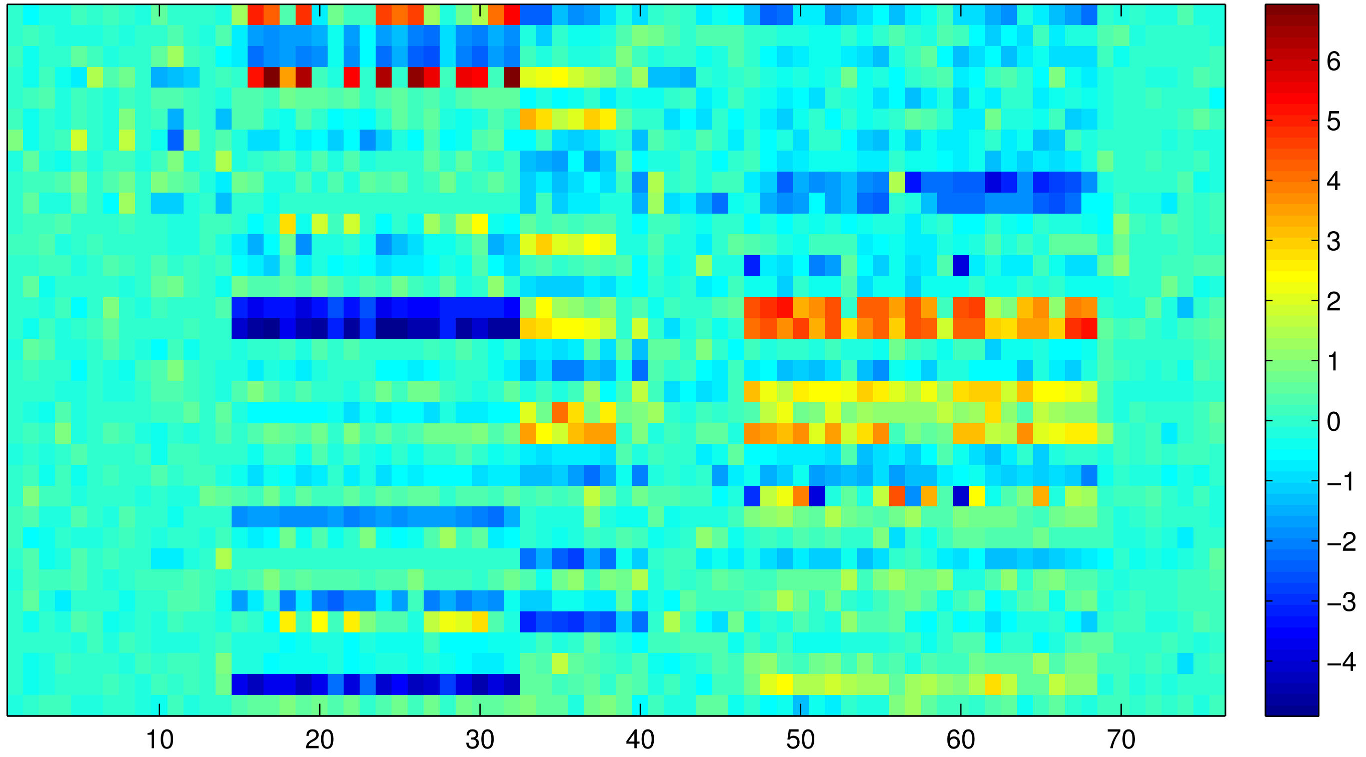

Original data, corr matrix for –SMc00170–NP_385944.1–424

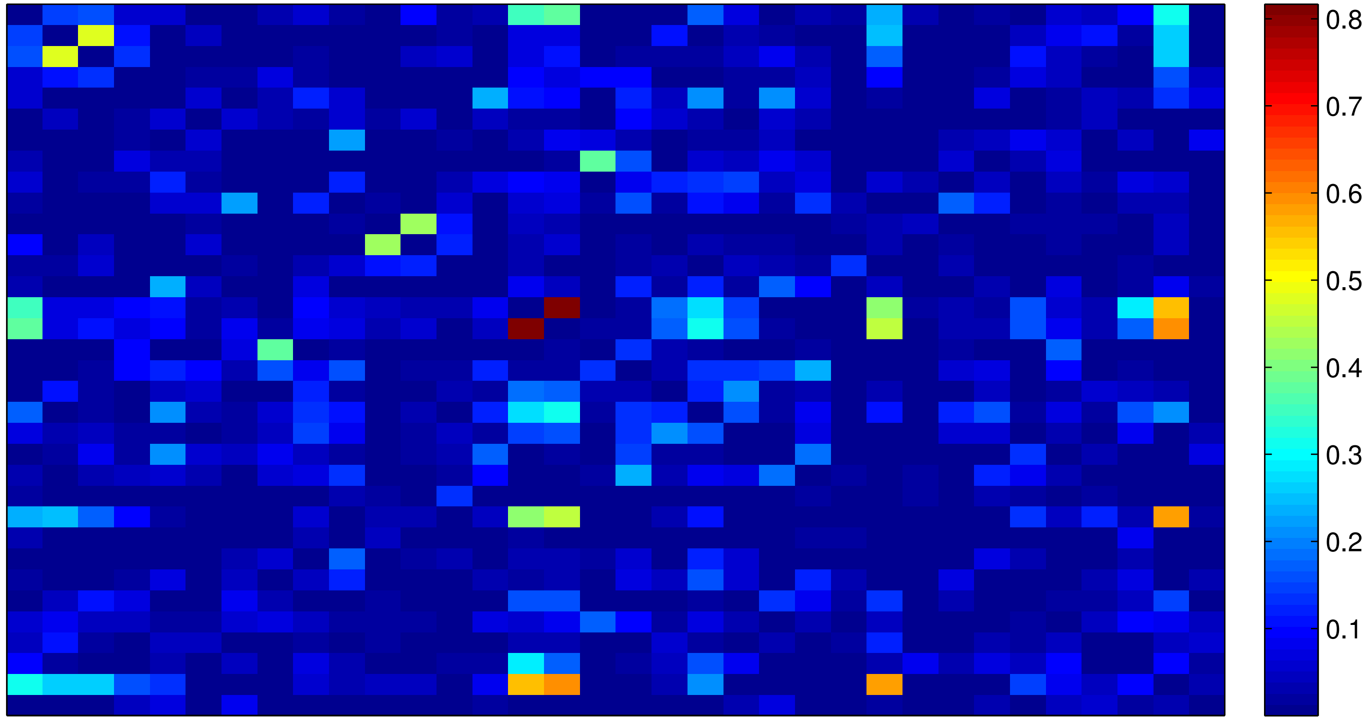

Selected data, corr matrix for –SMc00170–NP_385944.1–76–34

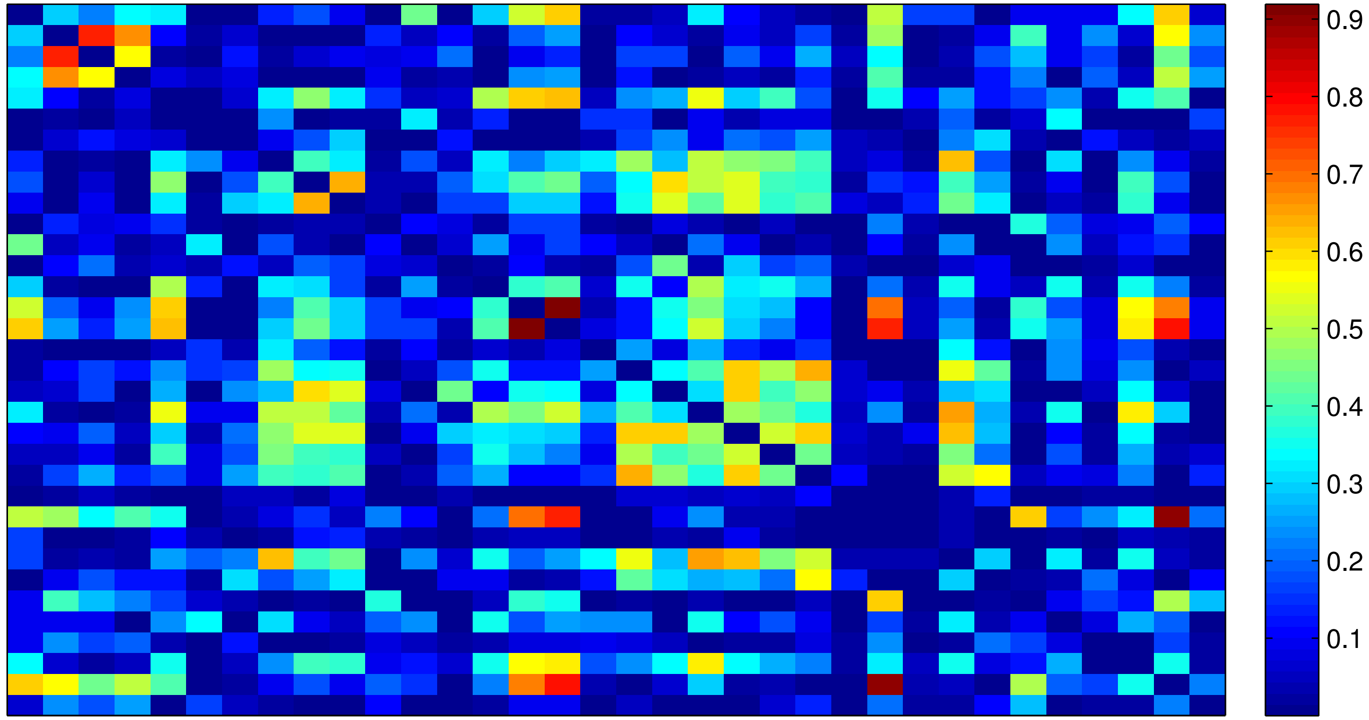

Supplement: S2 Material — (ZIP) [file pcbi.1004478.s002.zip › literature/SMc00170.pdf]
