# Supplementary material for: Evolution of Intra-specific Regulatory Networks in a Multipartite Bacterial Genome
Source: PLoS Comput Biol. 2015 Sep 4;11(9):e1004478. doi: 10.1371/journal.pcbi.1004478 (PMC4560400; doi:10.1371/journal.pcbi.1004478)
Supplement: S2 Material — (ZIP) [file pcbi.1004478.s002.zip › literature/SMc00058.pdf]

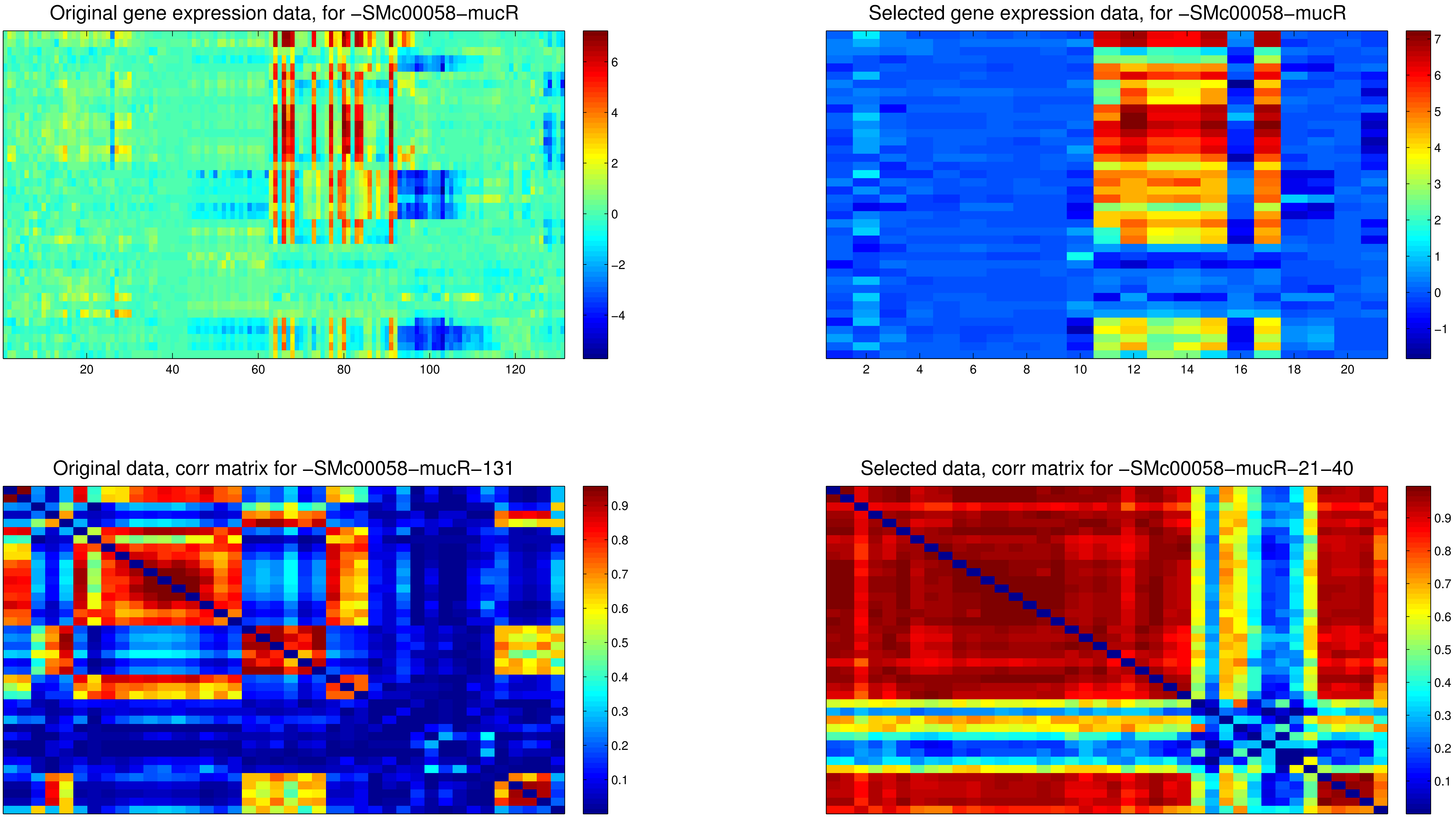
Original gene expression data, for −SMc00058−mucR
20
40
60
80
100
120
6
4
2
0
−2
−4
Selected gene expression data, for −SMc00058−mucR
2
4
6
8
10
12
14
16
18
20
7
6
5
4
3
2
1
0
−1
Original data, corr matrix for −SMc00058−mucR−131
0.9
0.8
0.7
0.6
0.5
0.4
0.3
0.2
0.1
Selected data, corr matrix for −SMc00058−mucR−21−40
0.9
0.8
0.7
0.6
0.5
0.4
0.3
0.2
0.1
