# Supplementary material for: Evolution of Intra-specific Regulatory Networks in a Multipartite Bacterial Genome
Source: PLoS Comput Biol. 2015 Sep 4;11(9):e1004478. doi: 10.1371/journal.pcbi.1004478 (PMC4560400; doi:10.1371/journal.pcbi.1004478)
Supplement: S2 Material — (ZIP) [file pcbi.1004478.s002.zip › literature/SMa0762.pdf]

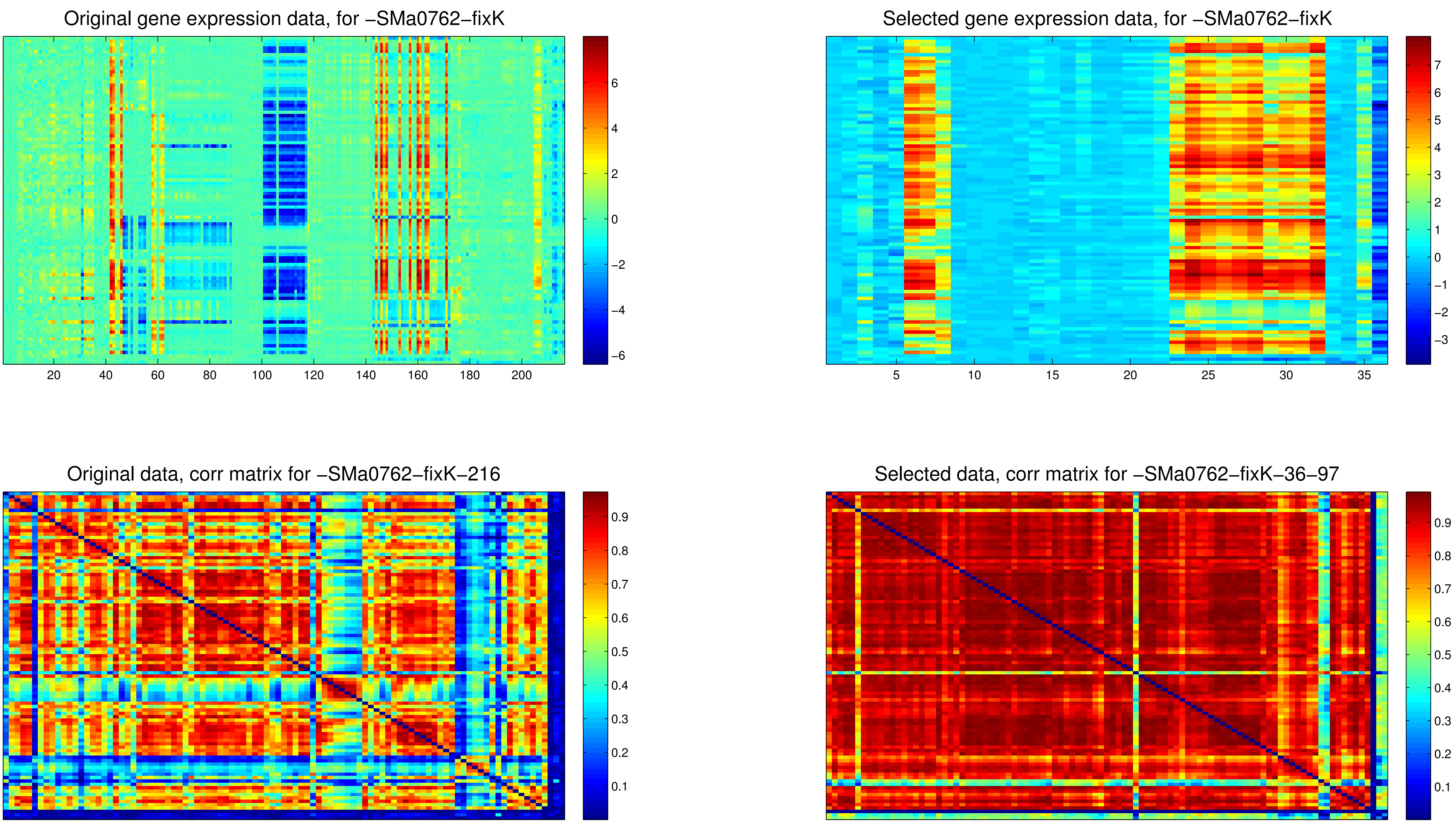
Original gene expression data, for −SMa0762−fixK
6
4
2
0
−2
−4
−6
20 40 60 80 100 120 140 160 180 200
Selected gene expression data, for −SMa0762−fixK
7
6
5
4
3
2
1
0
−1
−2
−3
5 10 15 20 25 30 35
Original data, corr matrix for −SMa0762−fixK−216
0.9
0.8
0.7
0.6
0.5
0.4
0.3
0.2
0.1
Selected data, corr matrix for −SMa0762−fixK−36−97
0.9
0.8
0.7
0.6
0.5
0.4
0.3
0.2
0.1
