# Supplementary material for: Evolution of Intra-specific Regulatory Networks in a Multipartite Bacterial Genome
Source: PLoS Comput Biol. 2015 Sep 4;11(9):e1004478. doi: 10.1371/journal.pcbi.1004478 (PMC4560400; doi:10.1371/journal.pcbi.1004478)
Supplement: S2 Material — (ZIP) [file pcbi.1004478.s002.zip › predictions/SMc04401.pdf]

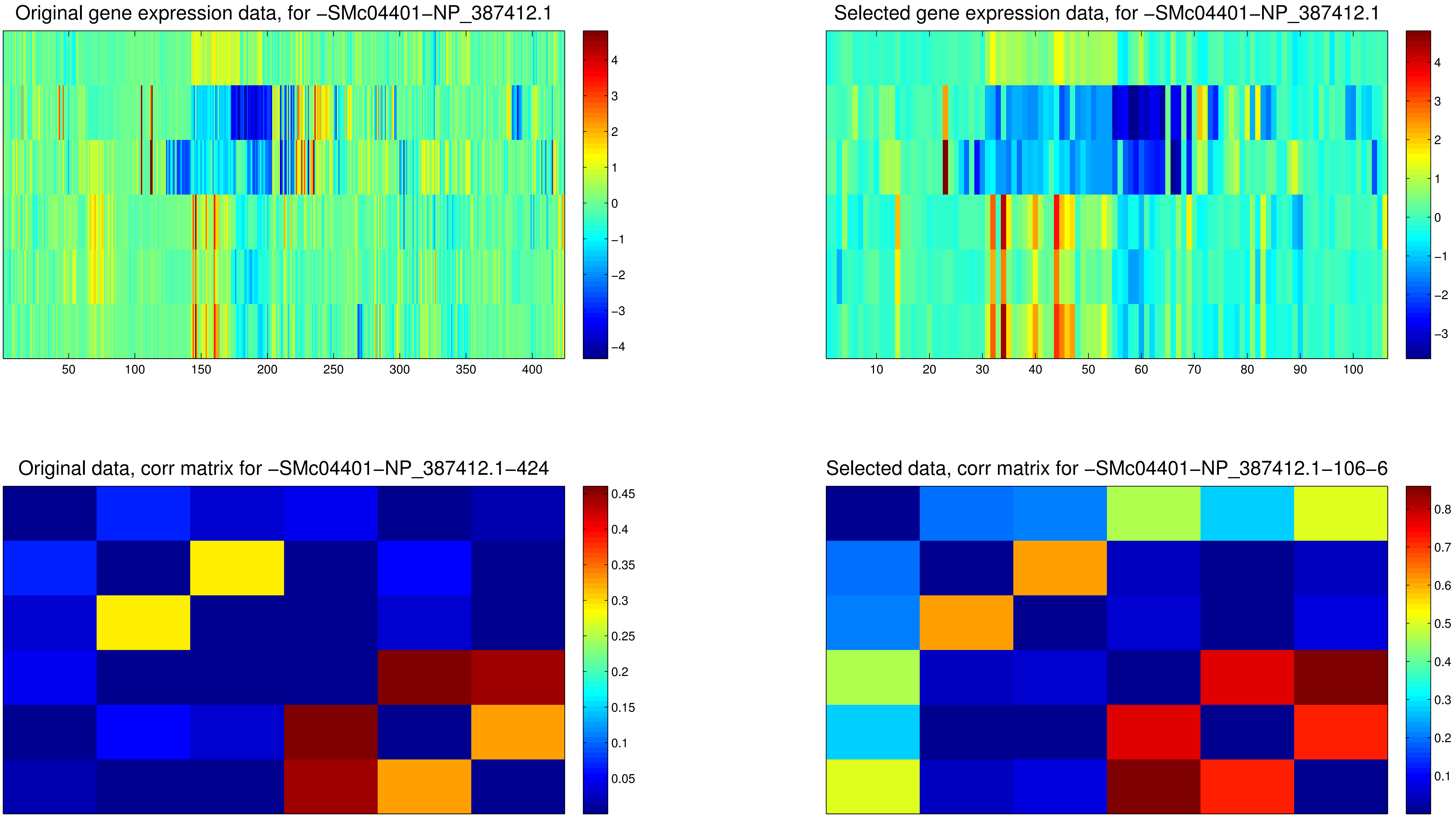
Original gene expression data, for −SMc04401−NP_387412.1
50 100 150 200 250 300 350 400
−4 −3 −2 −1 0 1 2 3 4
Selected gene expression data, for −SMc04401−NP_387412.1
10 20 30 40 50 60 70 80 90 100
−3 −2 −1 0 1 2 3 4
Original data, corr matrix for −SMc04401−NP_387412.1−424
0.05 0.1 0.15 0.2 0.25 0.3 0.35 0.4 0.45
Selected data, corr matrix for −SMc04401−NP_387412.1−106−6
0.1 0.2 0.3 0.4 0.5 0.6 0.7 0.8
