# Supplementary material for: Evolution of Intra-specific Regulatory Networks in a Multipartite Bacterial Genome
Source: PLoS Comput Biol. 2015 Sep 4;11(9):e1004478. doi: 10.1371/journal.pcbi.1004478 (PMC4560400; doi:10.1371/journal.pcbi.1004478)
Supplement: S2 Material — (ZIP) [file pcbi.1004478.s002.zip › predictions/SMc03806.pdf]

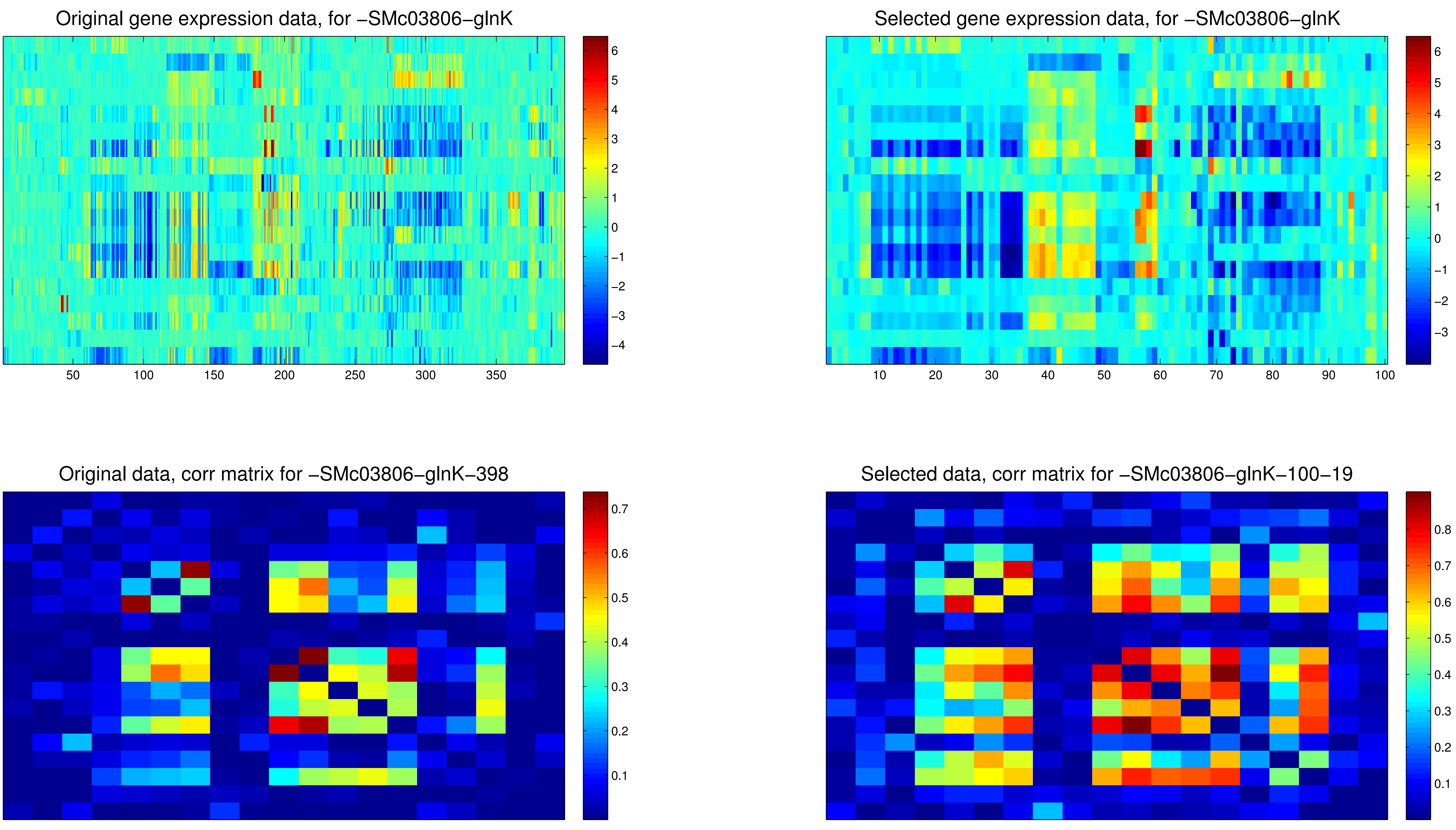
Original gene expression data, for −SMc03806−glnK
Selected gene expression data, for −SMc03806−glnK
Original data, corr matrix for −SMc03806−glnK−398
Selected data, corr matrix for −SMc03806−glnK−100−19
50 100 150 200 250 300 350
10 20 30 40 50 60 70 80 90 100
6 5 4 3 2 1 0 −1 −2 −3 −4
6 5 4 3 2 1 0 −1 −2 −3
0.7 0.6 0.5 0.4 0.3 0.2 0.1
0.8 0.7 0.6 0.5 0.4 0.3 0.2 0.1
