# Supplementary material for: Evolution of Intra-specific Regulatory Networks in a Multipartite Bacterial Genome
Source: PLoS Comput Biol. 2015 Sep 4;11(9):e1004478. doi: 10.1371/journal.pcbi.1004478 (PMC4560400; doi:10.1371/journal.pcbi.1004478)
Supplement: S2 Material — (ZIP) [file pcbi.1004478.s002.zip › predictions/SMc03210.pdf]

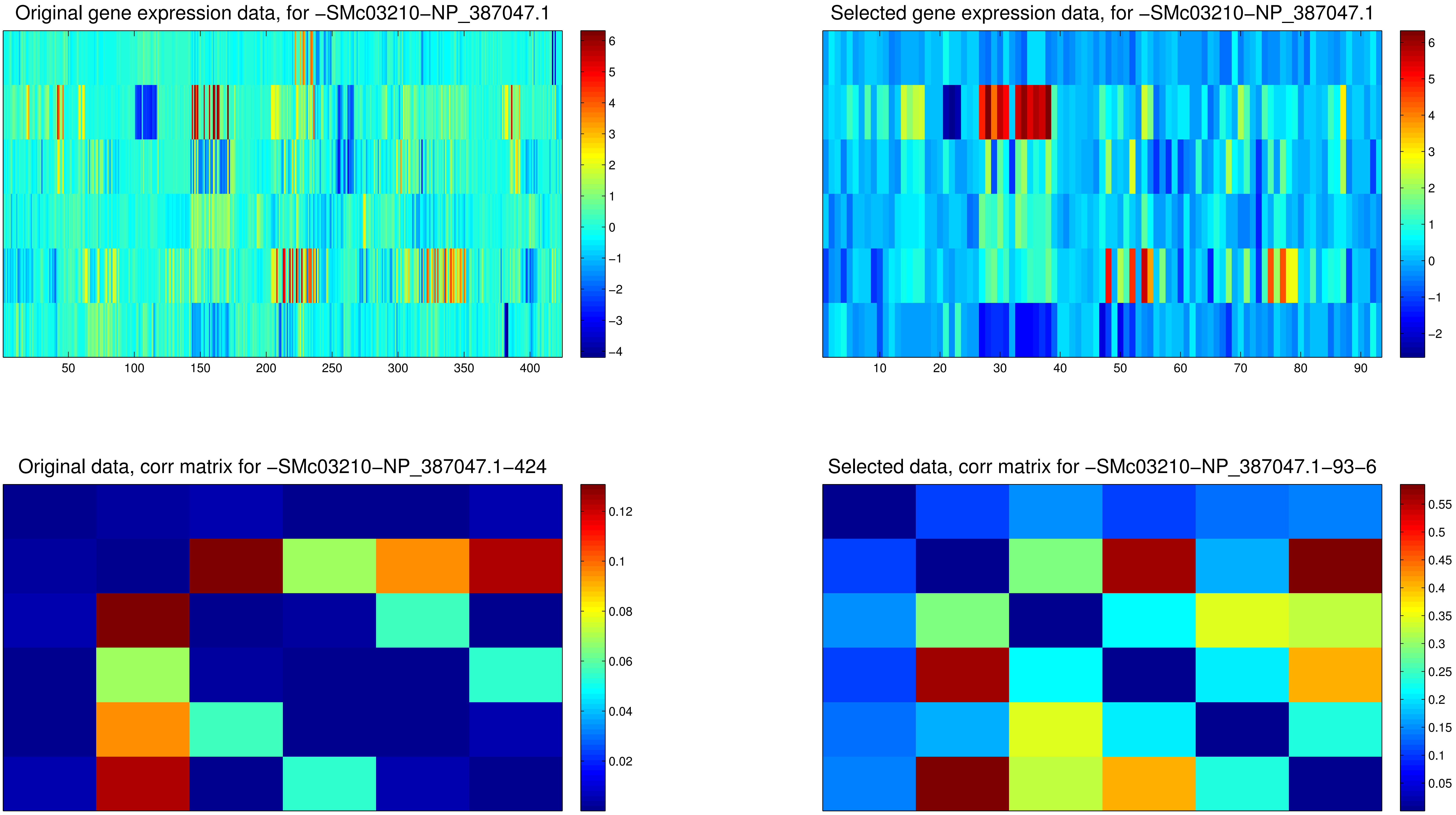
Original gene expression data, for –SMc03210–NP_387047.1
50 100 150 200 250 300 350 400
−4 −3 −2 −1 0 1 2 3 4 5 6
Selected gene expression data, for –SMc03210–NP_387047.1
10 20 30 40 50 60 70 80 90
−2 −1 0 1 2 3 4 5 6
Original data, corr matrix for –SMc03210–NP_387047.1–424
0.02 0.04 0.06 0.08 0.1 0.12
Selected data, corr matrix for –SMc03210–NP_387047.1–93–6
0.05 0.1 0.15 0.2 0.25 0.3 0.35 0.4 0.45 0.5 0.55
