# Supplementary material for: Evolution of Intra-specific Regulatory Networks in a Multipartite Bacterial Genome
Source: PLoS Comput Biol. 2015 Sep 4;11(9):e1004478. doi: 10.1371/journal.pcbi.1004478 (PMC4560400; doi:10.1371/journal.pcbi.1004478)

Original gene expression data, for –SMc00785–NP_384836.1

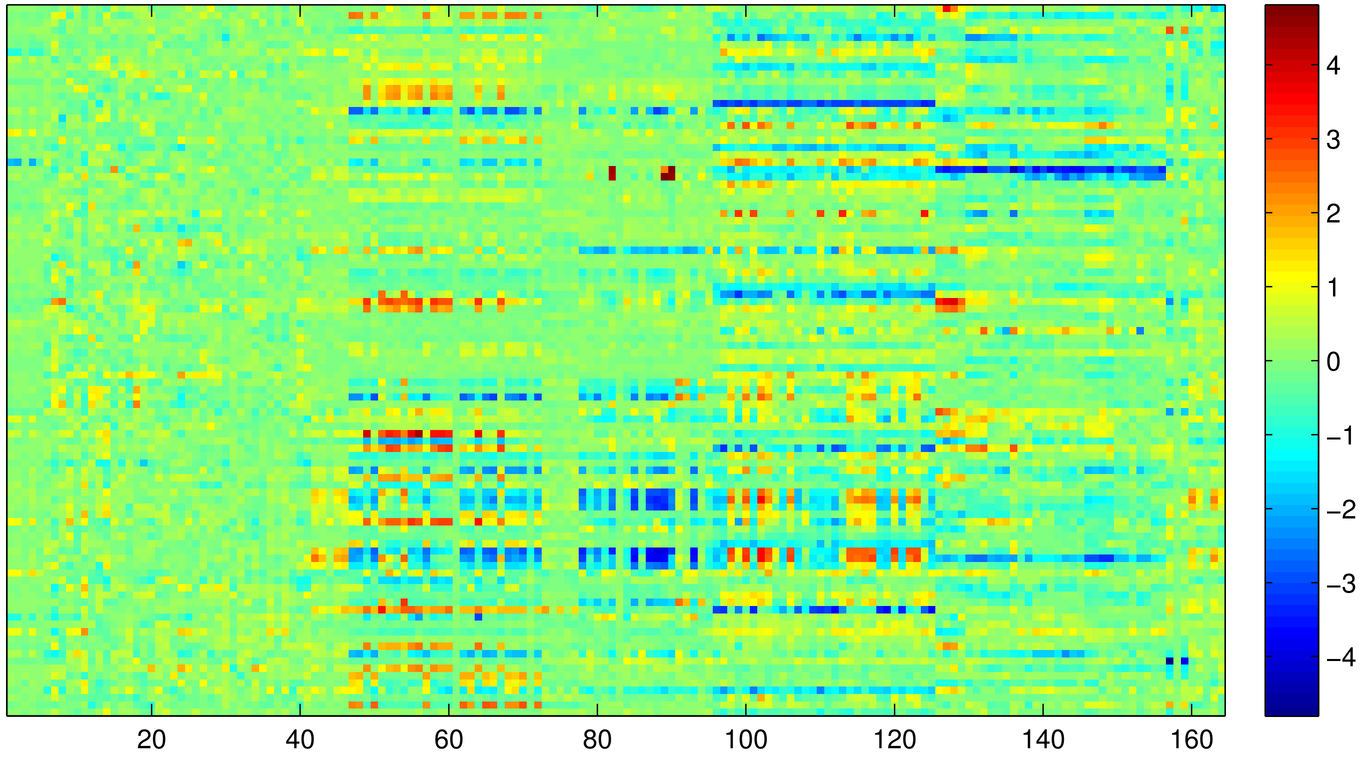

Selected gene expression data, for –SMc00785–NP_384836.1

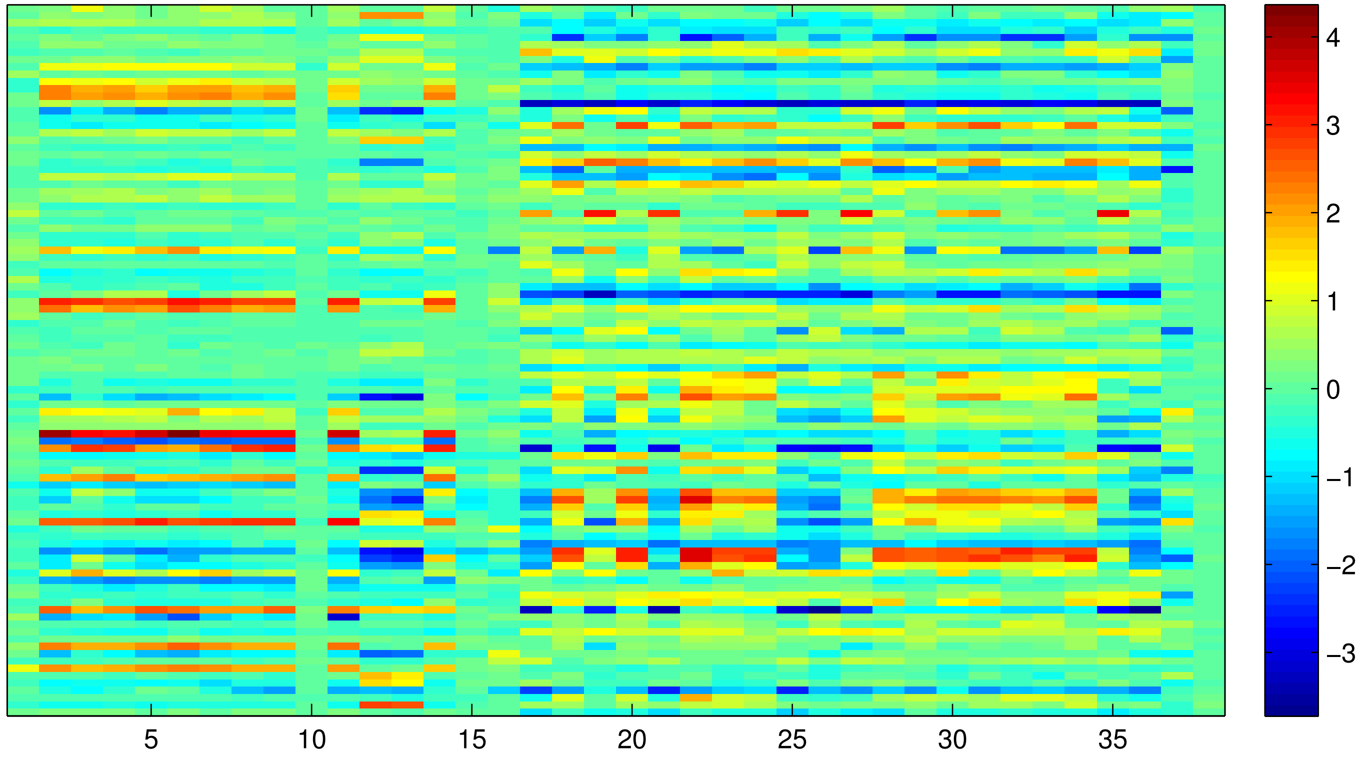

Original data, corr matrix for –SMc00785–NP_384836.1–164

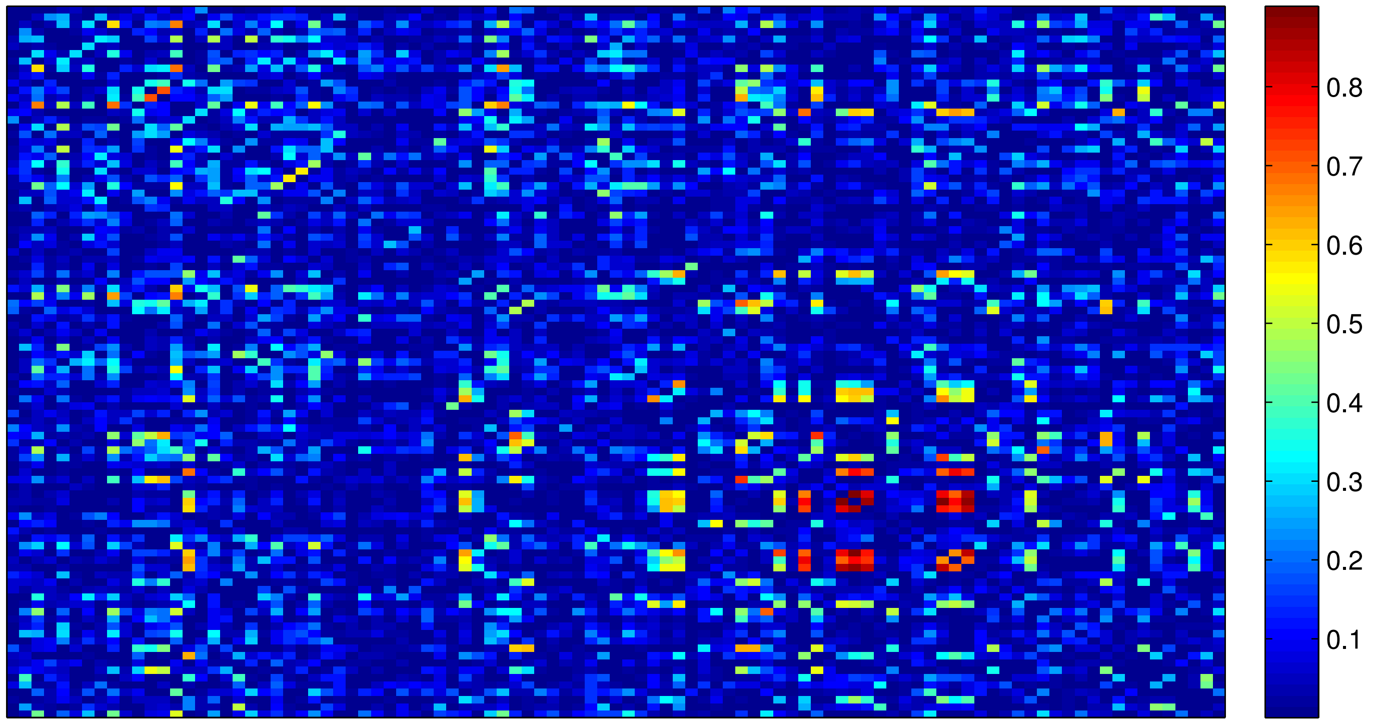

Selected data, corr matrix for –SMc00785–NP_384836.1–38–98

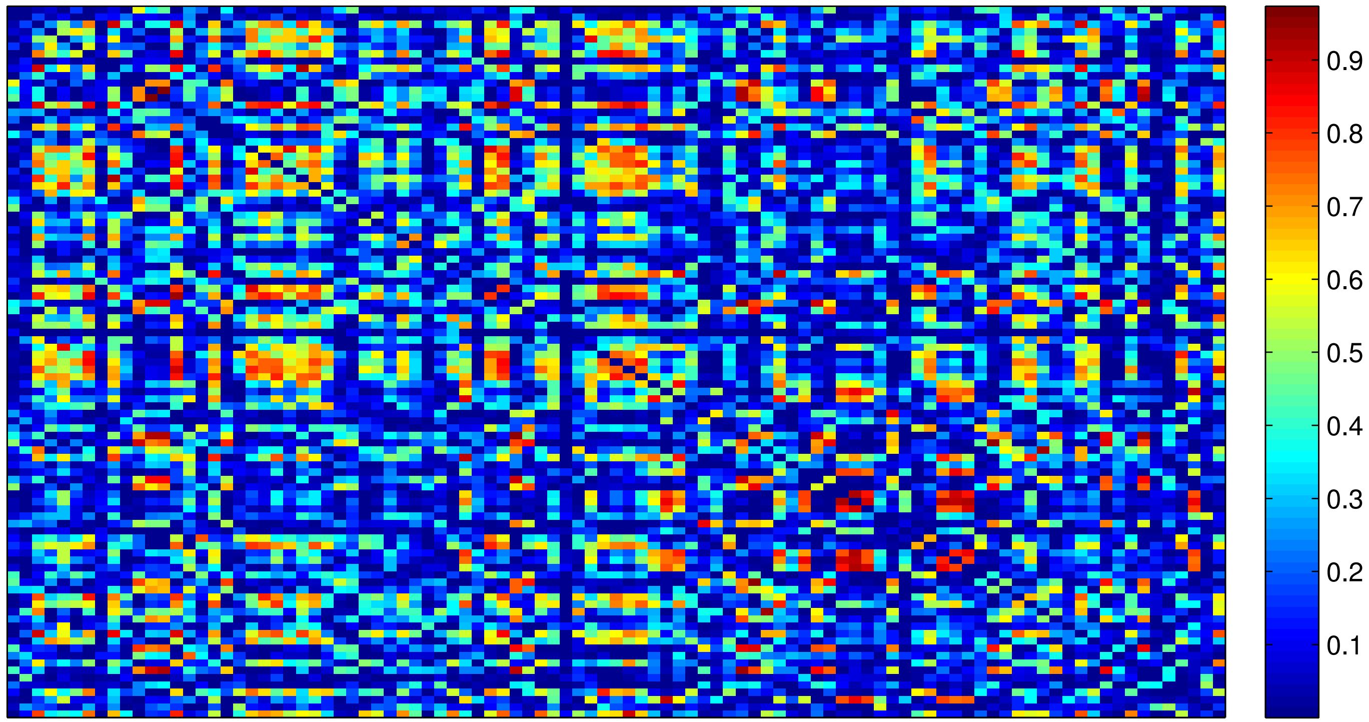

Supplement: S2 Material — (ZIP) [file pcbi.1004478.s002.zip › predictions/SMc00785.pdf]
