# Supplementary material for: Evolution of Intra-specific Regulatory Networks in a Multipartite Bacterial Genome
Source: PLoS Comput Biol. 2015 Sep 4;11(9):e1004478. doi: 10.1371/journal.pcbi.1004478 (PMC4560400; doi:10.1371/journal.pcbi.1004478)
Supplement: S2 Material — (ZIP) [file pcbi.1004478.s002.zip › predictions/SMb21706.pdf]

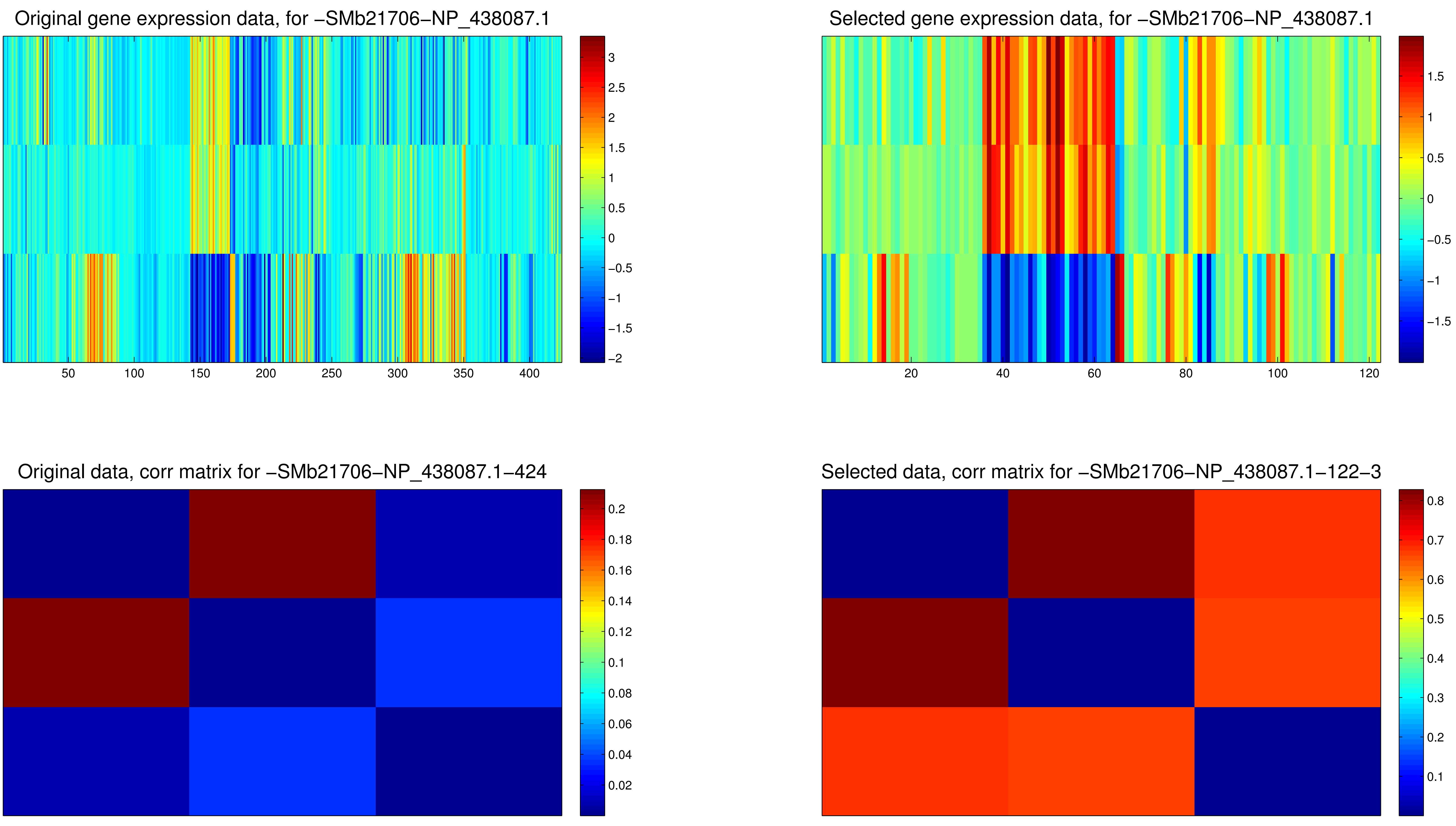
Original gene expression data, for −SMb21706−NP_438087.1
50 100 150 200 250 300 350 400
3 2.5 2 1.5 1 0.5 0 −0.5 −1 −1.5 −2
Selected gene expression data, for −SMb21706−NP_438087.1
20 40 60 80 100 120
1.5 1 0.5 0 −0.5 −1 −1.5
Original data, corr matrix for −SMb21706−NP_438087.1−424
0.2 0.18 0.16 0.14 0.12 0.1 0.08 0.06 0.04 0.02
Selected data, corr matrix for −SMb21706−NP_438087.1−122−3
0.8 0.7 0.6 0.5 0.4 0.3 0.2 0.1
