# Supplementary material for: Evolution of Intra-specific Regulatory Networks in a Multipartite Bacterial Genome
Source: PLoS Comput Biol. 2015 Sep 4;11(9):e1004478. doi: 10.1371/journal.pcbi.1004478 (PMC4560400; doi:10.1371/journal.pcbi.1004478)
Supplement: S2 Material — (ZIP) [file pcbi.1004478.s002.zip › predictions/SMb21598.pdf]

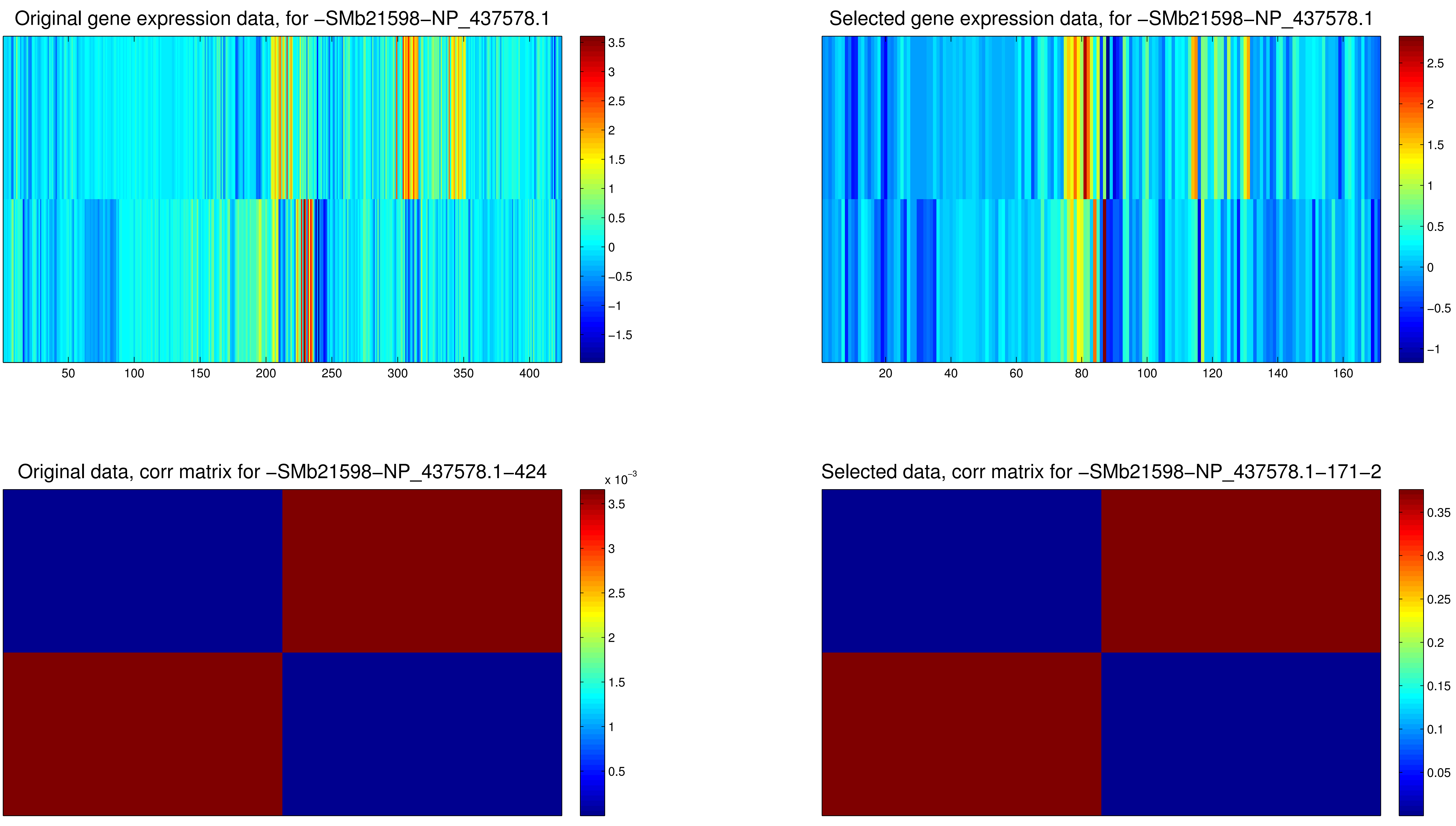
Original gene expression data, for −SMb21598−NP_437578.1
Selected gene expression data, for −SMb21598−NP_437578.1
Original data, corr matrix for −SMb21598−NP_437578.1−424
Selected data, corr matrix for −SMb21598−NP_437578.1−171−2
50
100
150
200
250
300
350
400
20
40
60
80
100
120
140
160
3.5
3
2.5
2
1.5
1
0.5
0
−0.5
−1
−1.5
x 10−3
0.35
0.3
0.25
0.2
0.15
0.1
0.05
