# Supplementary material for: Evolution of Intra-specific Regulatory Networks in a Multipartite Bacterial Genome
Source: PLoS Comput Biol. 2015 Sep 4;11(9):e1004478. doi: 10.1371/journal.pcbi.1004478 (PMC4560400; doi:10.1371/journal.pcbi.1004478)
Supplement: S2 Material — (ZIP) [file pcbi.1004478.s002.zip › predictions/SMb21372.pdf]

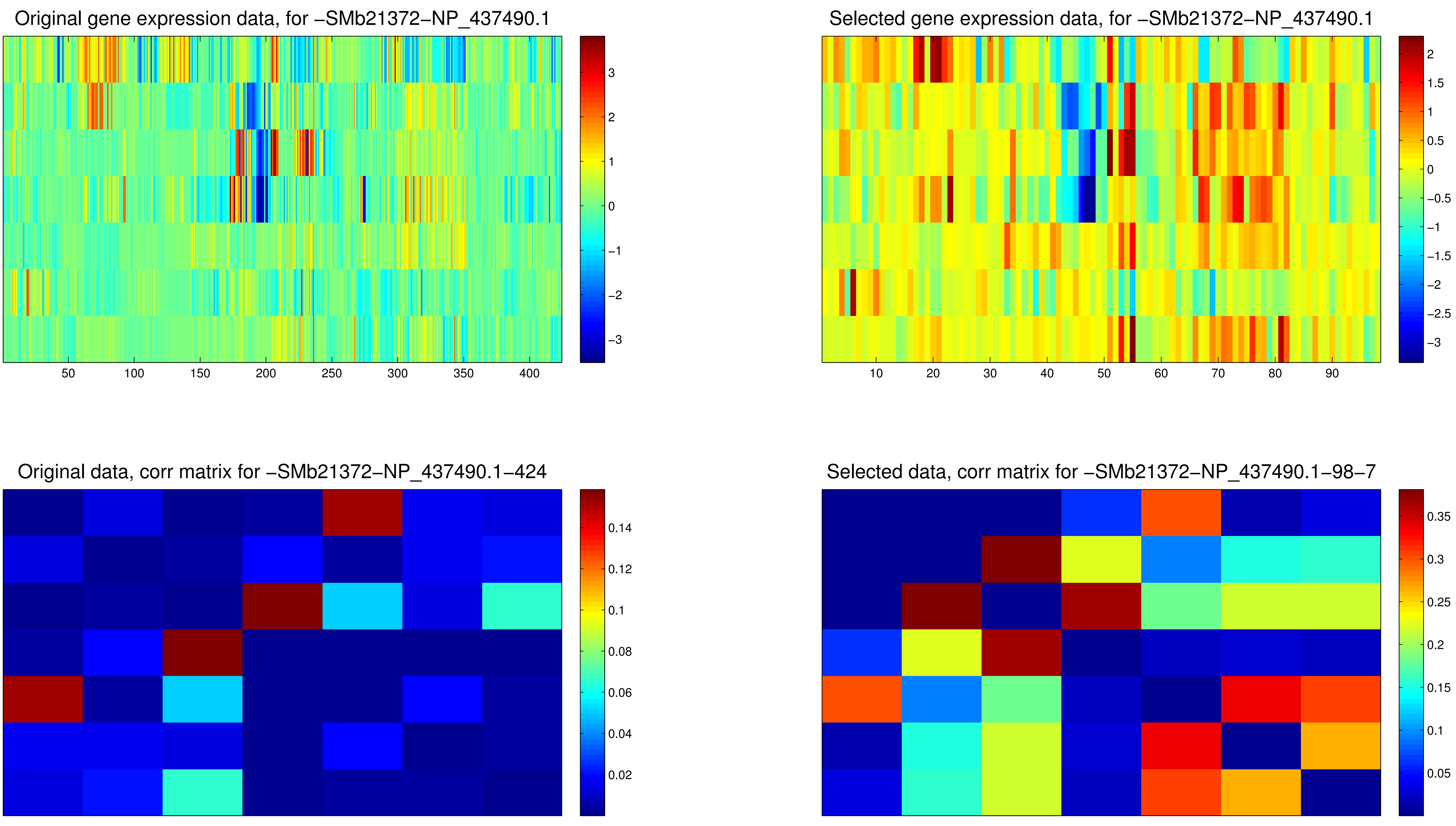
Original gene expression data, for −SMb21372−NP_437490.1
50 100 150 200 250 300 350 400
3 2 1 0 −1 −2 −3
Selected gene expression data, for −SMb21372−NP_437490.1
10 20 30 40 50 60 70 80 90
2 1.5 1 0.5 0 −0.5 −1 −1.5 −2 −2.5 −3
Original data, corr matrix for −SMb21372−NP_437490.1−424
0.14 0.12 0.1 0.08 0.06 0.04 0.02
Selected data, corr matrix for −SMb21372−NP_437490.1−98−7
0.35 0.3 0.25 0.2 0.15 0.1 0.05
