# Supplementary material for: Evolution of Intra-specific Regulatory Networks in a Multipartite Bacterial Genome
Source: PLoS Comput Biol. 2015 Sep 4;11(9):e1004478. doi: 10.1371/journal.pcbi.1004478 (PMC4560400; doi:10.1371/journal.pcbi.1004478)
Supplement: S2 Material — (ZIP) [file pcbi.1004478.s002.zip › predictions/SMb20667.pdf]

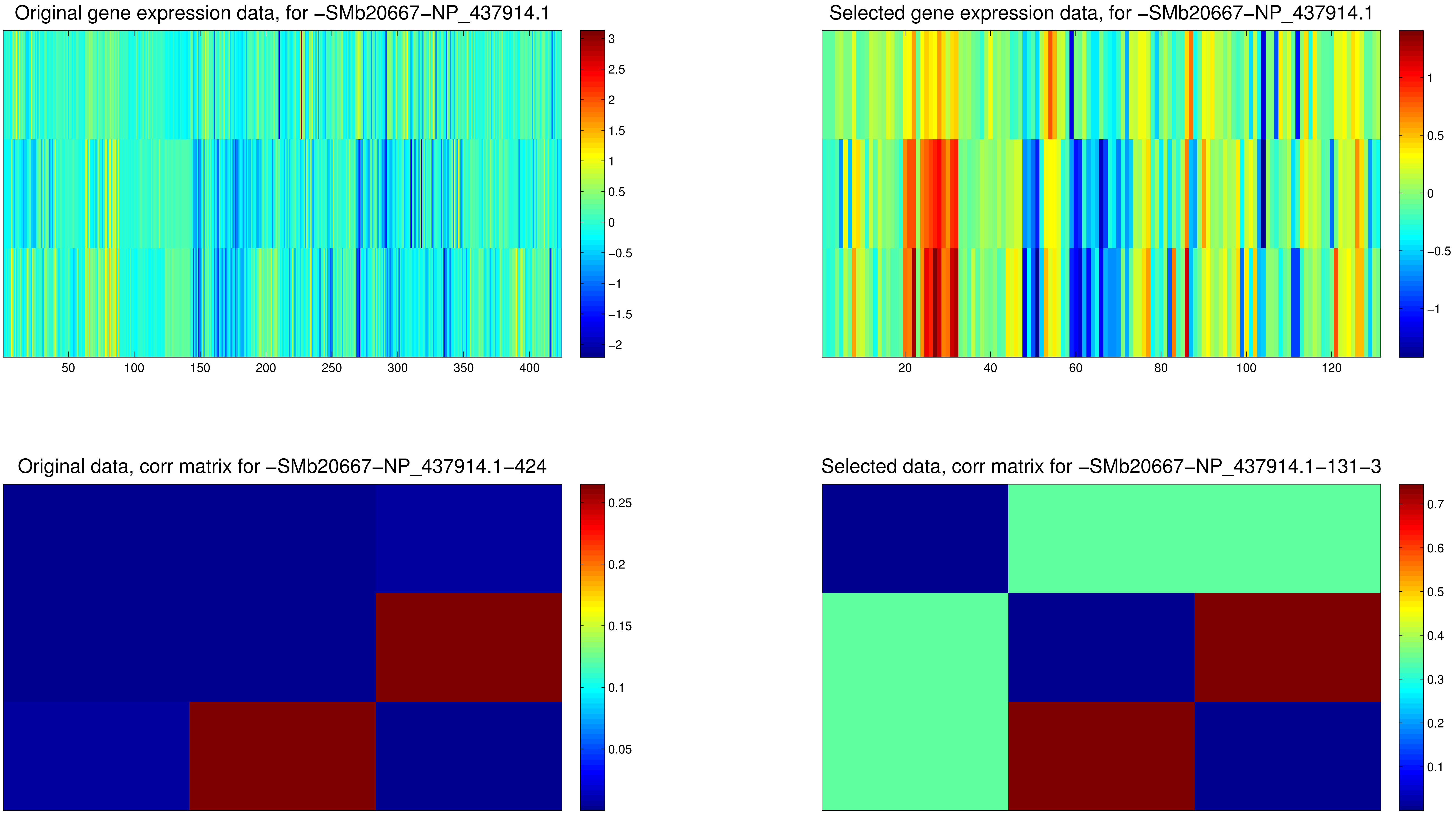
Original gene expression data, for −SMb20667−NP_437914.1
50
100
150
200
250
300
350
400
3
2.5
2
1.5
1
0.5
0
−0.5
−1
−1.5
−2
Selected gene expression data, for −SMb20667−NP_437914.1
20
40
60
80
100
120
1
0.5
0
−0.5
−1
Original data, corr matrix for −SMb20667−NP_437914.1−424
0.25
0.2
0.15
0.1
0.05
Selected data, corr matrix for −SMb20667−NP_437914.1−131−3
0.7
0.6
0.5
0.4
0.3
0.2
0.1
