# Supplementary material for: Evolution of Intra-specific Regulatory Networks in a Multipartite Bacterial Genome
Source: PLoS Comput Biol. 2015 Sep 4;11(9):e1004478. doi: 10.1371/journal.pcbi.1004478 (PMC4560400; doi:10.1371/journal.pcbi.1004478)
Supplement: S2 Material — (ZIP) [file pcbi.1004478.s002.zip › predictions/SMb20613.pdf]

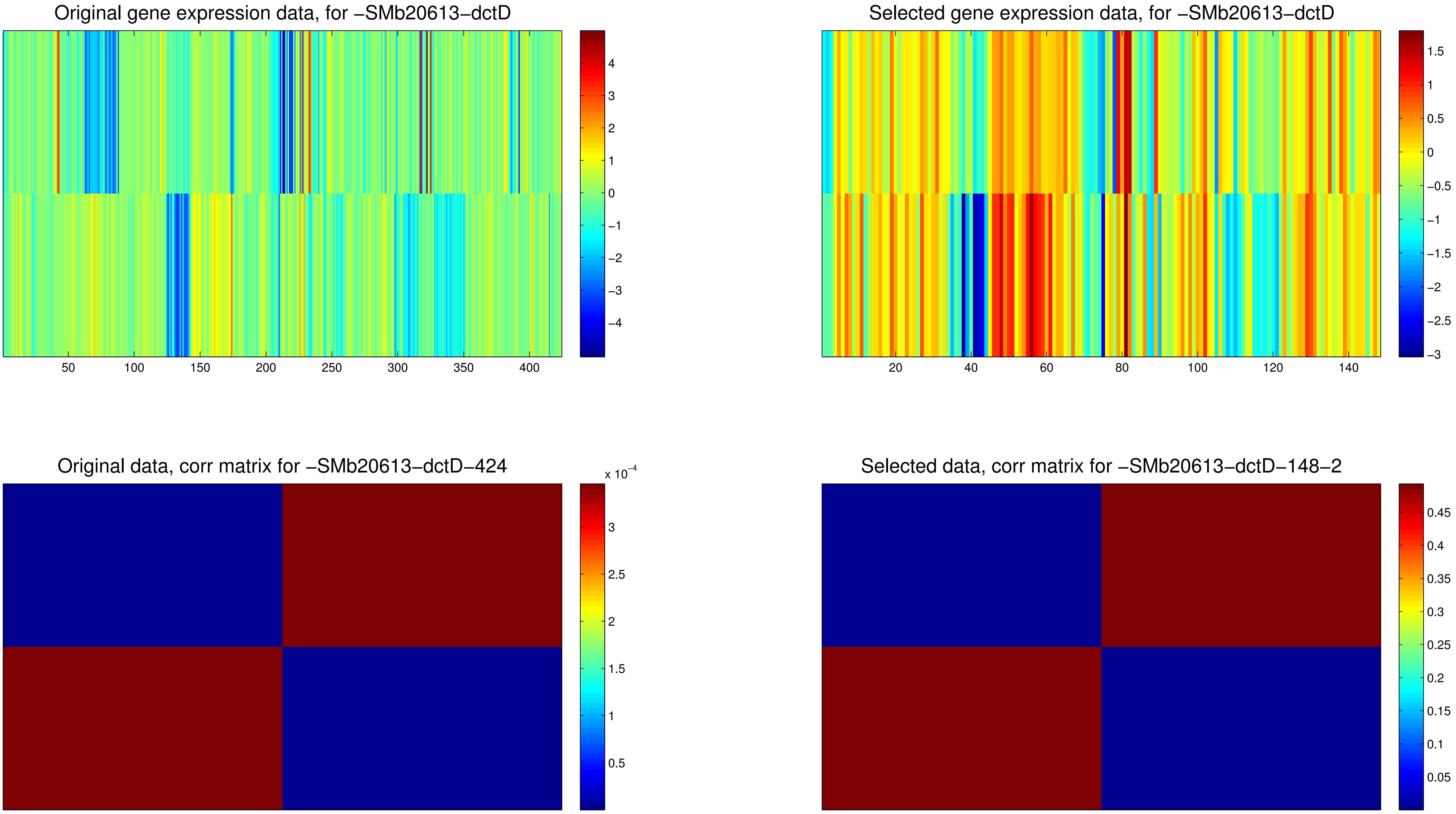
Original gene expression data, for −SMb20613−dctD
Selected gene expression data, for −SMb20613−dctD
Original data, corr matrix for −SMb20613−dctD−424
Selected data, corr matrix for −SMb20613−dctD−148−2
x 10^−4
