# Supplementary material for: Evolution of Intra-specific Regulatory Networks in a Multipartite Bacterial Genome
Source: PLoS Comput Biol. 2015 Sep 4;11(9):e1004478. doi: 10.1371/journal.pcbi.1004478 (PMC4560400; doi:10.1371/journal.pcbi.1004478)
Supplement: S2 Material — (ZIP) [file pcbi.1004478.s002.zip › predictions/SMb20324.pdf]

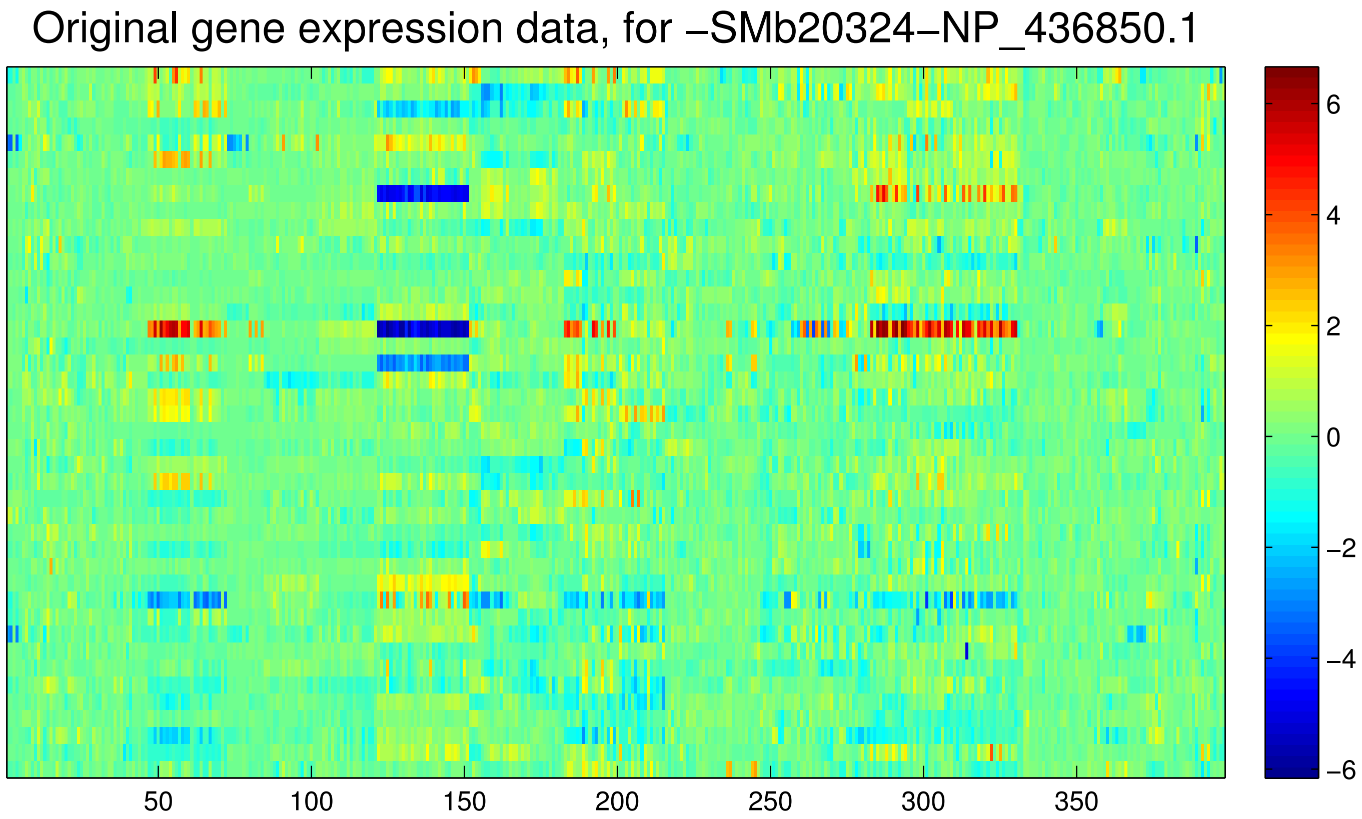
Original gene expression data, for −SMb20324−NP_436850.1
50
100
150
200
250
300
350
6
4
2
0
−2
−4
−6

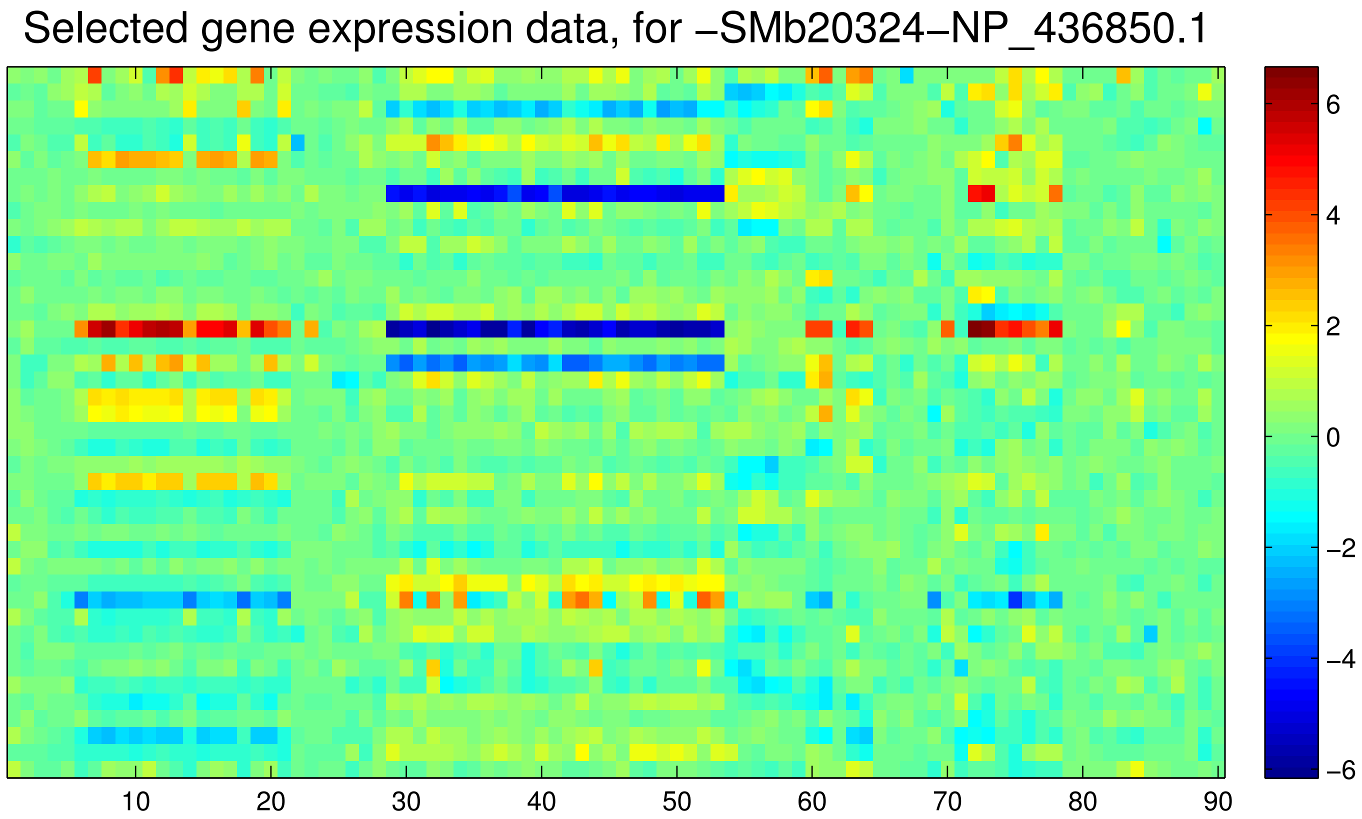
Selected gene expression data, for −SMb20324−NP_436850.1
10
20
30
40
50
60
70
80
90
6
4
2
0
−2
−4
−6

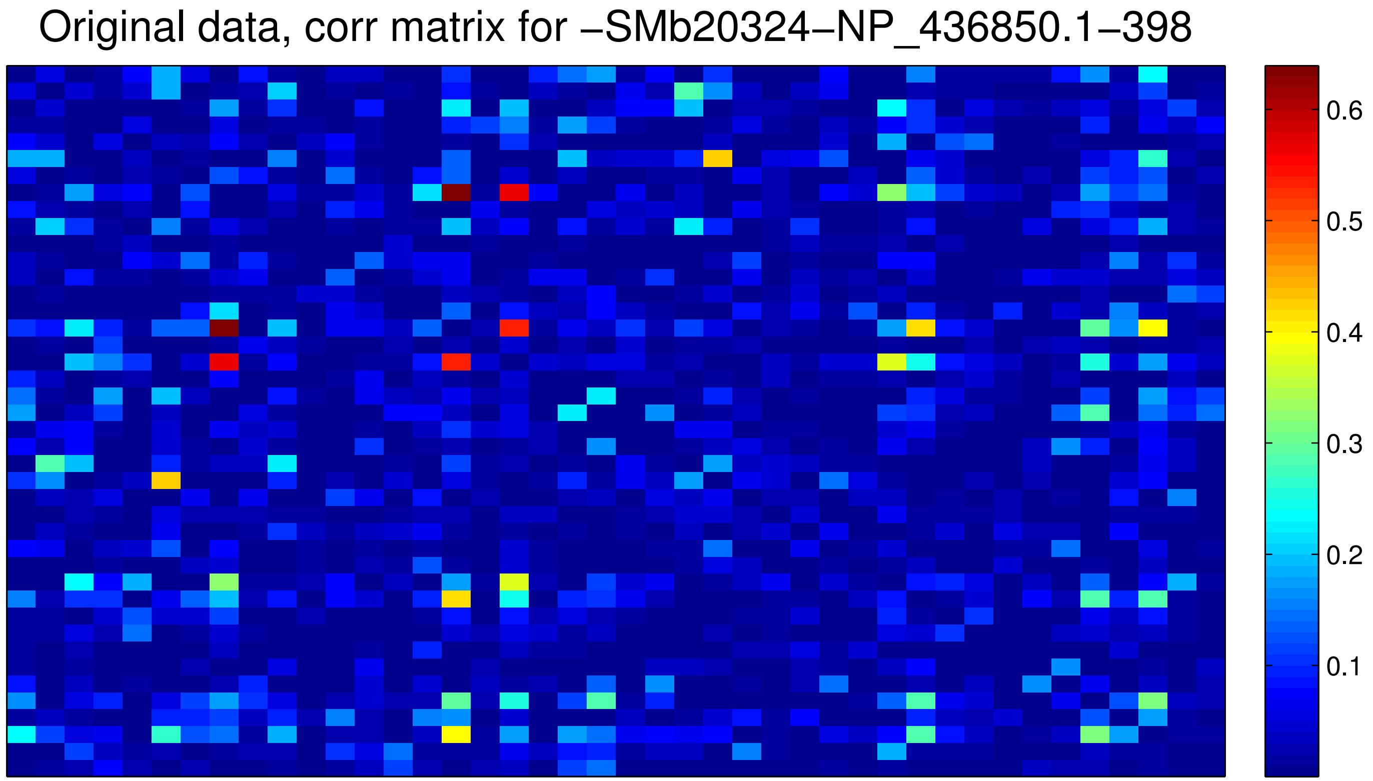
Original data, corr matrix for −SMb20324−NP_436850.1−398
0.6
0.5
0.4
0.3
0.2
0.1

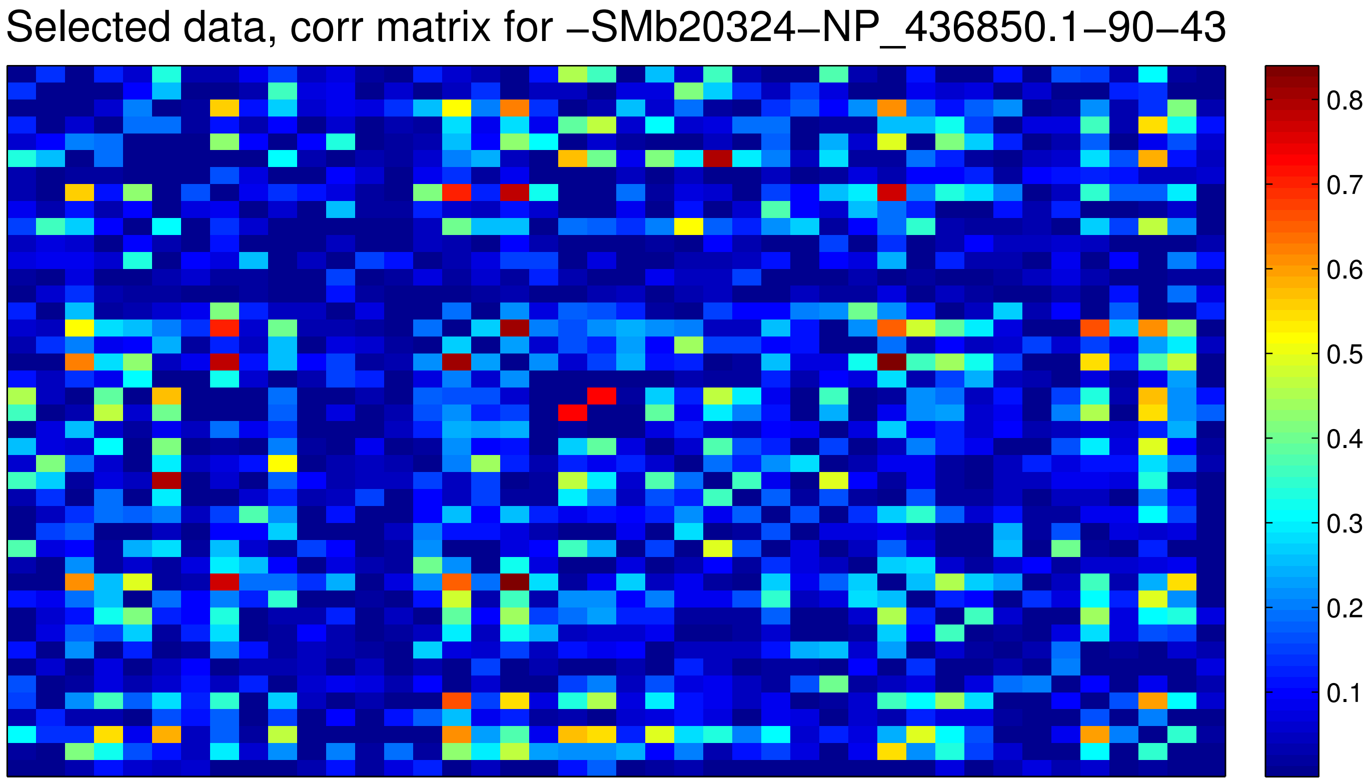
Selected data, corr matrix for −SMb20324−NP_436850.1−90−43
0.8
0.7
0.6
0.5
0.4
0.3
0.2
0.1
