# Supplementary material for: Evolution of Intra-specific Regulatory Networks in a Multipartite Bacterial Genome
Source: PLoS Comput Biol. 2015 Sep 4;11(9):e1004478. doi: 10.1371/journal.pcbi.1004478 (PMC4560400; doi:10.1371/journal.pcbi.1004478)
Supplement: S2 Material — (ZIP) [file pcbi.1004478.s002.zip › predictions/SMb20290.pdf]

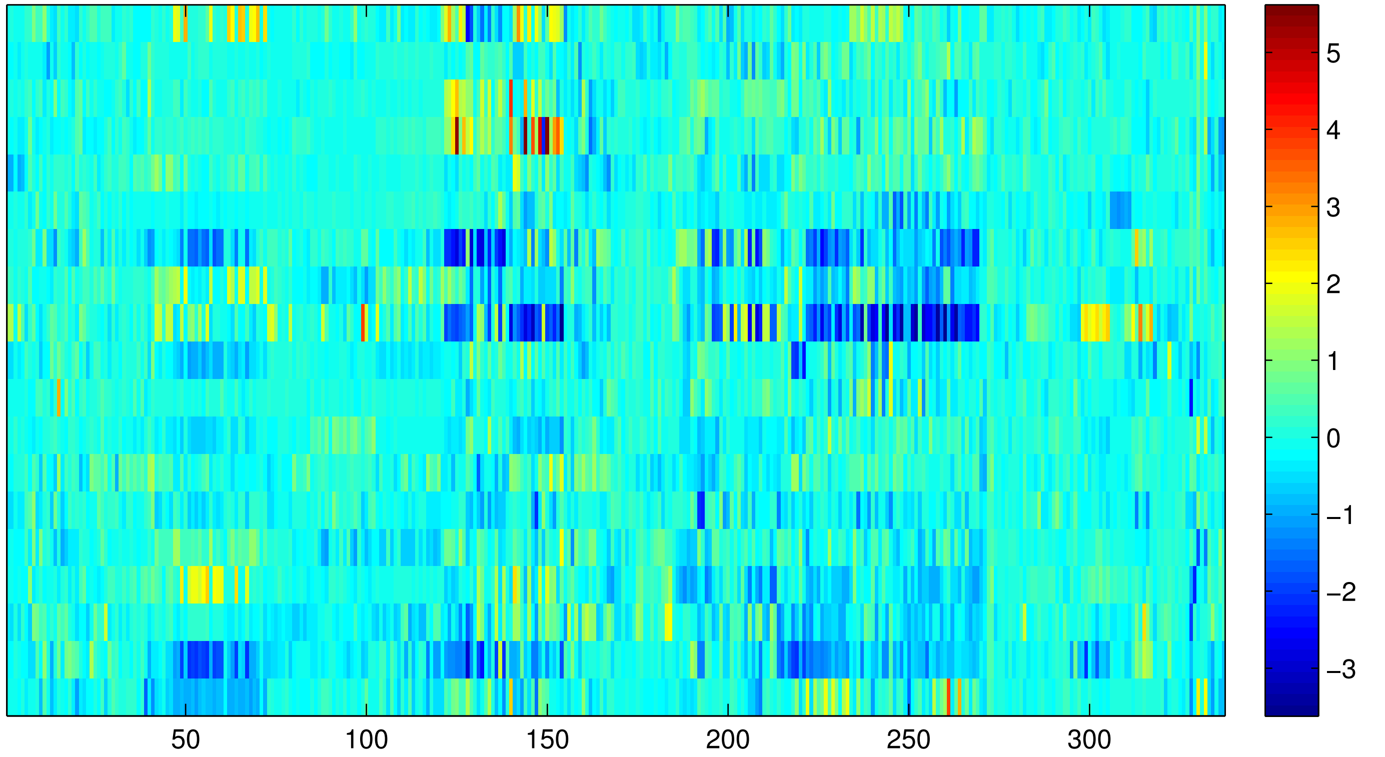


Original gene expression data, for −SMb20290−NP_436820.1

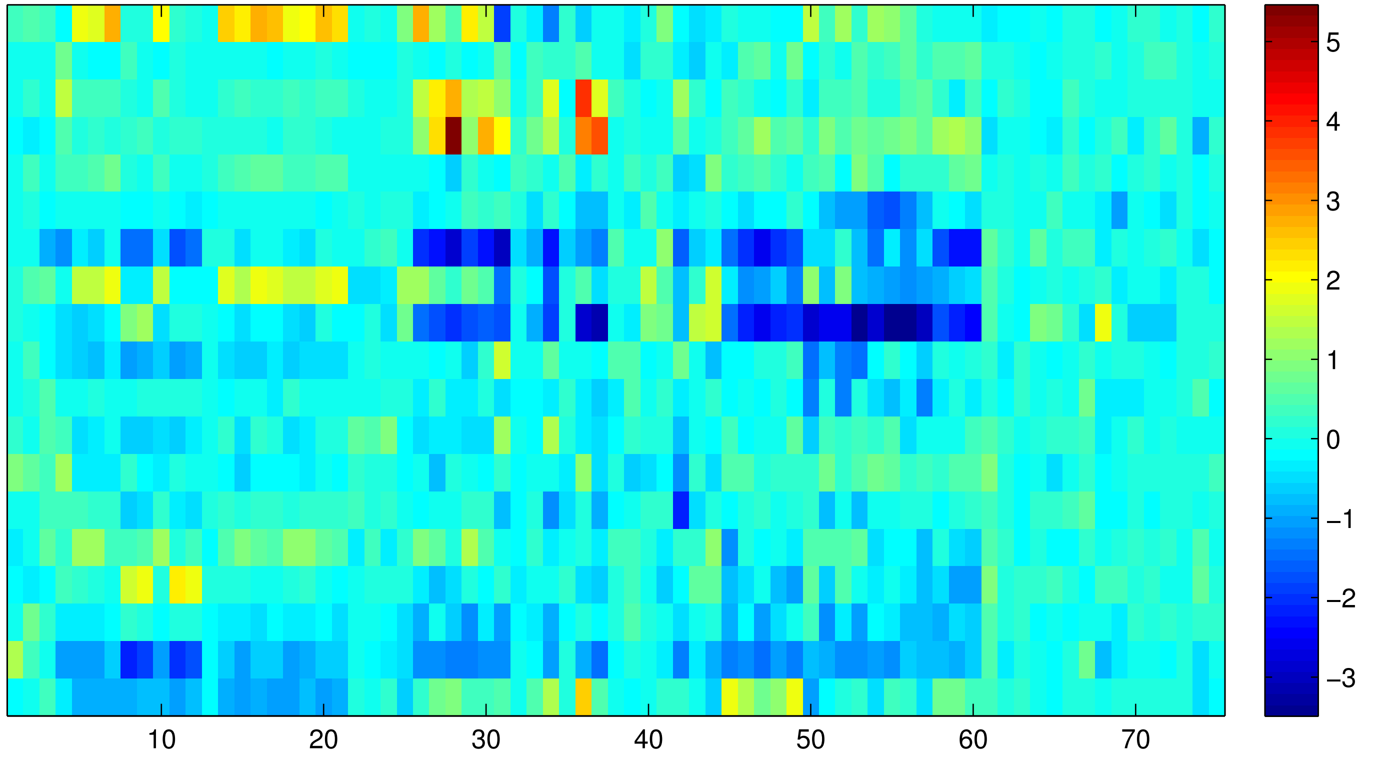


Selected gene expression data, for −SMb20290−NP_436820.1

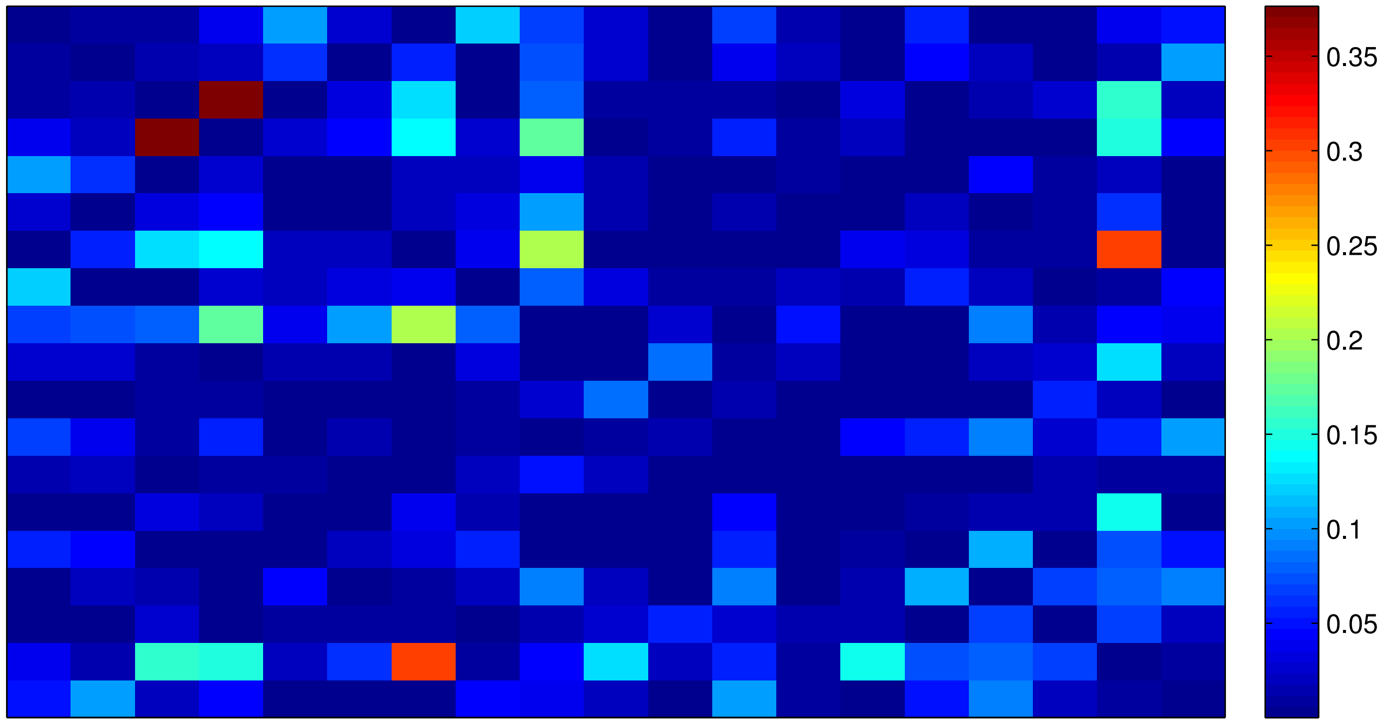


Original data, corr matrix for −SMb20290−NP_436820.1−337

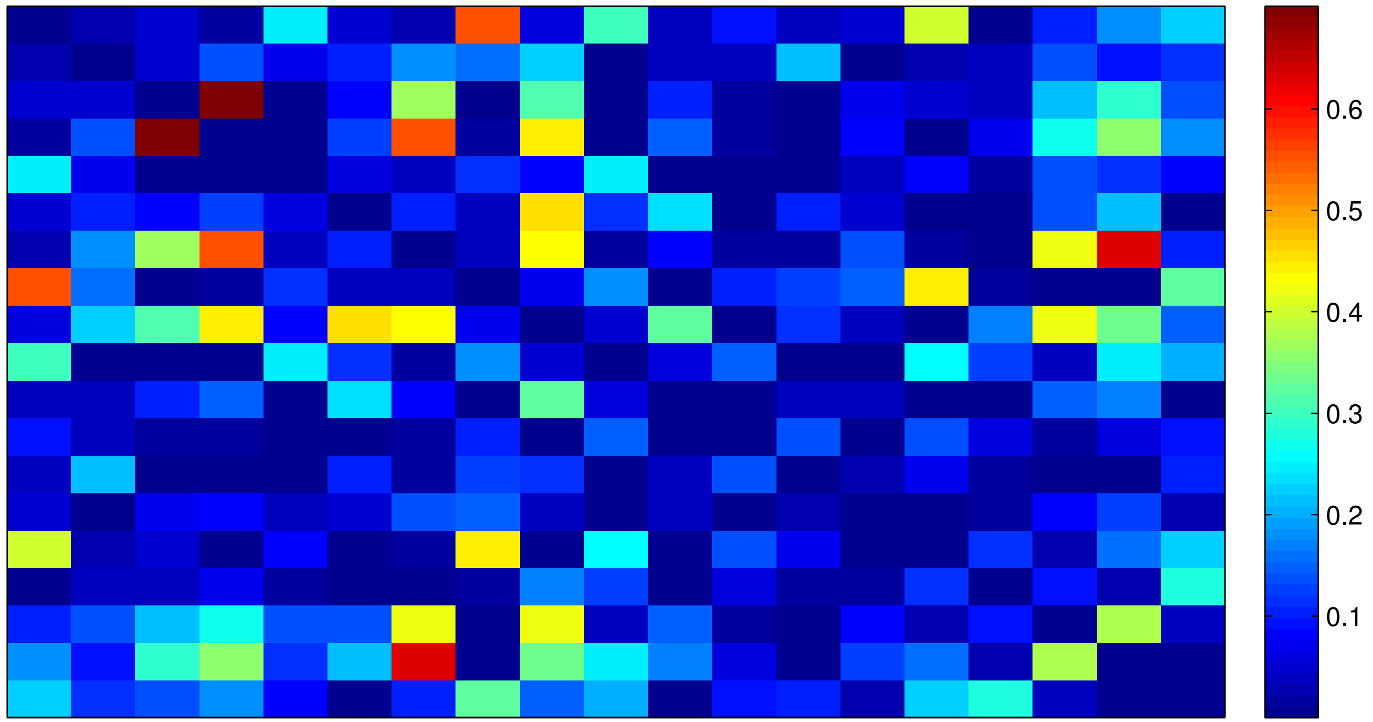


Selected data, corr matrix for −SMb20290−NP_436820.1−75−19
