# Supplementary material for: Evolution of Intra-specific Regulatory Networks in a Multipartite Bacterial Genome
Source: PLoS Comput Biol. 2015 Sep 4;11(9):e1004478. doi: 10.1371/journal.pcbi.1004478 (PMC4560400; doi:10.1371/journal.pcbi.1004478)
Supplement: S2 Material — (ZIP) [file pcbi.1004478.s002.zip › predictions/SMb20030.pdf]

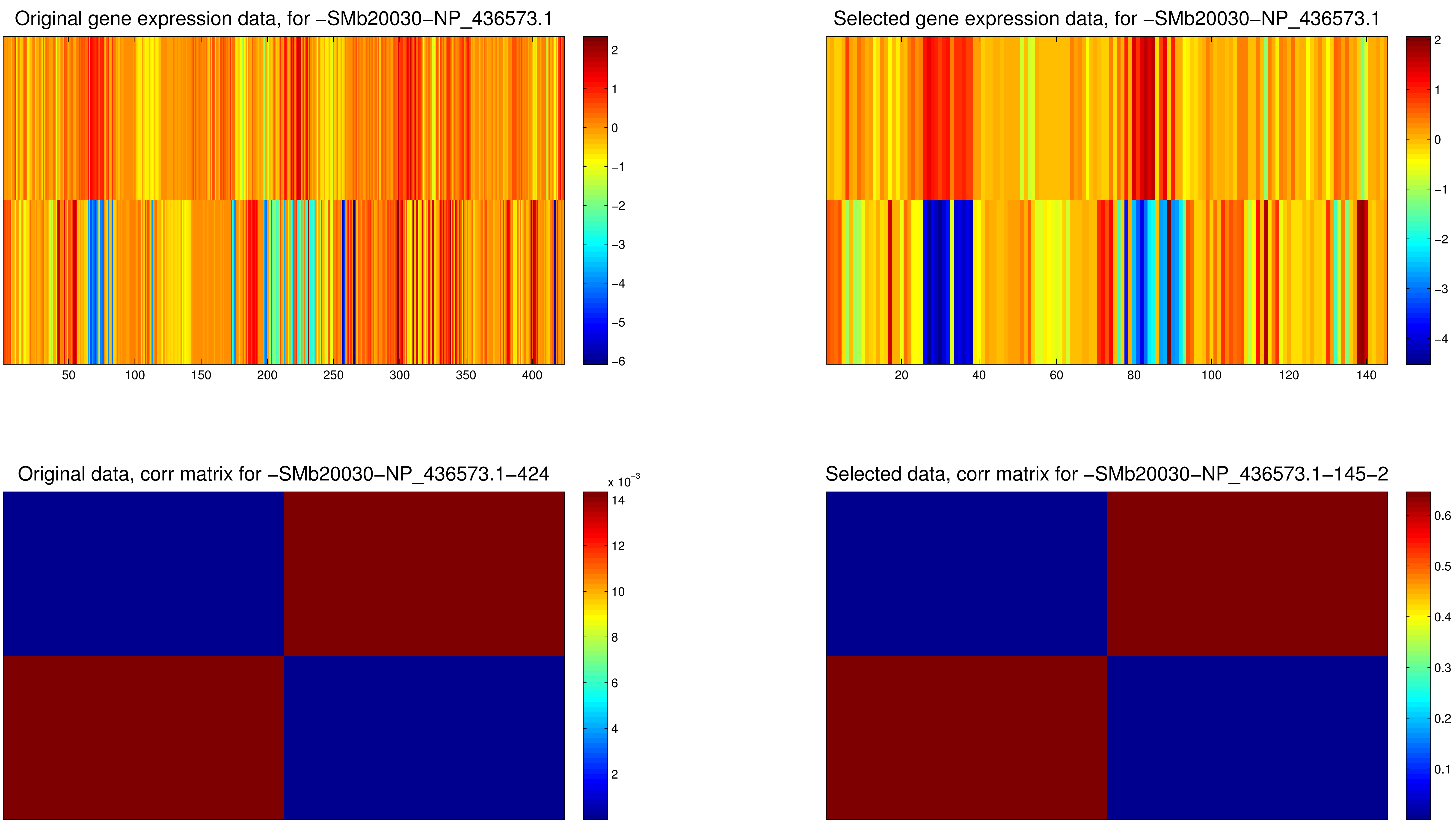
Original gene expression data, for −SMb20030−NP_436573.1
50 100 150 200 250 300 350 400
2 1 0 −1 −2 −3 −4 −5 −6
Selected gene expression data, for −SMb20030−NP_436573.1
20 40 60 80 100 120 140
2 1 0 −1 −2 −3 −4
Original data, corr matrix for −SMb20030−NP_436573.1−424
x 10−3
14 12 10 8 6 4 2
Selected data, corr matrix for −SMb20030−NP_436573.1−145−2
0.6 0.5 0.4 0.3 0.2 0.1
