# Supplementary material for: Evolution of Intra-specific Regulatory Networks in a Multipartite Bacterial Genome
Source: PLoS Comput Biol. 2015 Sep 4;11(9):e1004478. doi: 10.1371/journal.pcbi.1004478 (PMC4560400; doi:10.1371/journal.pcbi.1004478)
Supplement: S2 Material — (ZIP) [file pcbi.1004478.s002.zip › predictions/SMa2412.pdf]

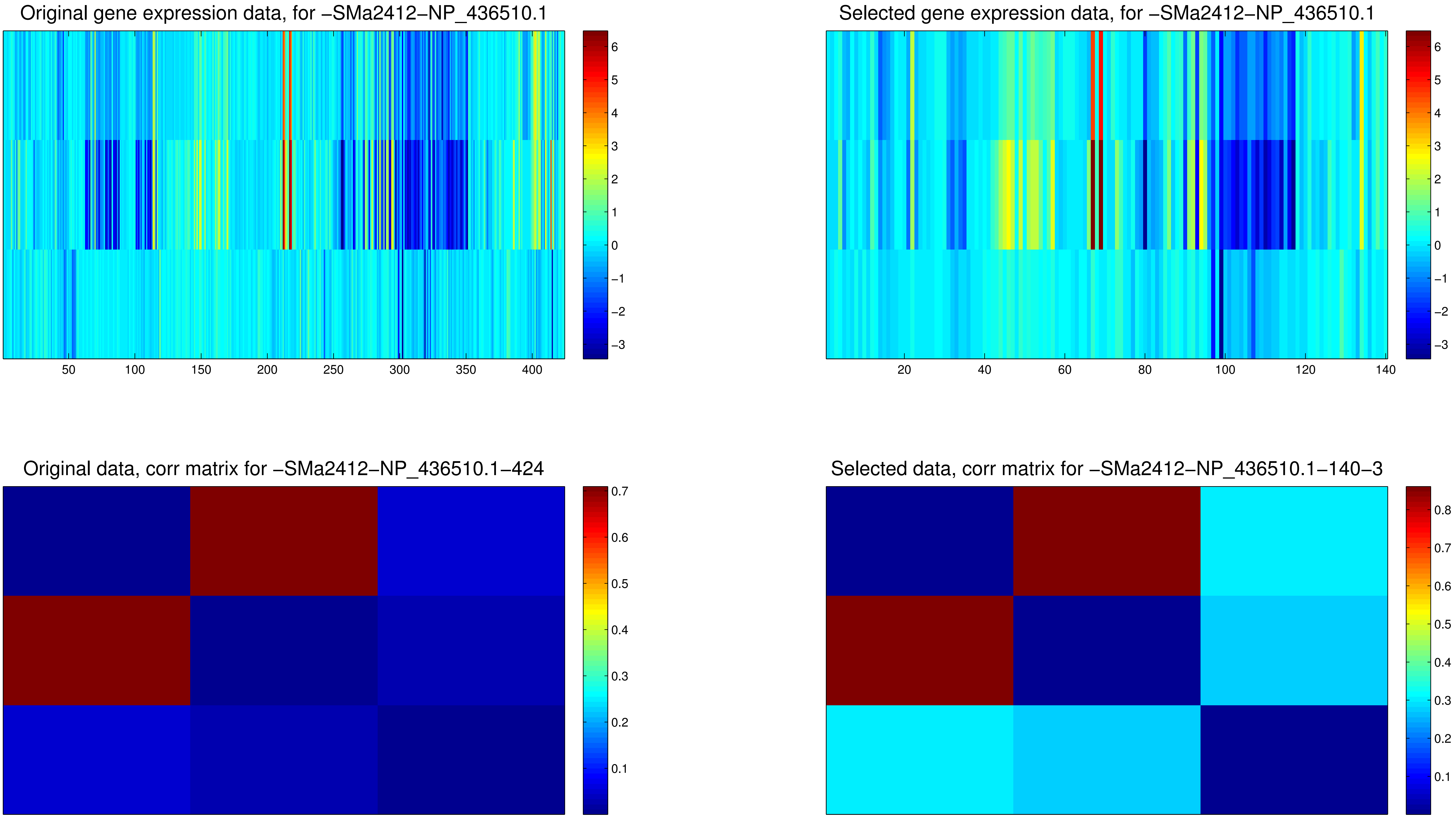

Original gene expression data, for –SMa2412–NP_436510.1
Selected gene expression data, for –SMa2412–NP_436510.1
Original data, corr matrix for –SMa2412–NP_436510.1–424
Selected data, corr matrix for –SMa2412–NP_436510.1–140–3
50
100
150
200
250
300
350
400
20
40
60
80
100
120
140
6
5
4
3
2
1
0
–1
–2
–3
0.7
0.6
0.5
0.4
0.3
0.2
0.1
0.8
