# Supplementary material for: Evolution of Intra-specific Regulatory Networks in a Multipartite Bacterial Genome
Source: PLoS Comput Biol. 2015 Sep 4;11(9):e1004478. doi: 10.1371/journal.pcbi.1004478 (PMC4560400; doi:10.1371/journal.pcbi.1004478)
Supplement: S2 Material — (ZIP) [file pcbi.1004478.s002.zip › predictions/SMa1225.pdf]

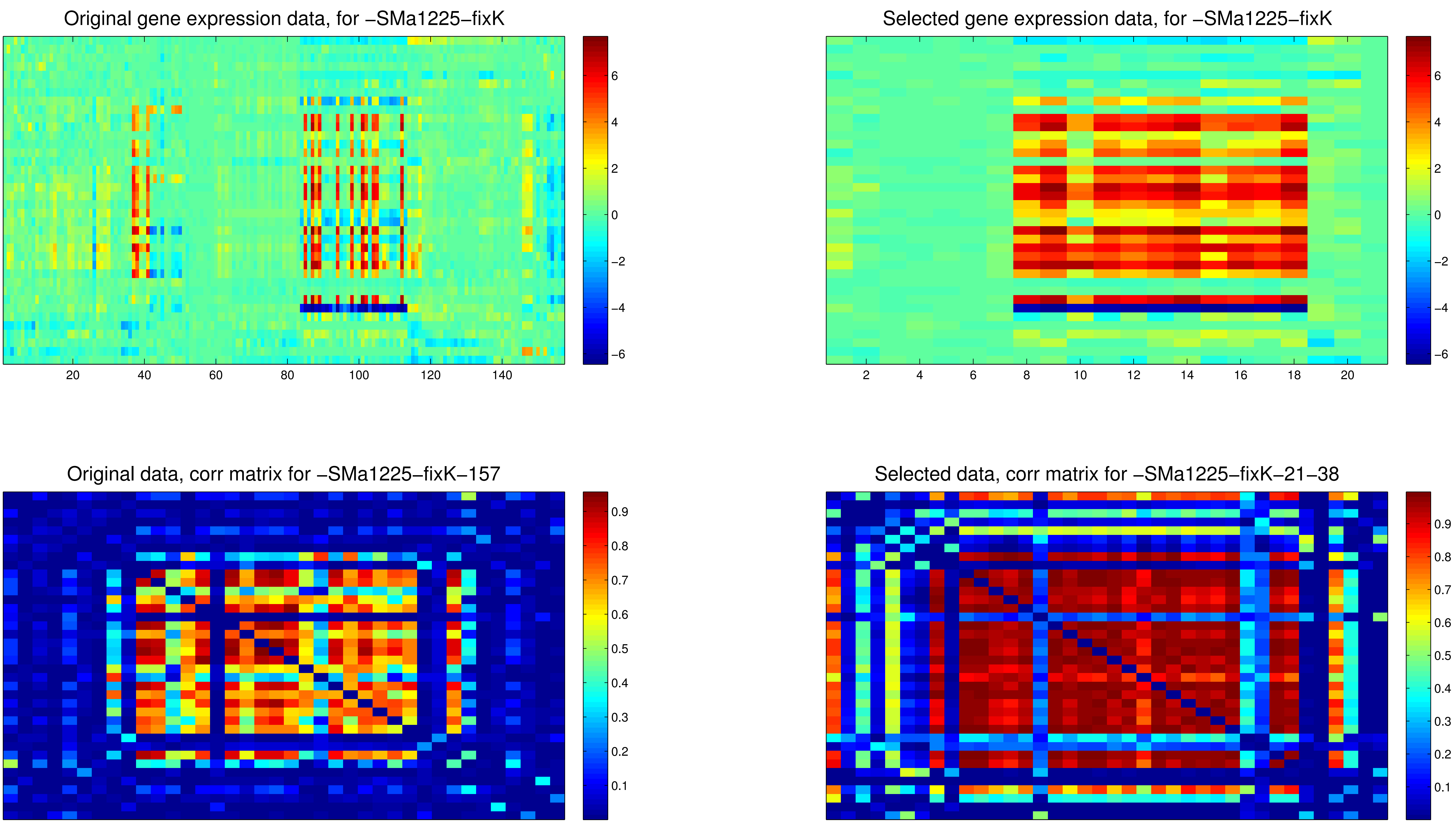

Original gene expression data, for −SMa1225−fixK
20
40
60
80
100
120
140
6
4
2
0
−2
−4
−6
Selected gene expression data, for −SMa1225−fixK
2
4
6
8
10
12
14
16
18
20
6
4
2
0
−2
−4
−6
Original data, corr matrix for −SMa1225−fixK−157
0.9
0.8
0.7
0.6
0.5
0.4
0.3
0.2
0.1
Selected data, corr matrix for −SMa1225−fixK−21−38
0.9
0.8
0.7
0.6
0.5
0.4
0.3
0.2
0.1
