# Supplementary material for: Evolution of Intra-specific Regulatory Networks in a Multipartite Bacterial Genome
Source: PLoS Comput Biol. 2015 Sep 4;11(9):e1004478. doi: 10.1371/journal.pcbi.1004478 (PMC4560400; doi:10.1371/journal.pcbi.1004478)
Supplement: S2 Material — (ZIP) [file pcbi.1004478.s002.zip › predictions/SMa0955.pdf]

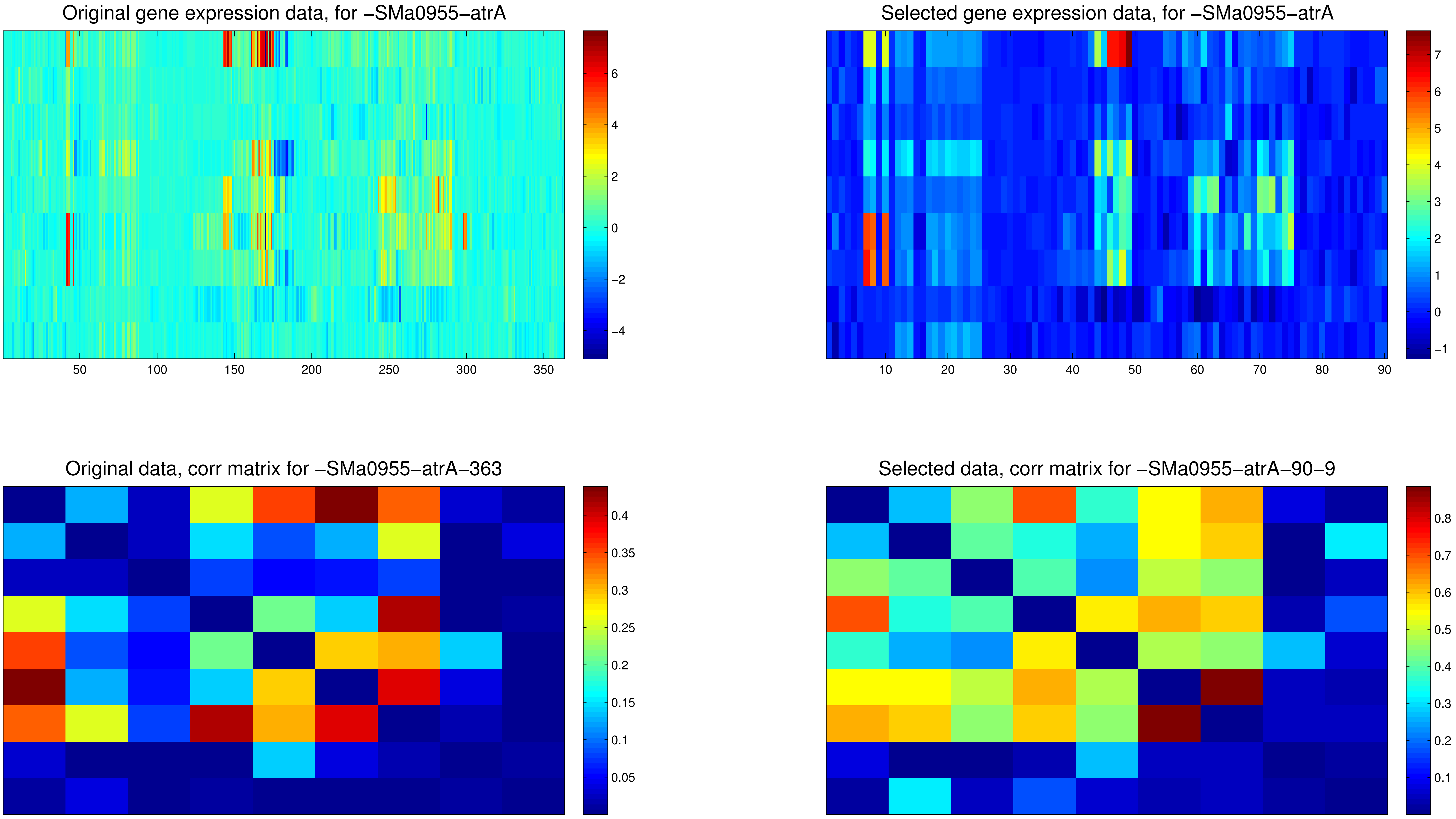
Original gene expression data, for –SMa0955–atrA
50
100
150
200
250
300
350
6
4
2
0
−2
−4
Selected gene expression data, for –SMa0955–atrA
10
20
30
40
50
60
70
80
90
7
6
5
4
3
2
1
0
−1
Original data, corr matrix for –SMa0955–atrA–363
0.4
0.35
0.3
0.25
0.2
0.15
0.1
0.05
Selected data, corr matrix for –SMa0955–atrA–90–9
0.8
0.7
0.6
0.5
0.4
0.3
0.2
0.1
