# Supplementary material for: Evolution of Intra-specific Regulatory Networks in a Multipartite Bacterial Genome
Source: PLoS Comput Biol. 2015 Sep 4;11(9):e1004478. doi: 10.1371/journal.pcbi.1004478 (PMC4560400; doi:10.1371/journal.pcbi.1004478)
Supplement: S2 Material — (ZIP) [file pcbi.1004478.s002.zip › predictions/SMa0870.pdf]

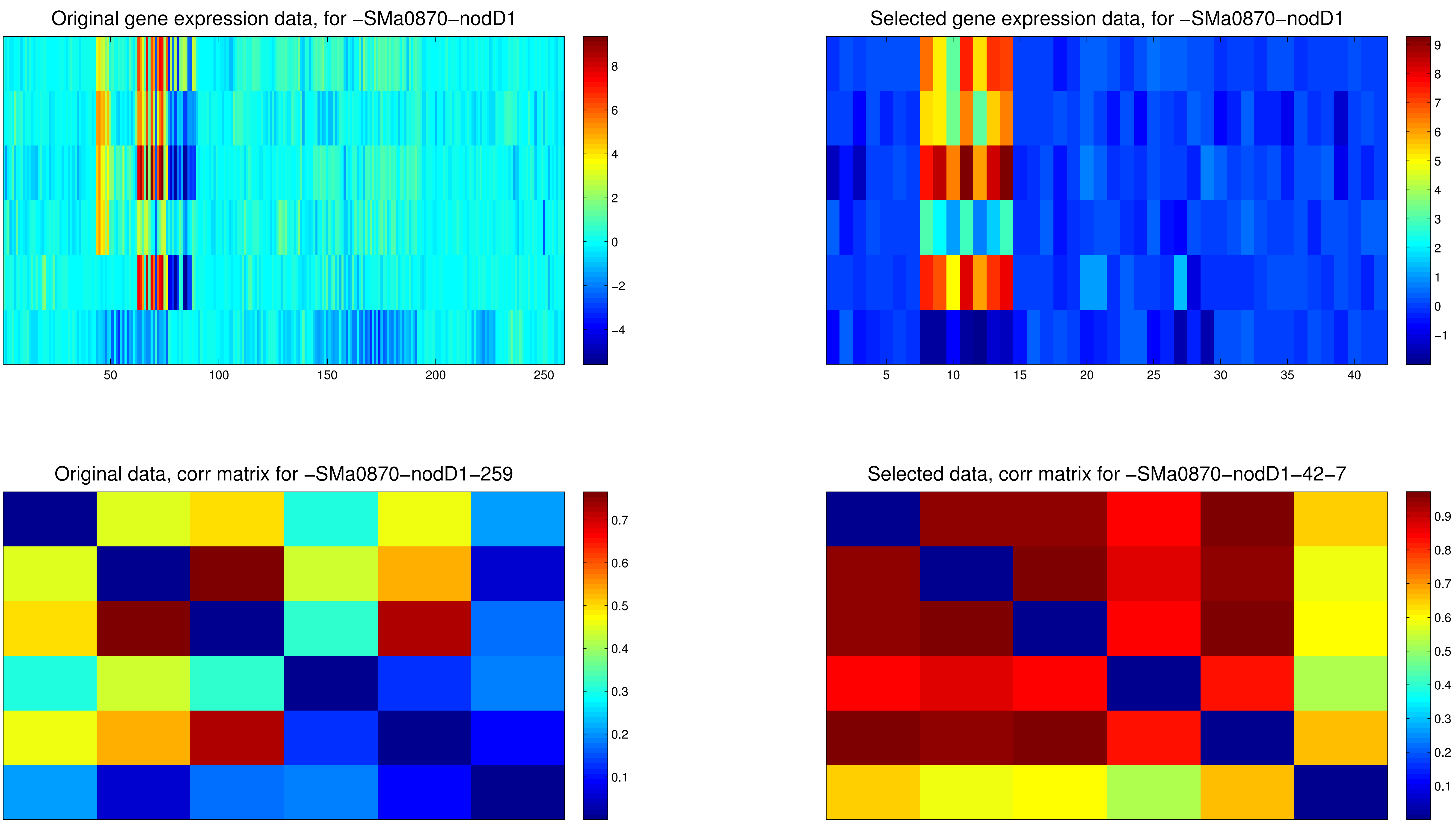
Original gene expression data, for −SMa0870−nodD1
50
100
150
200
250
8
6
4
2
0
−2
−4
Selected gene expression data, for −SMa0870−nodD1
5
10
15
20
25
30
35
40
9
8
7
6
5
4
3
2
1
0
−1
Original data, corr matrix for −SMa0870−nodD1−259
0.7
0.6
0.5
0.4
0.3
0.2
0.1
Selected data, corr matrix for −SMa0870−nodD1−42−7
0.9
0.8
0.7
0.6
0.5
0.4
0.3
0.2
0.1
