# Supplementary material for: Evolution of Intra-specific Regulatory Networks in a Multipartite Bacterial Genome
Source: PLoS Comput Biol. 2015 Sep 4;11(9):e1004478. doi: 10.1371/journal.pcbi.1004478 (PMC4560400; doi:10.1371/journal.pcbi.1004478)
Supplement: S2 Material — (ZIP) [file pcbi.1004478.s002.zip › predictions/SMa0815.pdf]

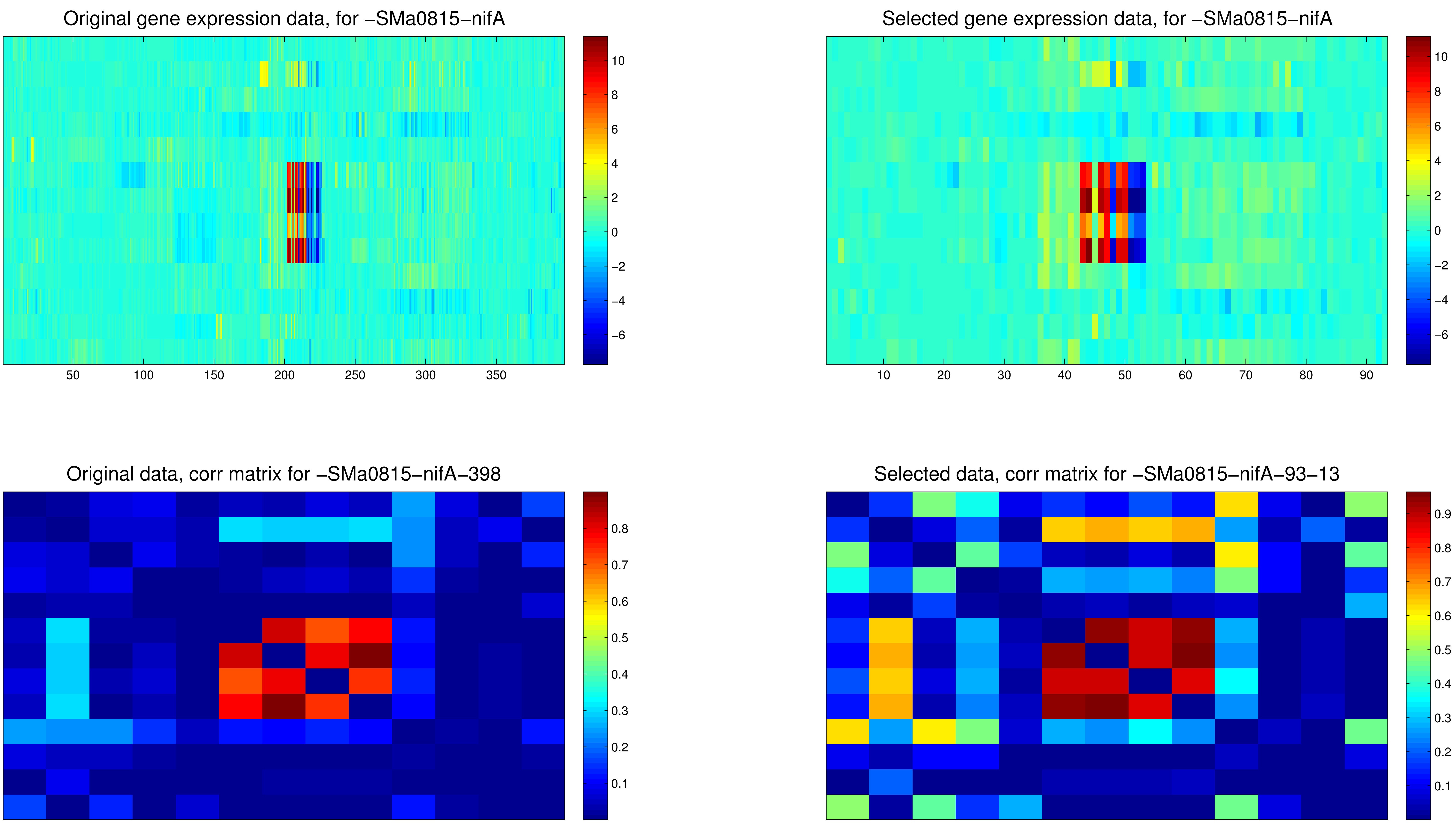
Original gene expression data, for –SMa0815–nifA
50
100
150
200
250
300
350
10
8
6
4
2
0
−2
−4
−6
Selected gene expression data, for –SMa0815–nifA
10
20
30
40
50
60
70
80
90
10
8
6
4
2
0
−2
−4
−6
Original data, corr matrix for –SMa0815–nifA–398
0.8
0.7
0.6
0.5
0.4
0.3
0.2
0.1
Selected data, corr matrix for –SMa0815–nifA–93–13
0.9
0.8
0.7
0.6
0.5
0.4
0.3
0.2
0.1
