# Supplementary material for: Evolution of Intra-specific Regulatory Networks in a Multipartite Bacterial Genome
Source: PLoS Comput Biol. 2015 Sep 4;11(9):e1004478. doi: 10.1371/journal.pcbi.1004478 (PMC4560400; doi:10.1371/journal.pcbi.1004478)
Supplement: S2 Material — (ZIP) [file pcbi.1004478.s002.zip › predictions/SMa0762.pdf]

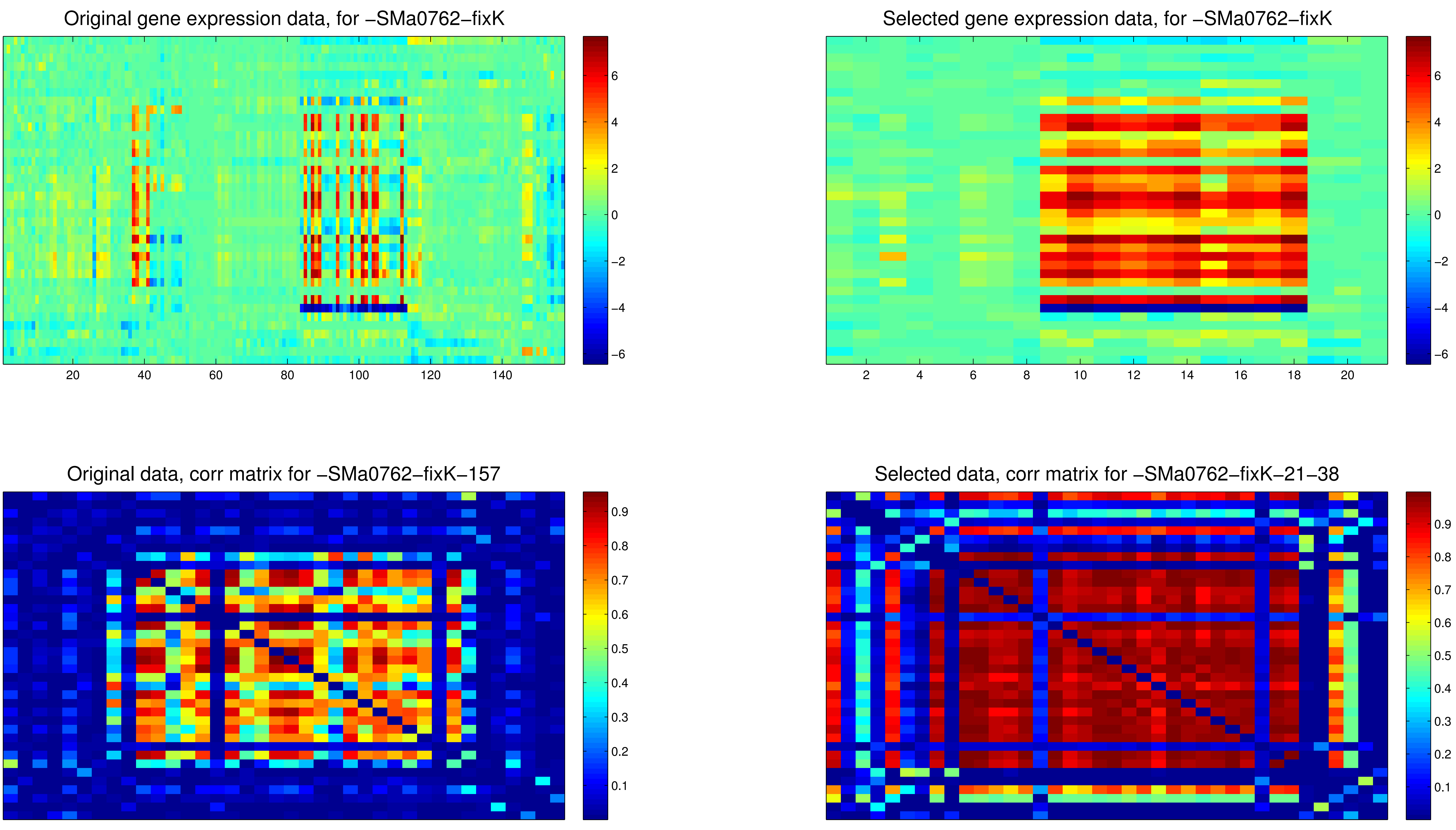

Original gene expression data, for –SMa0762–fixK
20 40 60 80 100 120 140
6 4 2 0 −2 −4 −6
Selected gene expression data, for –SMa0762–fixK
2 4 6 8 10 12 14 16 18 20
6 4 2 0 −2 −4 −6
Original data, corr matrix for –SMa0762–fixK–157
0.9 0.8 0.7 0.6 0.5 0.4 0.3 0.2 0.1
Selected data, corr matrix for –SMa0762–fixK–21–38
0.9 0.8 0.7 0.6 0.5 0.4 0.3 0.2 0.1
