# Supplementary material for: Evolution of Intra-specific Regulatory Networks in a Multipartite Bacterial Genome
Source: PLoS Comput Biol. 2015 Sep 4;11(9):e1004478. doi: 10.1371/journal.pcbi.1004478 (PMC4560400; doi:10.1371/journal.pcbi.1004478)
Supplement: S2 Material — (ZIP) [file pcbi.1004478.s002.zip › predictions_20_or_more/SMc04401.pdf]

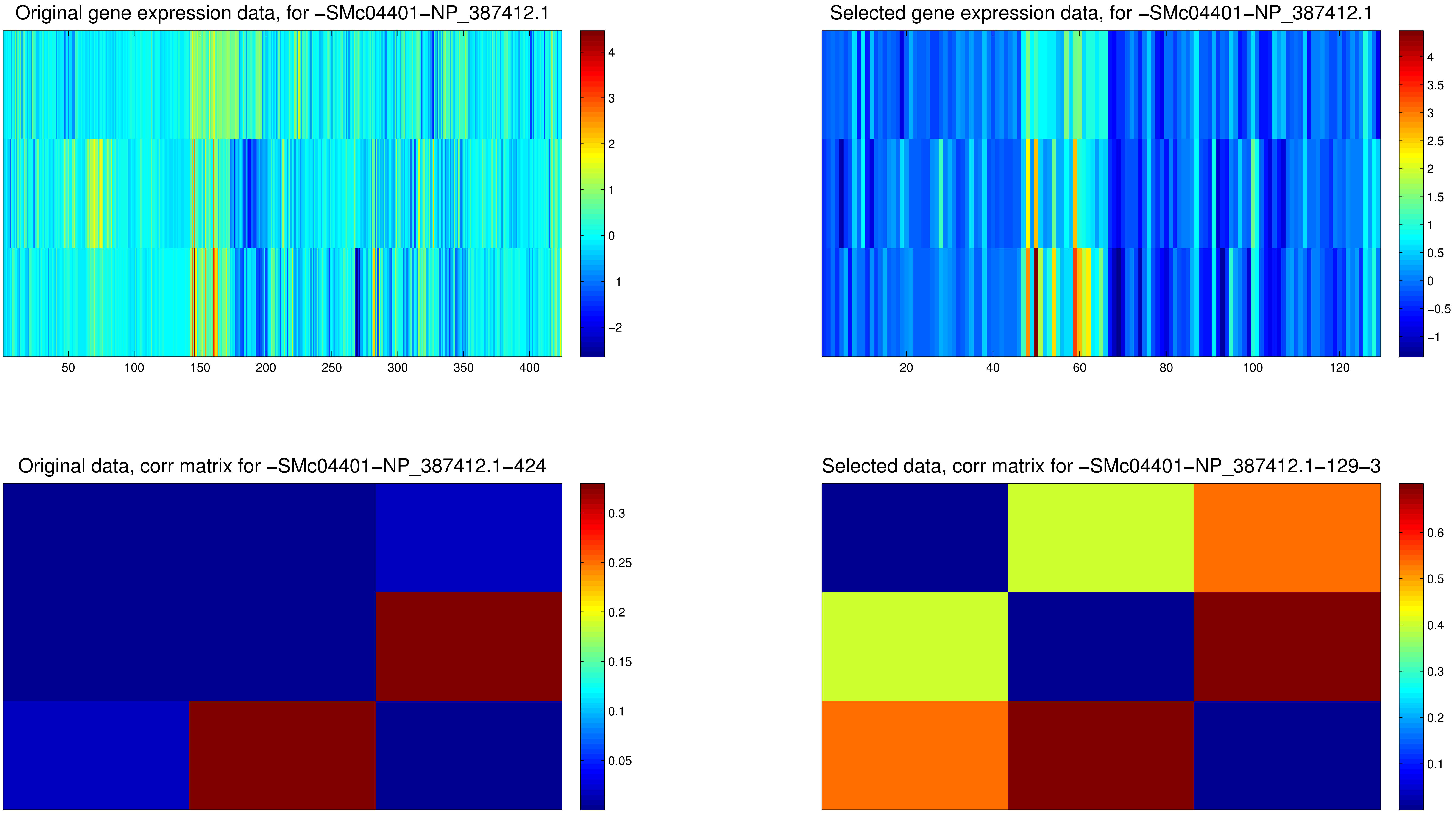
Original gene expression data, for –SMc04401–NP_387412.1
50 100 150 200 250 300 350 400
4 3 2 1 0 −1 −2
Selected gene expression data, for –SMc04401–NP_387412.1
20 40 60 80 100 120
4 3.5 3 2.5 2 1.5 1 0.5 0 −0.5 −1
Original data, corr matrix for –SMc04401–NP_387412.1–424
0.3 0.25 0.2 0.15 0.1 0.05
Selected data, corr matrix for –SMc04401–NP_387412.1–129–3
0.6 0.5 0.4 0.3 0.2 0.1
