# Supplementary material for: Evolution of Intra-specific Regulatory Networks in a Multipartite Bacterial Genome
Source: PLoS Comput Biol. 2015 Sep 4;11(9):e1004478. doi: 10.1371/journal.pcbi.1004478 (PMC4560400; doi:10.1371/journal.pcbi.1004478)
Supplement: S2 Material — (ZIP) [file pcbi.1004478.s002.zip › predictions_20_or_more/SMc02510.pdf]

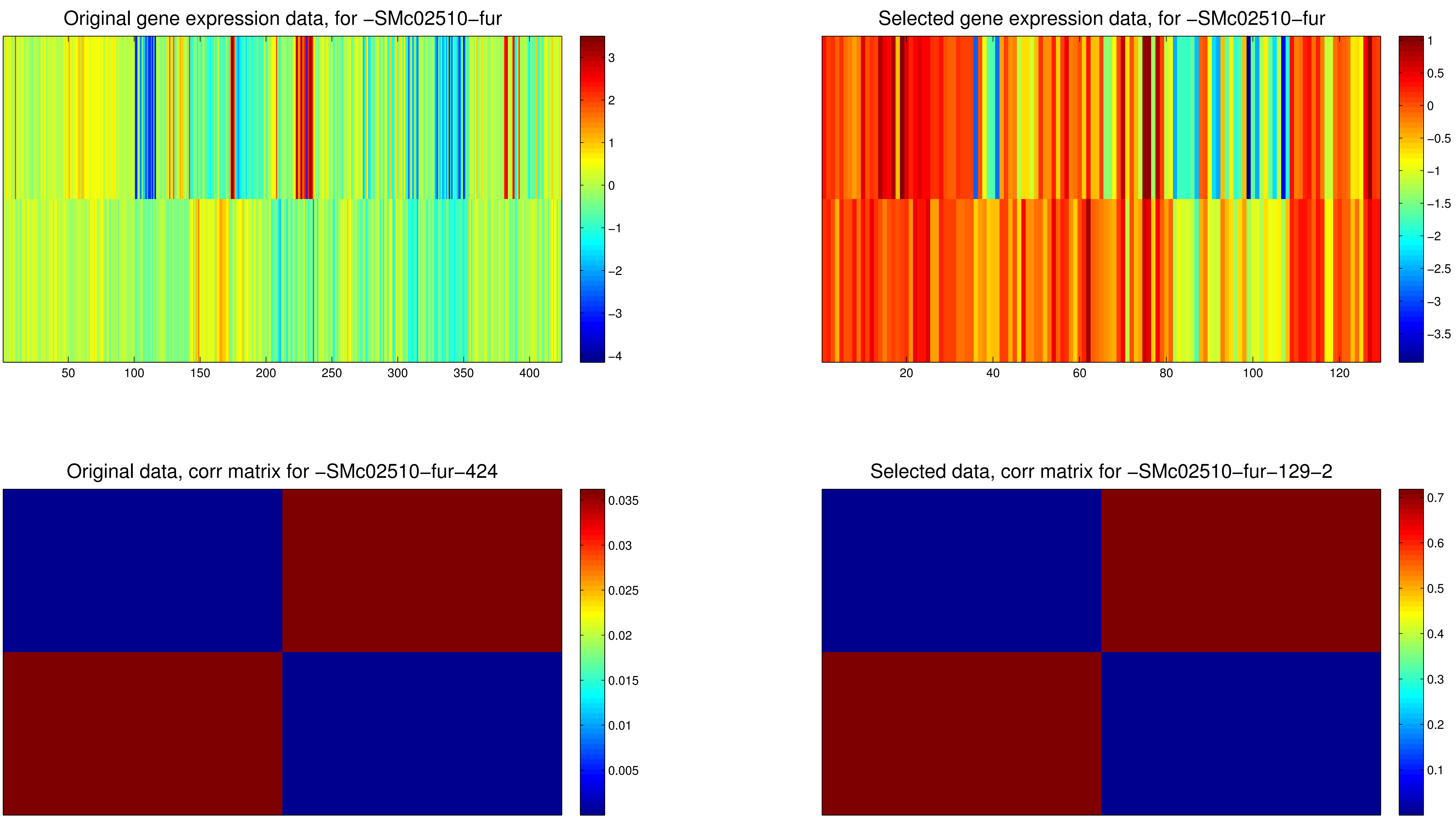
Original gene expression data, for −SMc02510−fur
50 100 150 200 250 300 350 400
3 2 1 0 −1 −2 −3 −4
Selected gene expression data, for −SMc02510−fur
20 40 60 80 100 120
1 0.5 0 −0.5 −1 −1.5 −2 −2.5 −3 −3.5
Original data, corr matrix for −SMc02510−fur−424
0.035 0.03 0.025 0.02 0.015 0.01 0.005
Selected data, corr matrix for −SMc02510−fur−129−2
0.7 0.6 0.5 0.4 0.3 0.2 0.1
