# Supplementary material for: Evolution of Intra-specific Regulatory Networks in a Multipartite Bacterial Genome
Source: PLoS Comput Biol. 2015 Sep 4;11(9):e1004478. doi: 10.1371/journal.pcbi.1004478 (PMC4560400; doi:10.1371/journal.pcbi.1004478)
Supplement: S2 Material — (ZIP) [file pcbi.1004478.s002.zip › predictions_20_or_more/SMc00095.pdf]

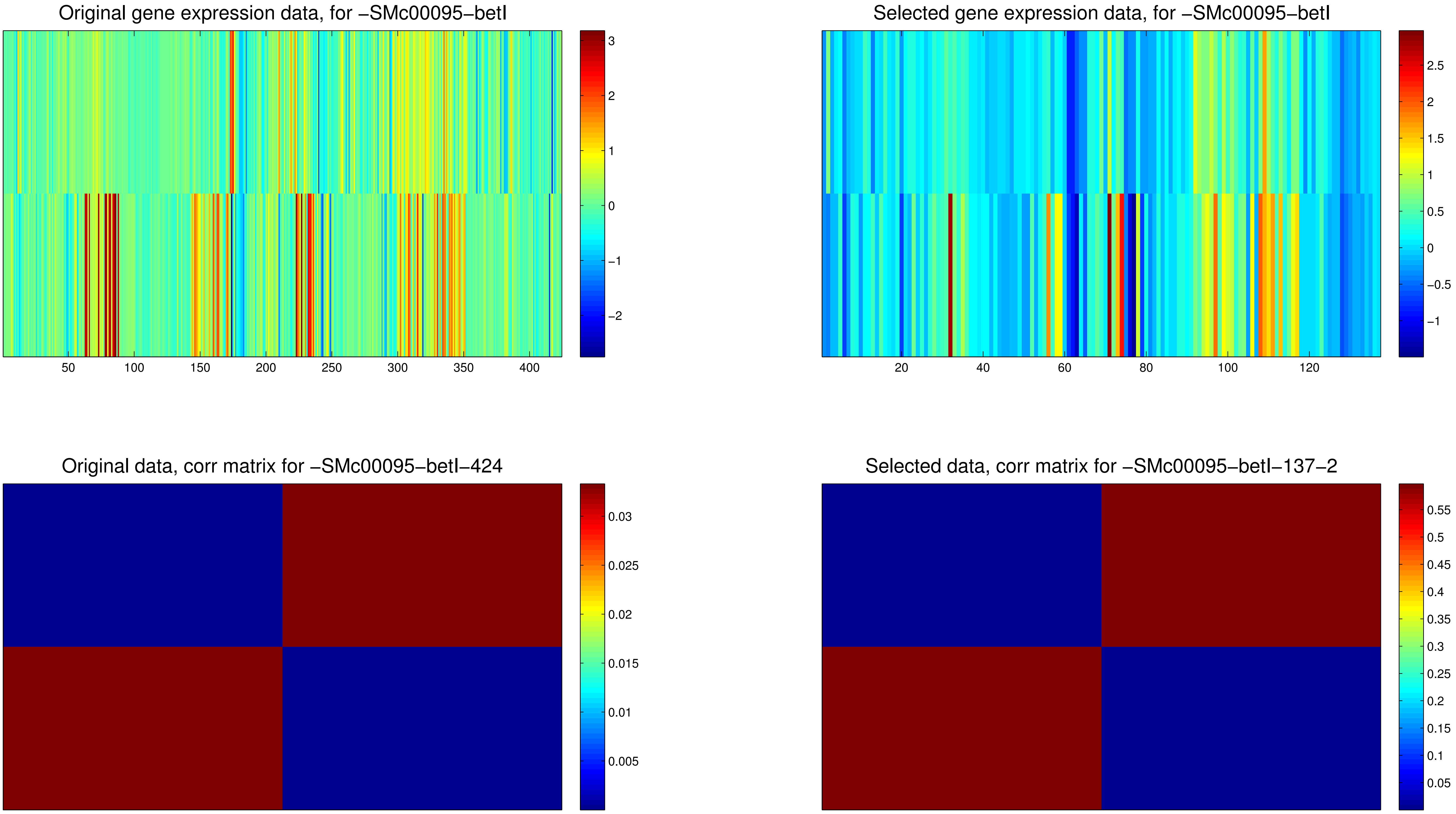
Original gene expression data, for −SMc00095−betI
50
100
150
200
250
300
350
400
3
2
1
0
−1
−2
Selected gene expression data, for −SMc00095−betI
20
40
60
80
100
120
2.5
2
1.5
1
0.5
0
−0.5
−1
Original data, corr matrix for −SMc00095−betI−424
0.03
0.025
0.02
0.015
0.01
0.005
Selected data, corr matrix for −SMc00095−betI−137−2
0.55
0.5
0.45
0.4
0.35
0.3
0.25
0.2
0.15
0.1
0.05
