# Supplementary material for: Evolution of Intra-specific Regulatory Networks in a Multipartite Bacterial Genome
Source: PLoS Comput Biol. 2015 Sep 4;11(9):e1004478. doi: 10.1371/journal.pcbi.1004478 (PMC4560400; doi:10.1371/journal.pcbi.1004478)
Supplement: S2 Material — (ZIP) [file pcbi.1004478.s002.zip › predictions_20_or_more/SMc00058.pdf]

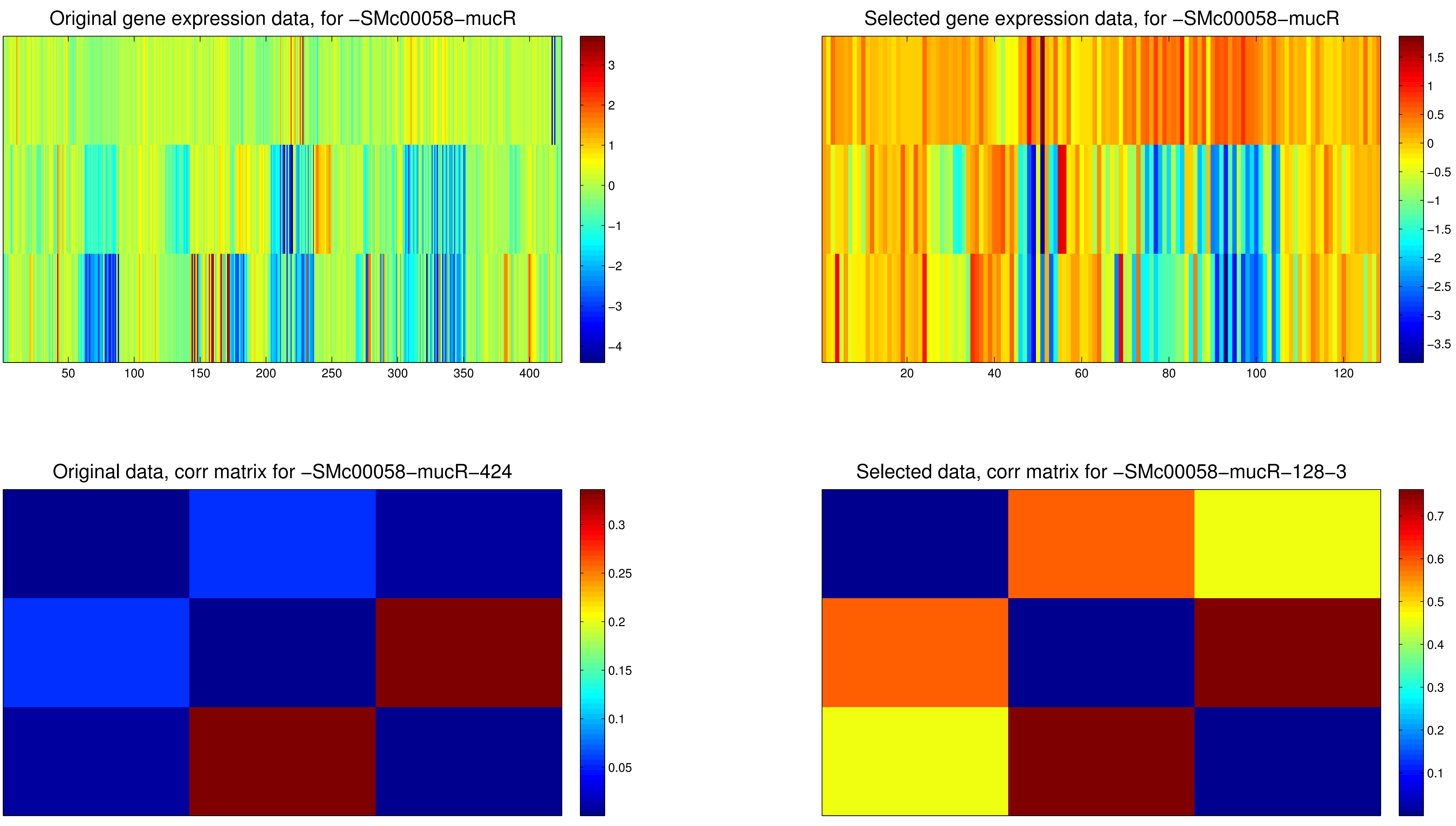

Original gene expression data, for –SMc00058–mucR
50 100 150 200 250 300 350 400
3 2 1 0 –1 –2 –3 –4
Selected gene expression data, for –SMc00058–mucR
20 40 60 80 100 120
1.5 1 0.5 0 –0.5 –1 –1.5 –2 –2.5 –3 –3.5
Original data, corr matrix for –SMc00058–mucR–424
0.3 0.25 0.2 0.15 0.1 0.05
Selected data, corr matrix for –SMc00058–mucR–128–3
0.7 0.6 0.5 0.4 0.3 0.2 0.1
