# Supplementary material for: Evolution of Intra-specific Regulatory Networks in a Multipartite Bacterial Genome
Source: PLoS Comput Biol. 2015 Sep 4;11(9):e1004478. doi: 10.1371/journal.pcbi.1004478 (PMC4560400; doi:10.1371/journal.pcbi.1004478)
Supplement: S2 Material — (ZIP) [file pcbi.1004478.s002.zip › predictions_20_or_more/SMa2412.pdf]

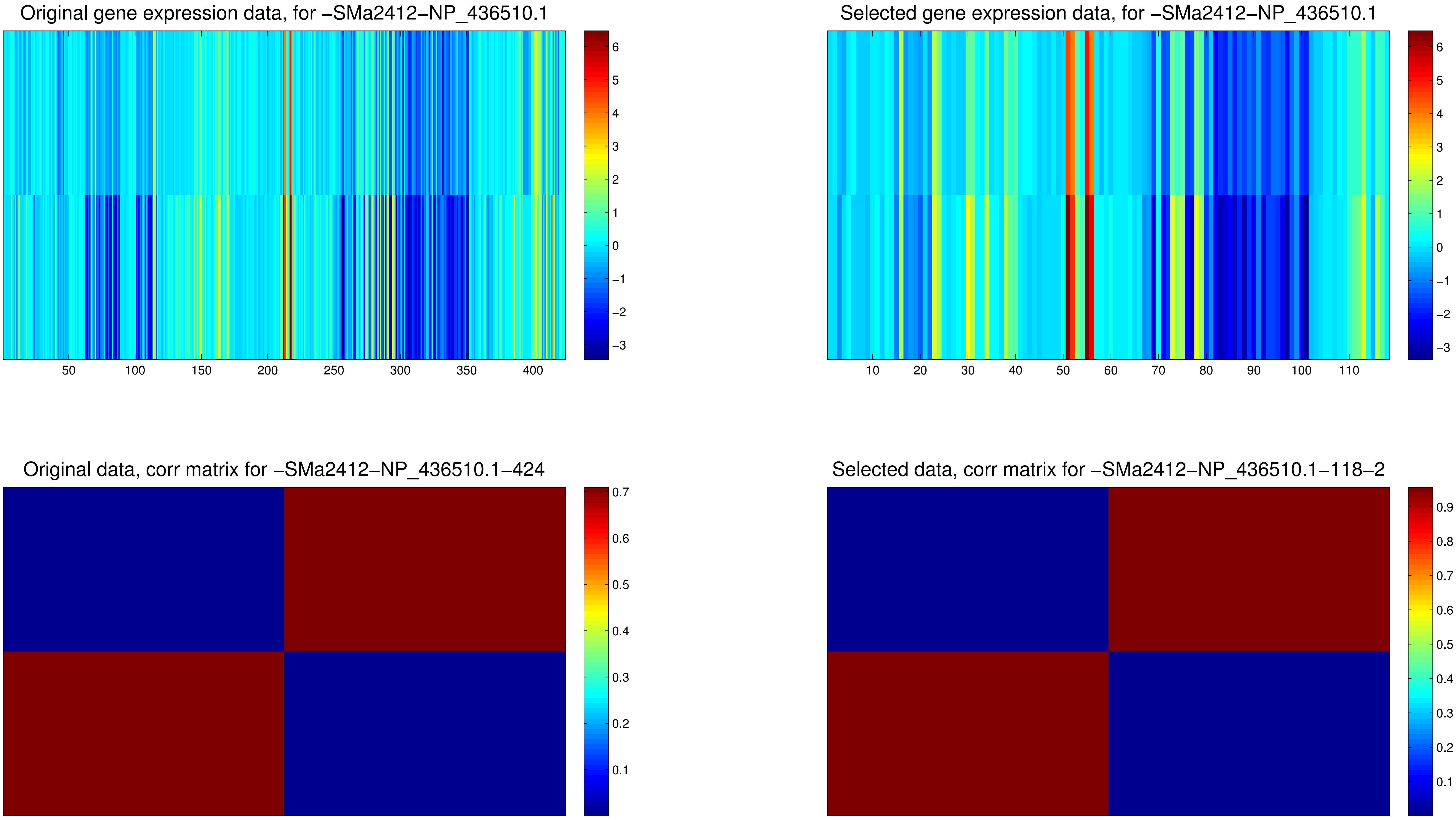
Original gene expression data, for –SMa2412–NP_436510.1
50 100 150 200 250 300 350 400
−3 −2 −1 0 1 2 3 4 5 6
Selected gene expression data, for –SMa2412–NP_436510.1
10 20 30 40 50 60 70 80 90 100 110
−3 −2 −1 0 1 2 3 4 5 6
Original data, corr matrix for –SMa2412–NP_436510.1–424
0.1 0.2 0.3 0.4 0.5 0.6 0.7
Selected data, corr matrix for –SMa2412–NP_436510.1–118–2
0.1 0.2 0.3 0.4 0.5 0.6 0.7 0.8 0.9
