# Supplementary material for: Evolution of Intra-specific Regulatory Networks in a Multipartite Bacterial Genome
Source: PLoS Comput Biol. 2015 Sep 4;11(9):e1004478. doi: 10.1371/journal.pcbi.1004478 (PMC4560400; doi:10.1371/journal.pcbi.1004478)
Supplement: S2 Material — (ZIP) [file pcbi.1004478.s002.zip › predictions_20_or_more/SMa0815.pdf]

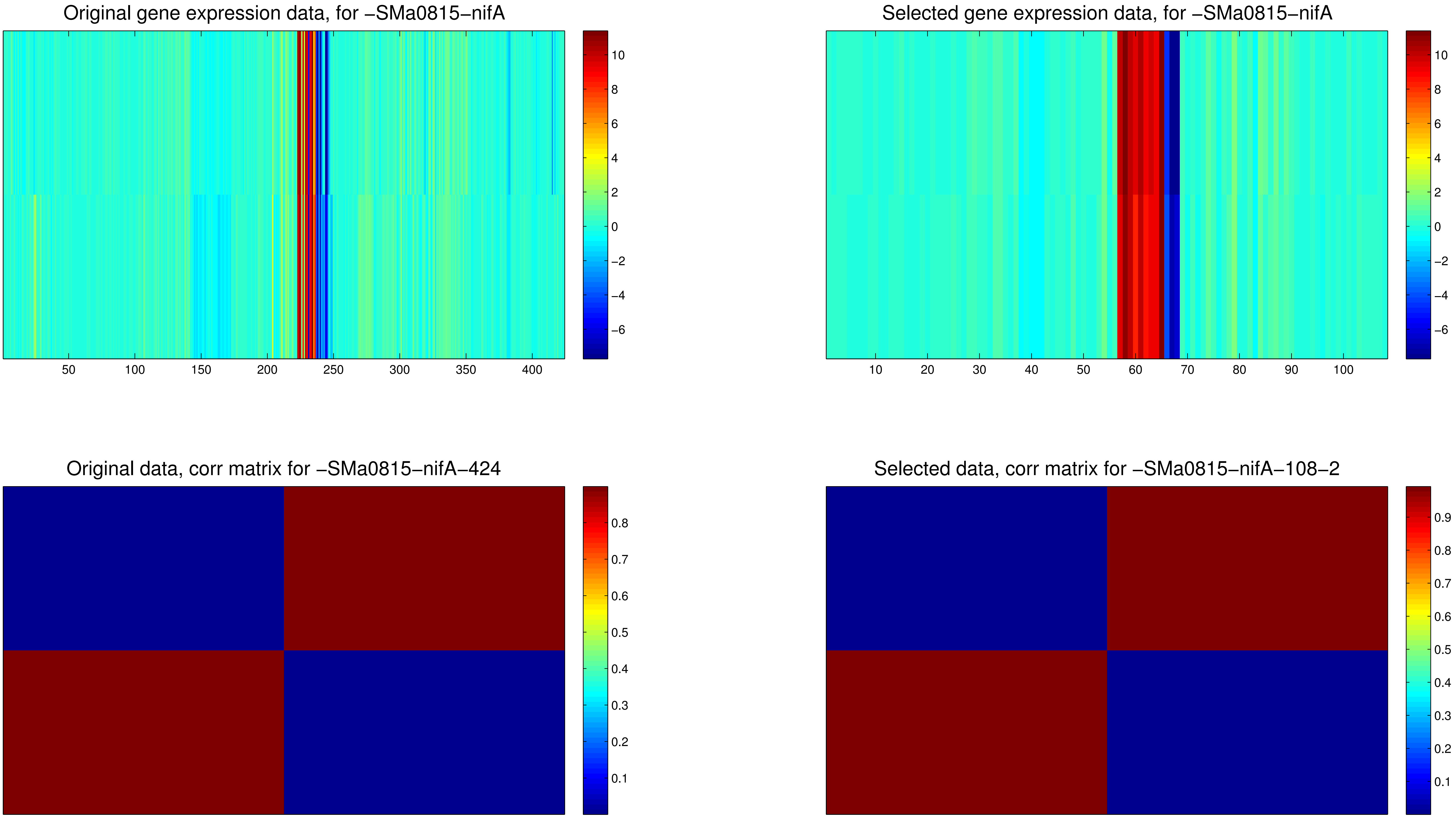
Original gene expression data, for −SMa0815−nifA
50 100 150 200 250 300 350 400
10 8 6 4 2 0 −2 −4 −6
Selected gene expression data, for −SMa0815−nifA
10 20 30 40 50 60 70 80 90 100
10 8 6 4 2 0 −2 −4 −6
Original data, corr matrix for −SMa0815−nifA−424
0.8 0.7 0.6 0.5 0.4 0.3 0.2 0.1
Selected data, corr matrix for −SMa0815−nifA−108−2
0.9 0.8 0.7 0.6 0.5 0.4 0.3 0.2 0.1
