# Supplementary material for: Evolution of Intra-specific Regulatory Networks in a Multipartite Bacterial Genome
Source: PLoS Comput Biol. 2015 Sep 4;11(9):e1004478. doi: 10.1371/journal.pcbi.1004478 (PMC4560400; doi:10.1371/journal.pcbi.1004478)
Supplement: S2 Material — (ZIP) [file pcbi.1004478.s002.zip › predictions_20_or_more/SMa0757.pdf]

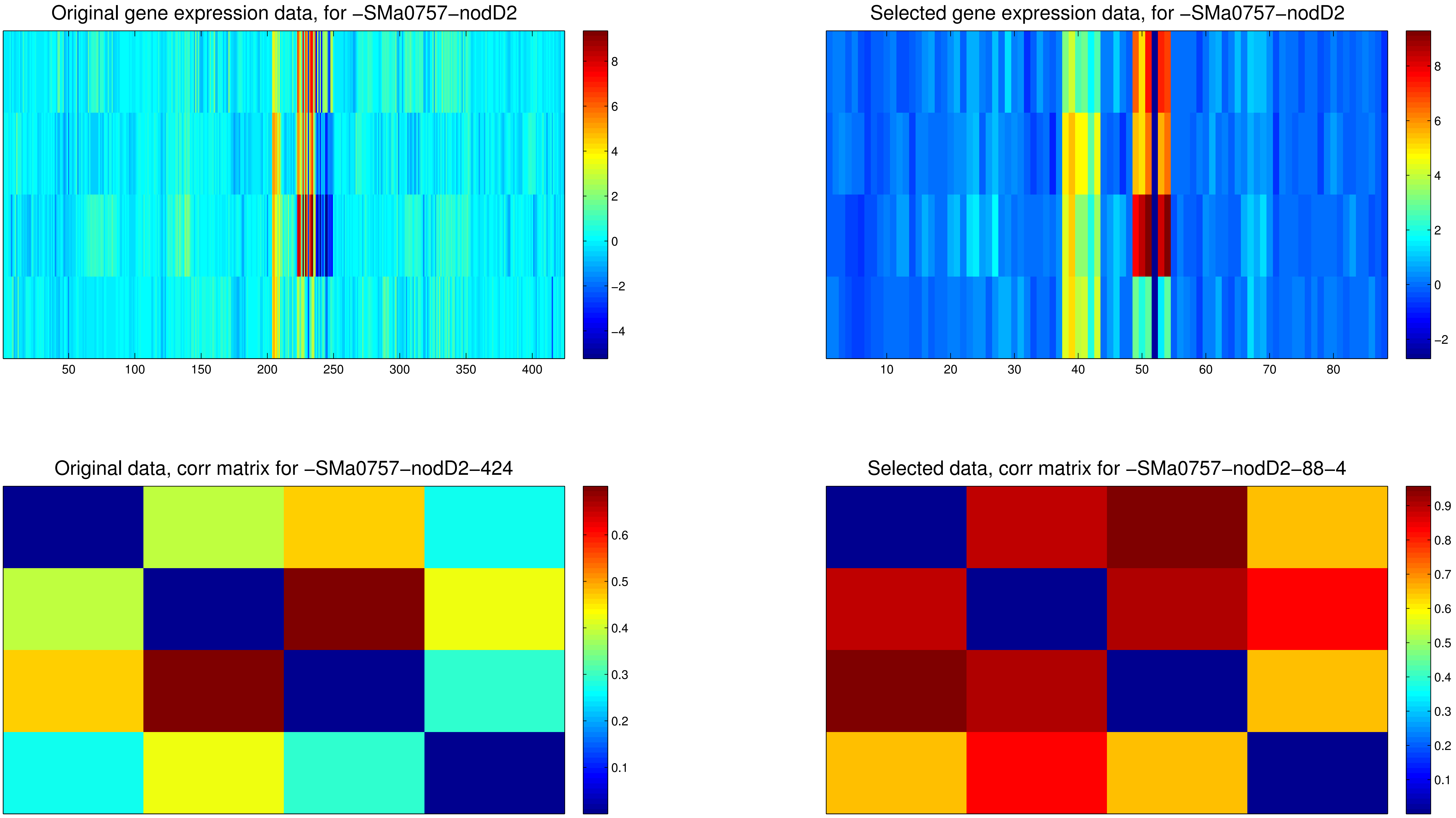
Original gene expression data, for −SMa0757−nodD2
50 100 150 200 250 300 350 400
8 6 4 2 0 −2 −4
Selected gene expression data, for −SMa0757−nodD2
10 20 30 40 50 60 70 80
8 6 4 2 0 −2
Original data, corr matrix for −SMa0757−nodD2−424
0.6 0.5 0.4 0.3 0.2 0.1
Selected data, corr matrix for −SMa0757−nodD2−88−4
0.9 0.8 0.7 0.6 0.5 0.4 0.3 0.2 0.1
