# Supplementary material for: Evolution of Intra-specific Regulatory Networks in a Multipartite Bacterial Genome
Source: PLoS Comput Biol. 2015 Sep 4;11(9):e1004478. doi: 10.1371/journal.pcbi.1004478 (PMC4560400; doi:10.1371/journal.pcbi.1004478)

Original gene expression data, for −SMc03806−glnK

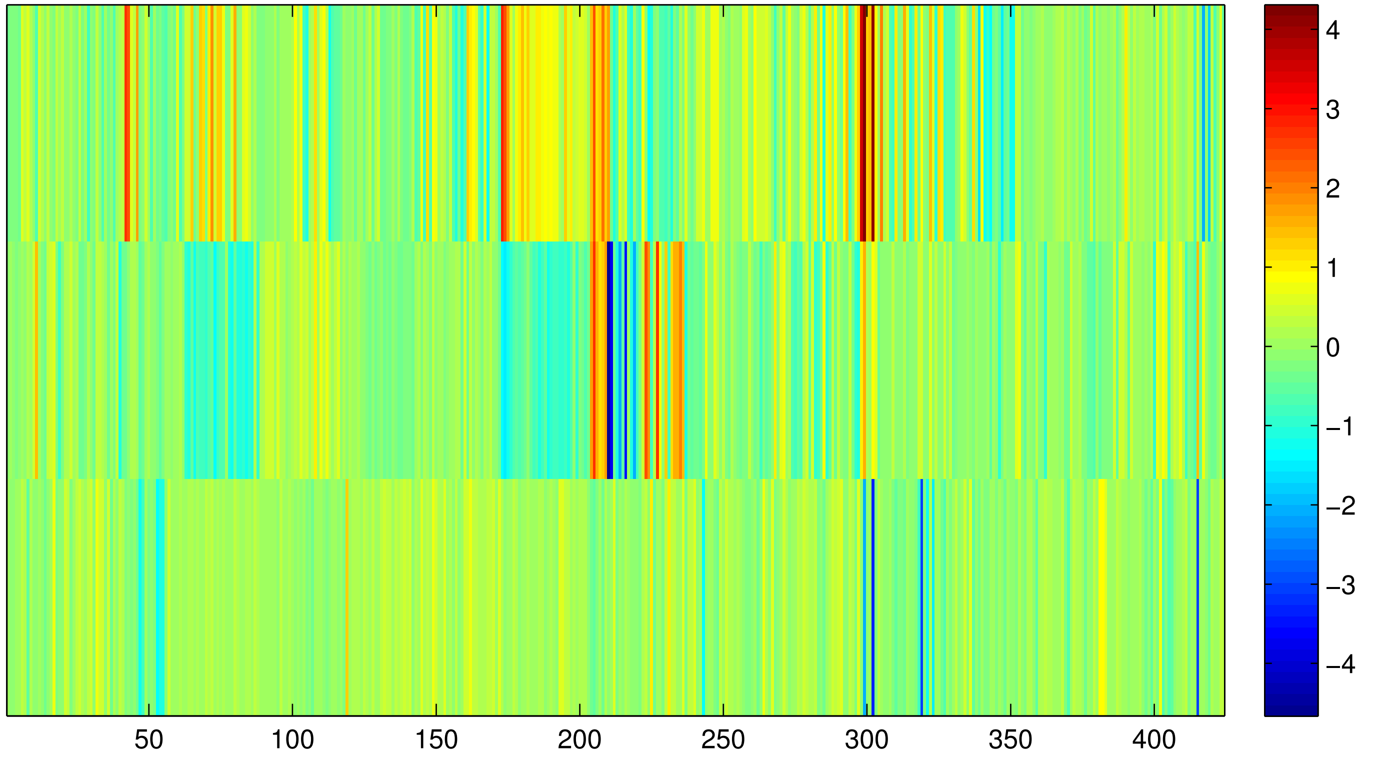

Selected gene expression data, for −SMc03806−glnK

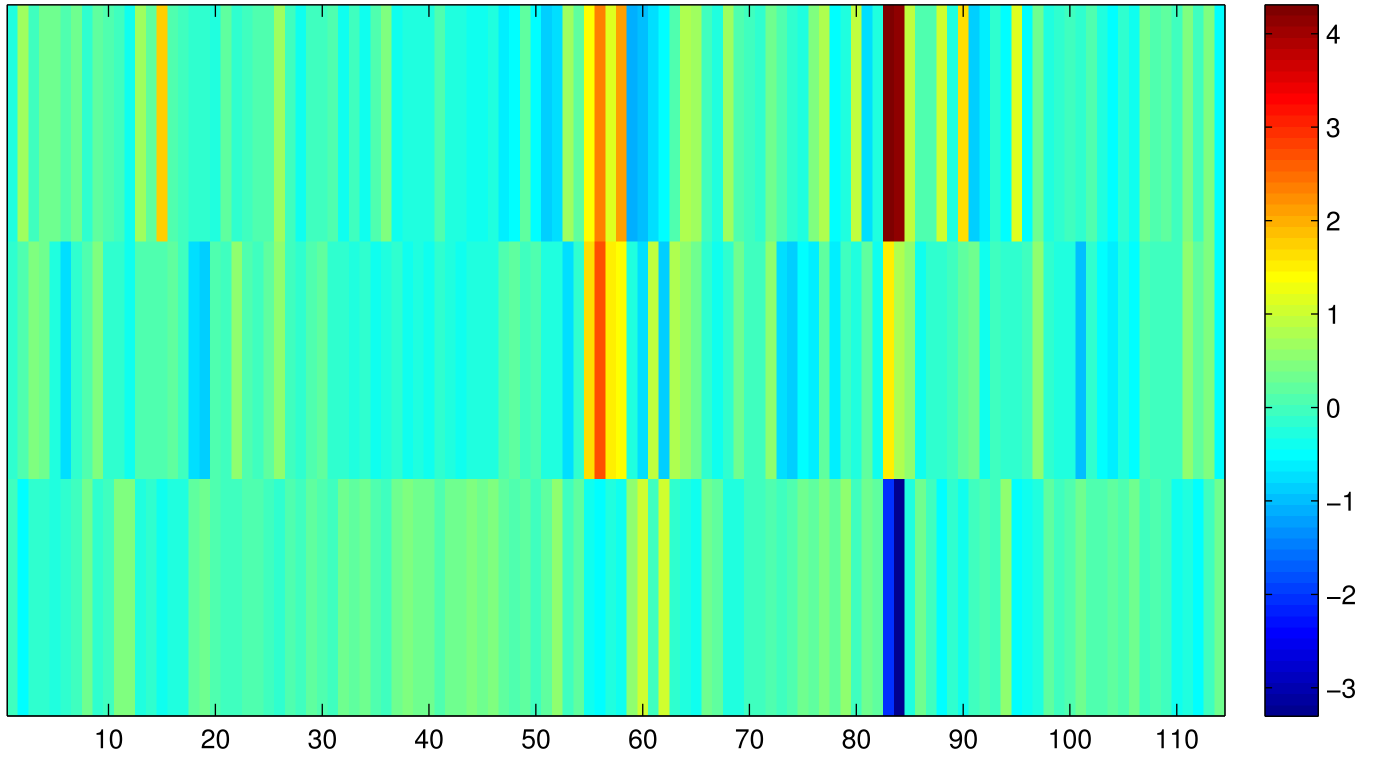

Original data, corr matrix for −SMc03806−glnK−424

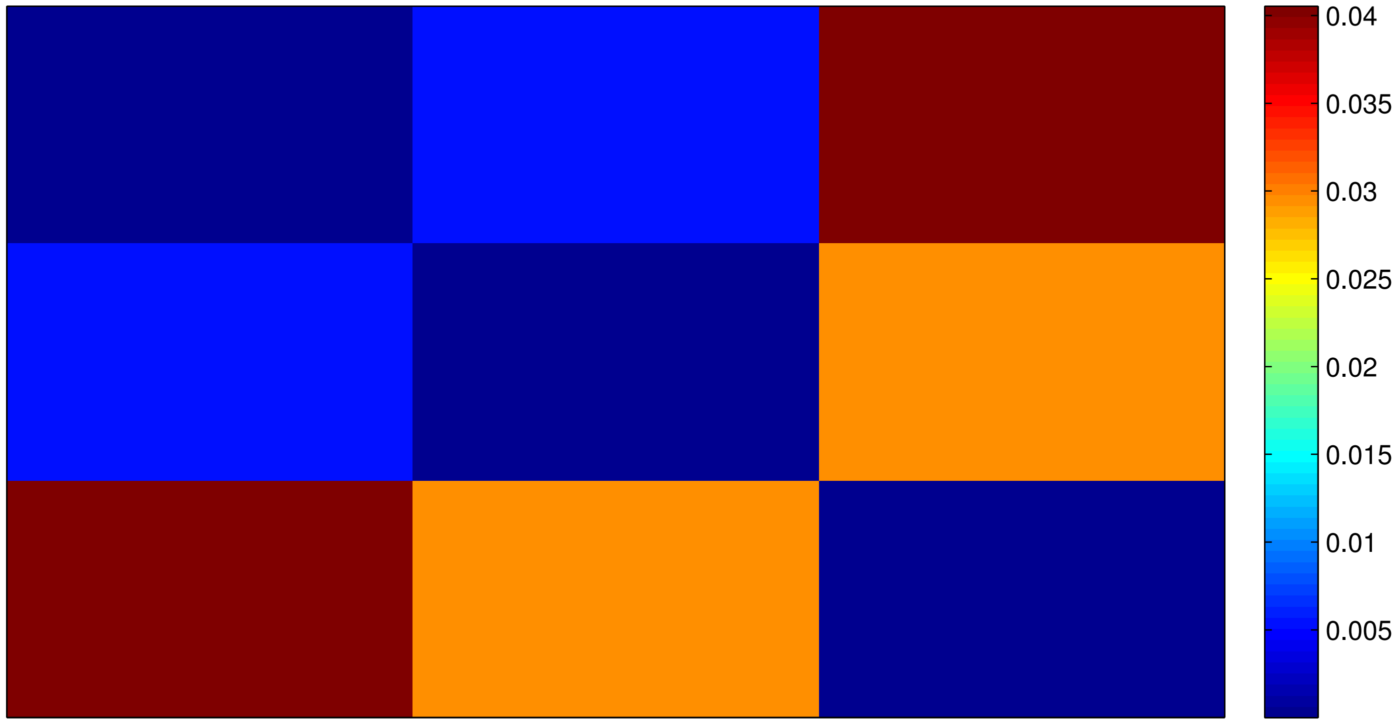

Selected data, corr matrix for −SMc03806−glnK−114−3

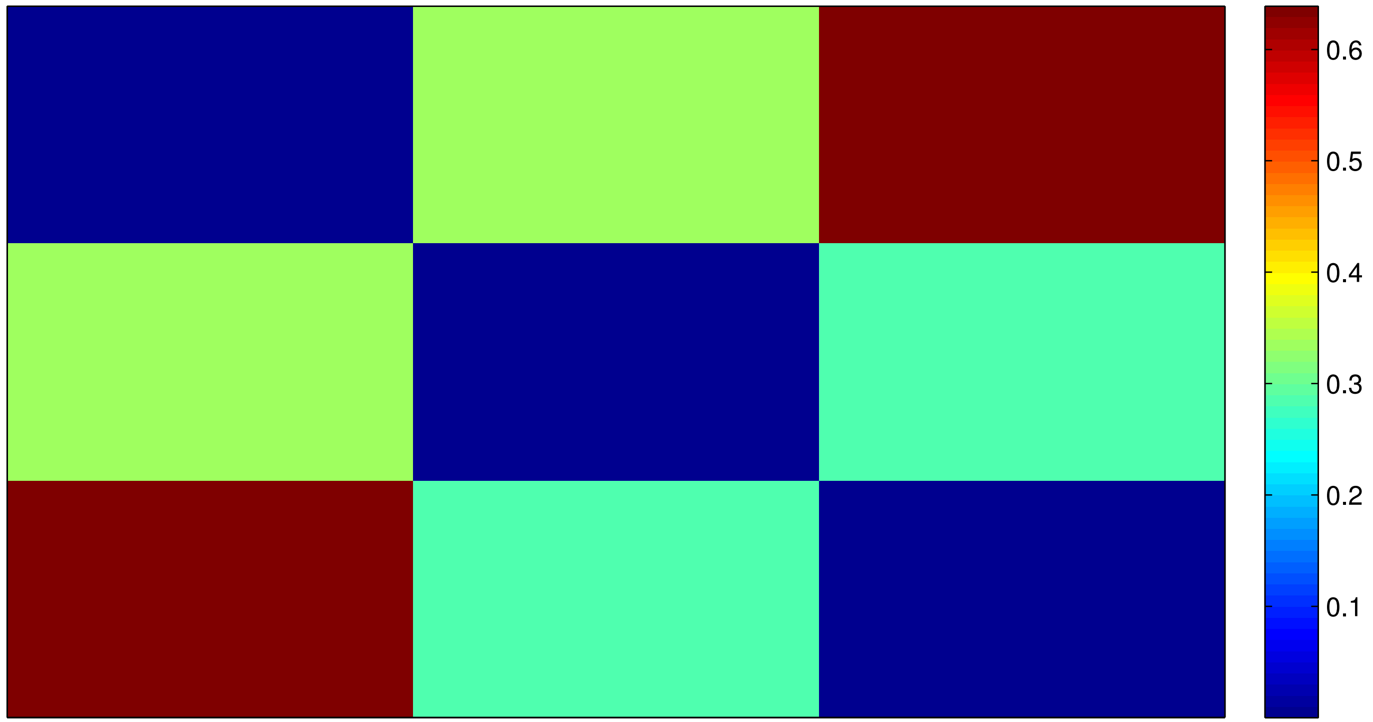

Supplement: S2 Material — (ZIP) [file pcbi.1004478.s002.zip › predictions_40_or_more/SMc03806.pdf]
