# Supplementary material for: Evolution of Intra-specific Regulatory Networks in a Multipartite Bacterial Genome
Source: PLoS Comput Biol. 2015 Sep 4;11(9):e1004478. doi: 10.1371/journal.pcbi.1004478 (PMC4560400; doi:10.1371/journal.pcbi.1004478)
Supplement: S2 Material — (ZIP) [file pcbi.1004478.s002.zip › predictions_40_or_more/SMc01164.pdf]

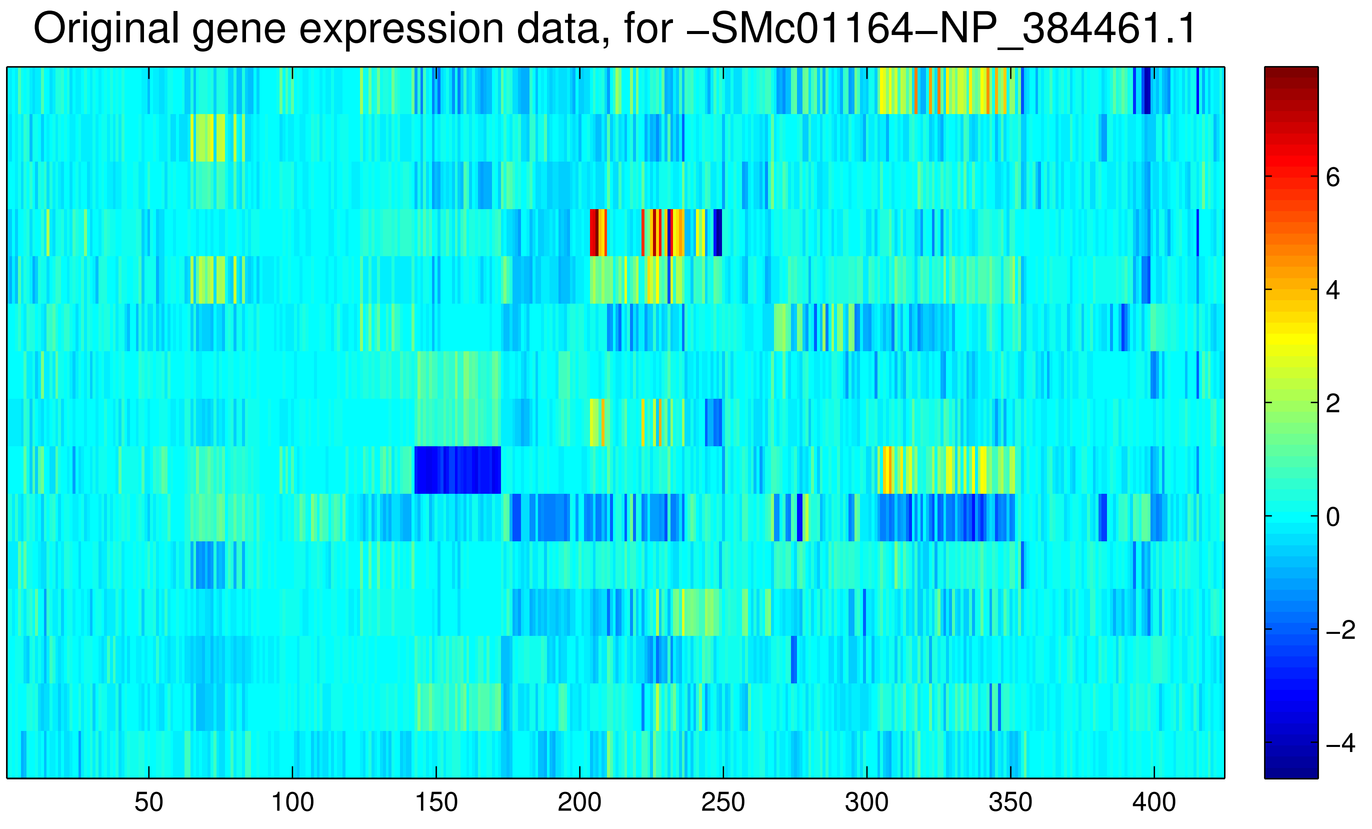

Original gene expression data, for −SMc01164−NP_384461.1
50
100
150
200
250
300
350
400
6
4
2
0
−2
−4

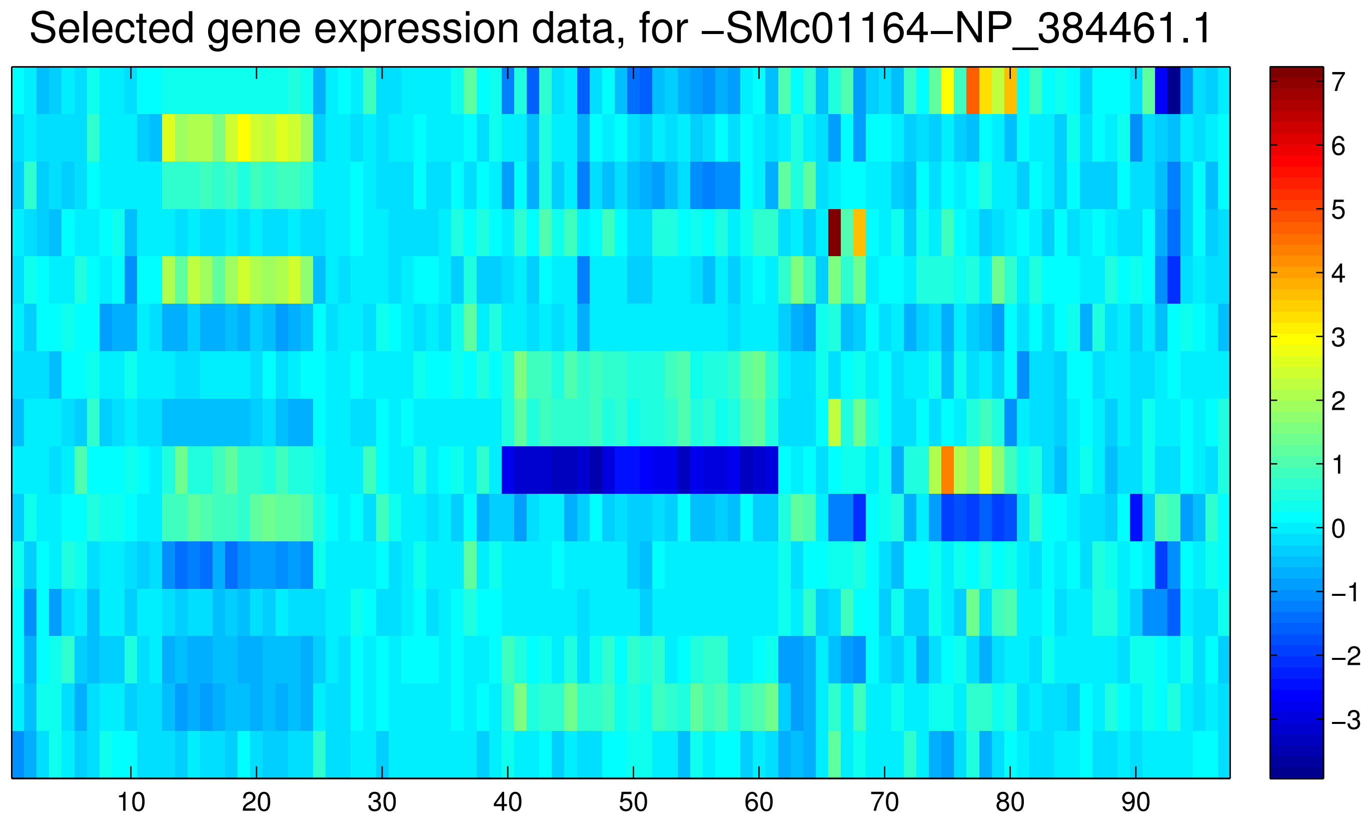

Selected gene expression data, for −SMc01164−NP_384461.1
10
20
30
40
50
60
70
80
90
7
6
5
4
3
2
1
0
−1
−2
−3

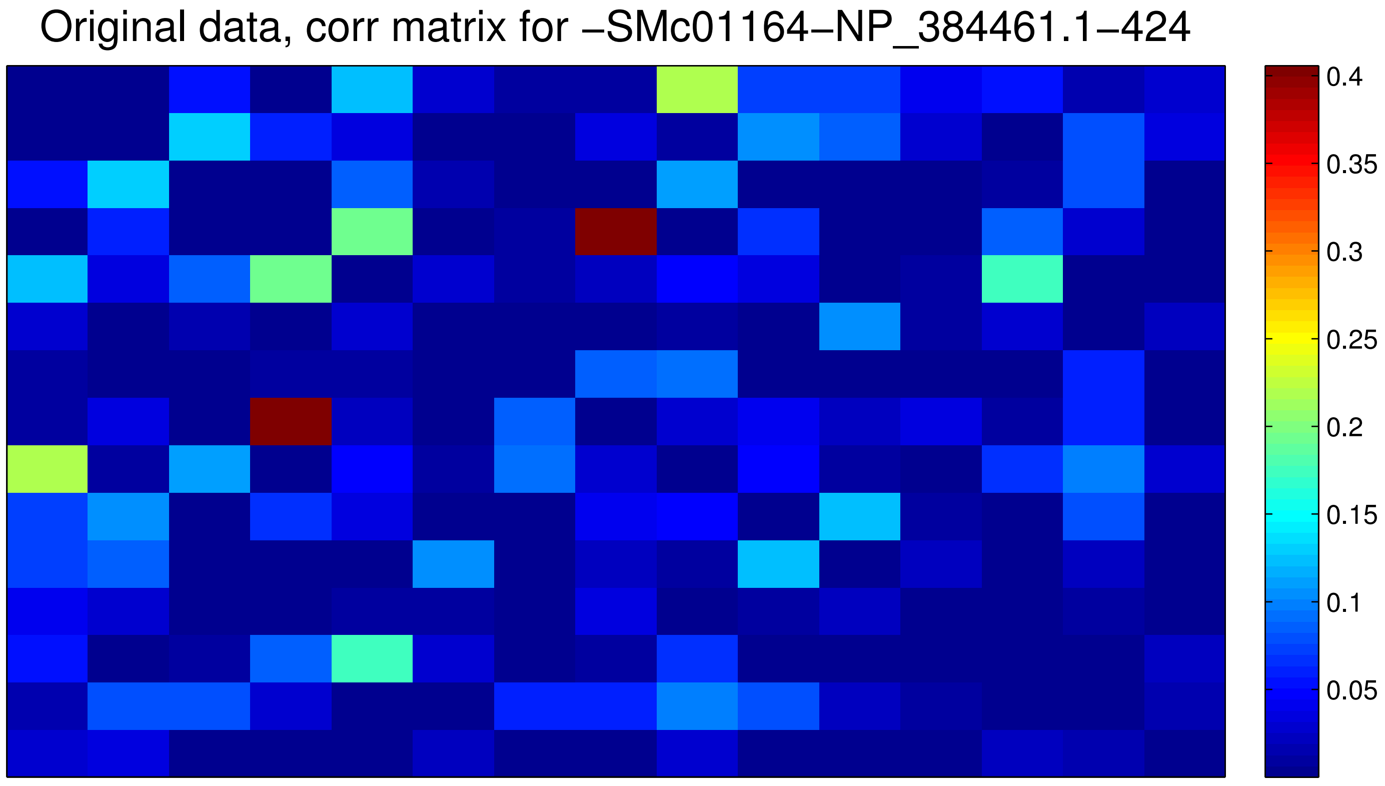

Original data, corr matrix for −SMc01164−NP_384461.1−424
0.4
0.35
0.3
0.25
0.2
0.15
0.1
0.05

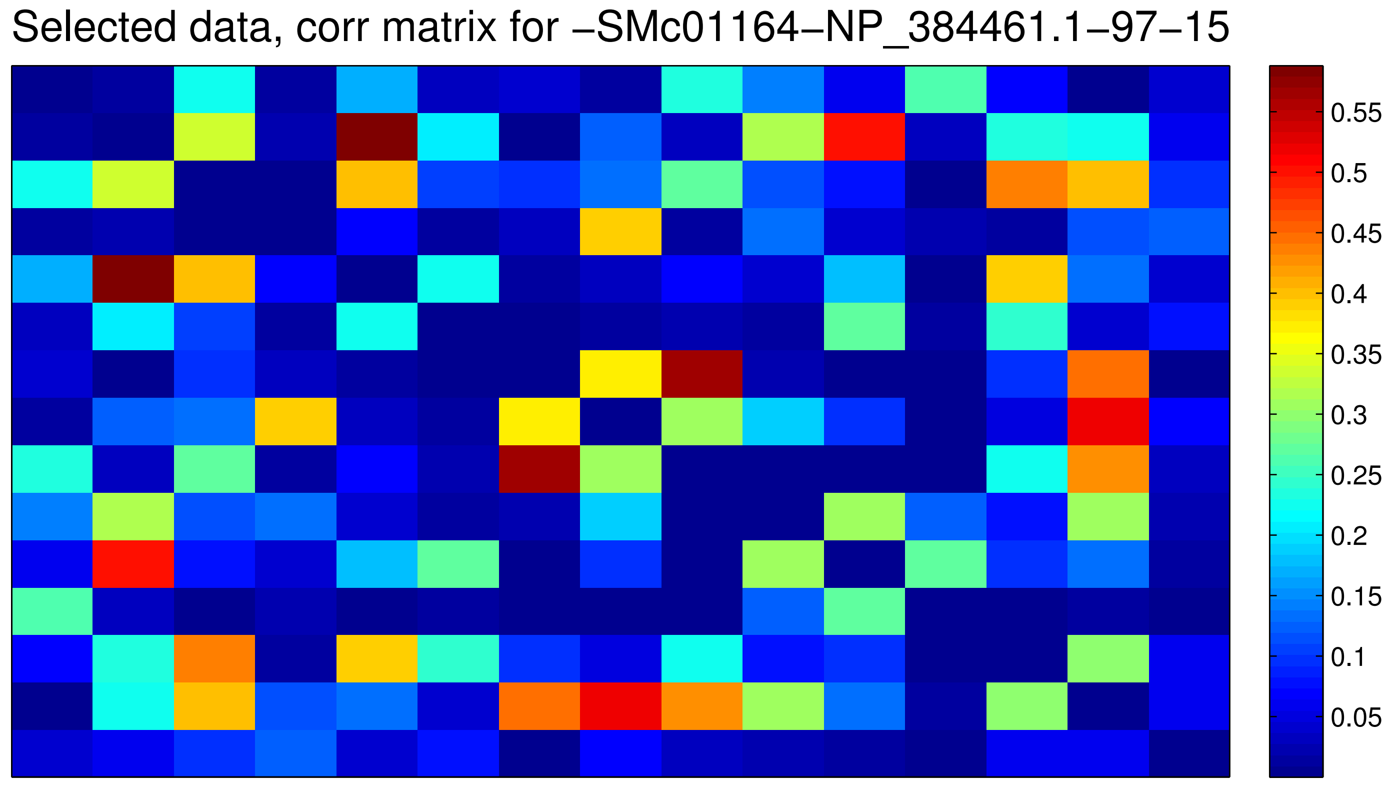

Selected data, corr matrix for −SMc01164−NP_384461.1−97−15
0.55
0.5
0.45
0.4
0.35
0.3
0.25
0.2
0.15
0.1
0.05
