# Supplementary material for: Evolution of Intra-specific Regulatory Networks in a Multipartite Bacterial Genome
Source: PLoS Comput Biol. 2015 Sep 4;11(9):e1004478. doi: 10.1371/journal.pcbi.1004478 (PMC4560400; doi:10.1371/journal.pcbi.1004478)
Supplement: S2 Material — (ZIP) [file pcbi.1004478.s002.zip › predictions_40_or_more/SMb21598.pdf]

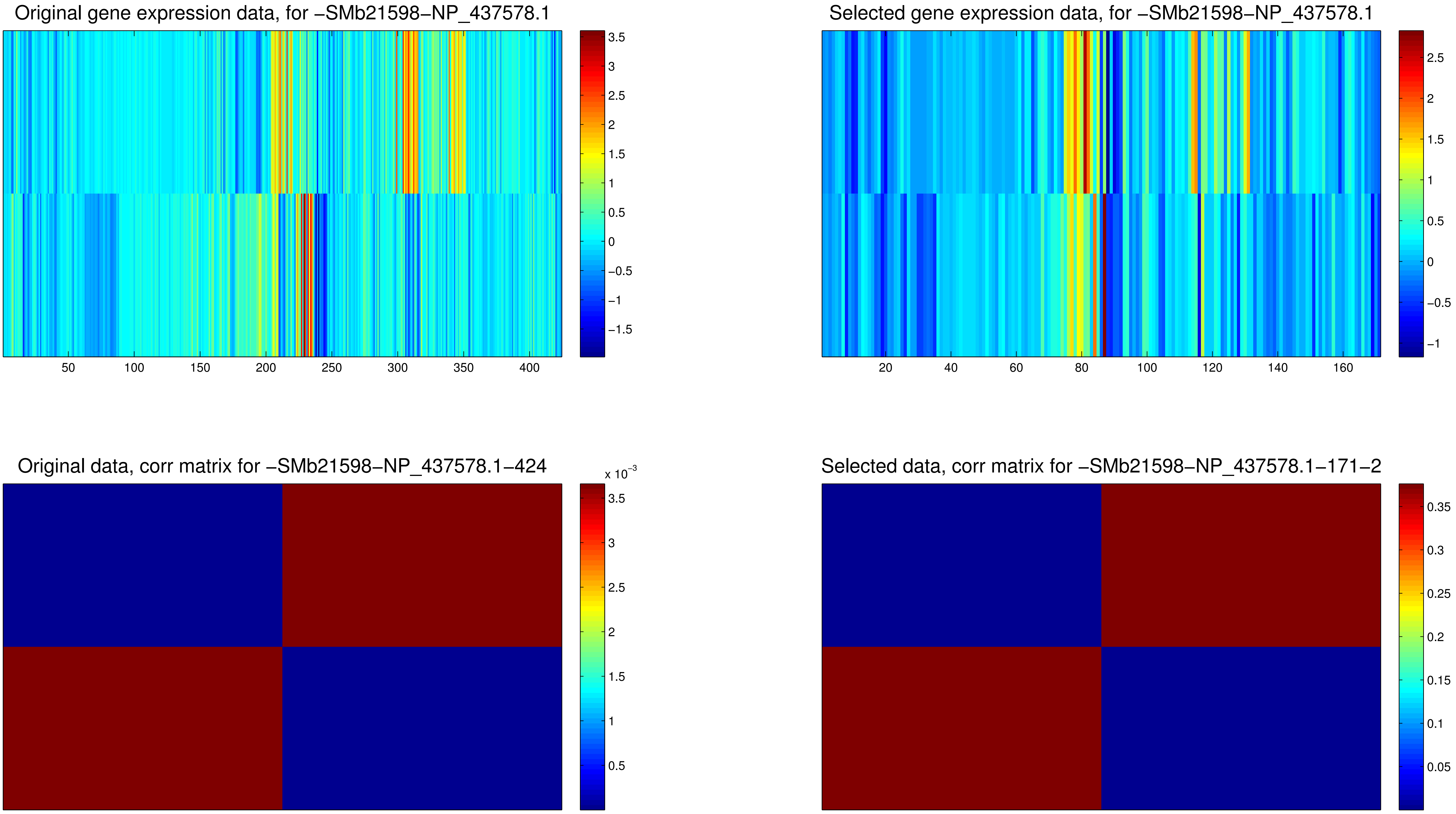

Original gene expression data, for −SMb21598−NP_437578.1
Selected gene expression data, for −SMb21598−NP_437578.1
Original data, corr matrix for −SMb21598−NP_437578.1−424
Selected data, corr matrix for −SMb21598−NP_437578.1−171−2
x 10^−3
