# Supplementary material for: Evolution of Intra-specific Regulatory Networks in a Multipartite Bacterial Genome
Source: PLoS Comput Biol. 2015 Sep 4;11(9):e1004478. doi: 10.1371/journal.pcbi.1004478 (PMC4560400; doi:10.1371/journal.pcbi.1004478)
Supplement: S2 Material — (ZIP) [file pcbi.1004478.s002.zip › predictions_40_or_more/SMb21317.pdf]

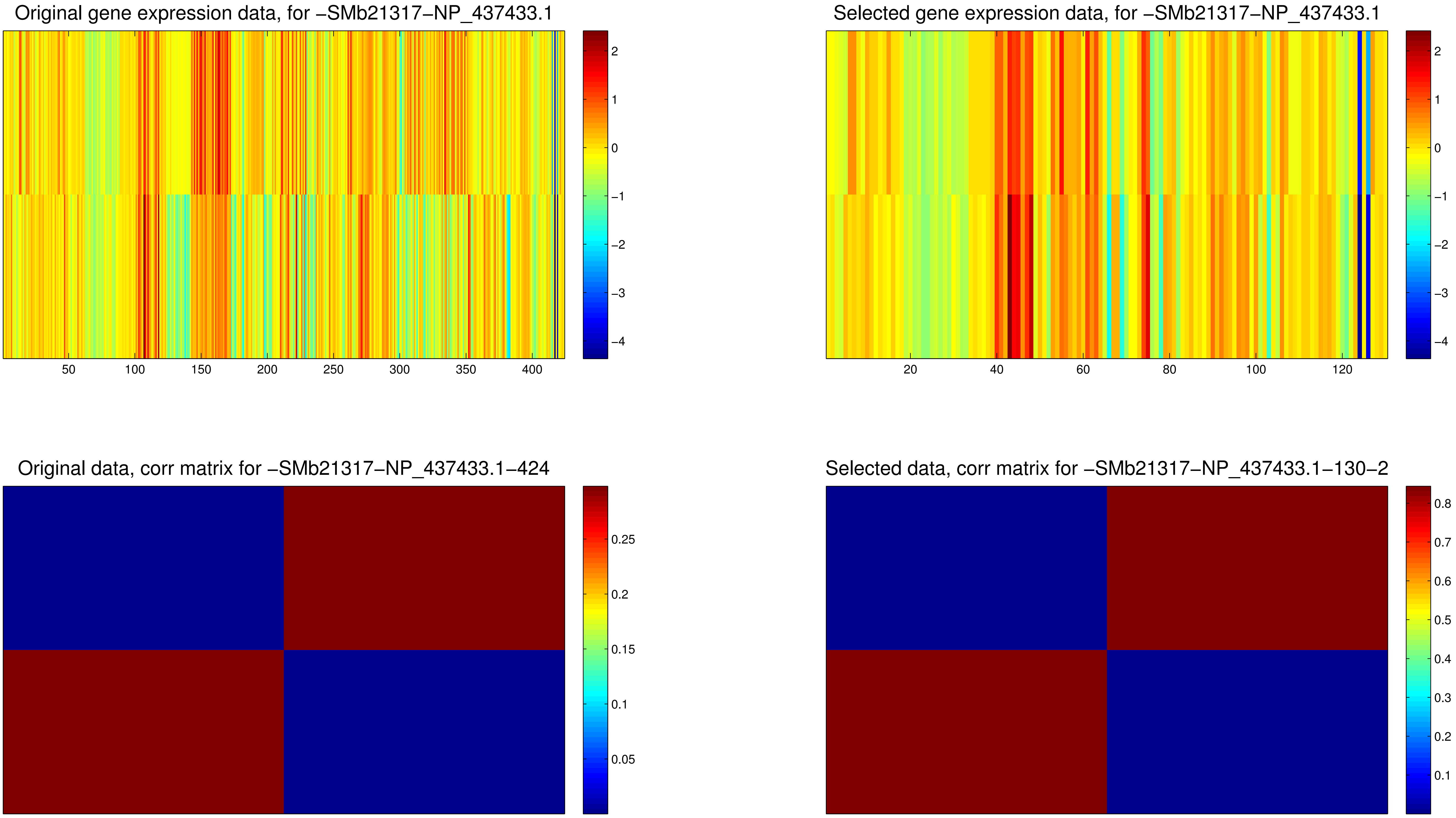
Original gene expression data, for −SMb21317−NP_437433.1
50
100
150
200
250
300
350
400
2
1
0
−1
−2
−3
−4
Selected gene expression data, for −SMb21317−NP_437433.1
20
40
60
80
100
120
2
1
0
−1
−2
−3
−4
Original data, corr matrix for −SMb21317−NP_437433.1−424
0.25
0.2
0.15
0.1
0.05
Selected data, corr matrix for −SMb21317−NP_437433.1−130−2
0.8
0.7
0.6
0.5
0.4
0.3
0.2
0.1
