# Supplementary material for: Evolution of Intra-specific Regulatory Networks in a Multipartite Bacterial Genome
Source: PLoS Comput Biol. 2015 Sep 4;11(9):e1004478. doi: 10.1371/journal.pcbi.1004478 (PMC4560400; doi:10.1371/journal.pcbi.1004478)
Supplement: S2 Material — (ZIP) [file pcbi.1004478.s002.zip › predictions_40_or_more/SMb20717.pdf]

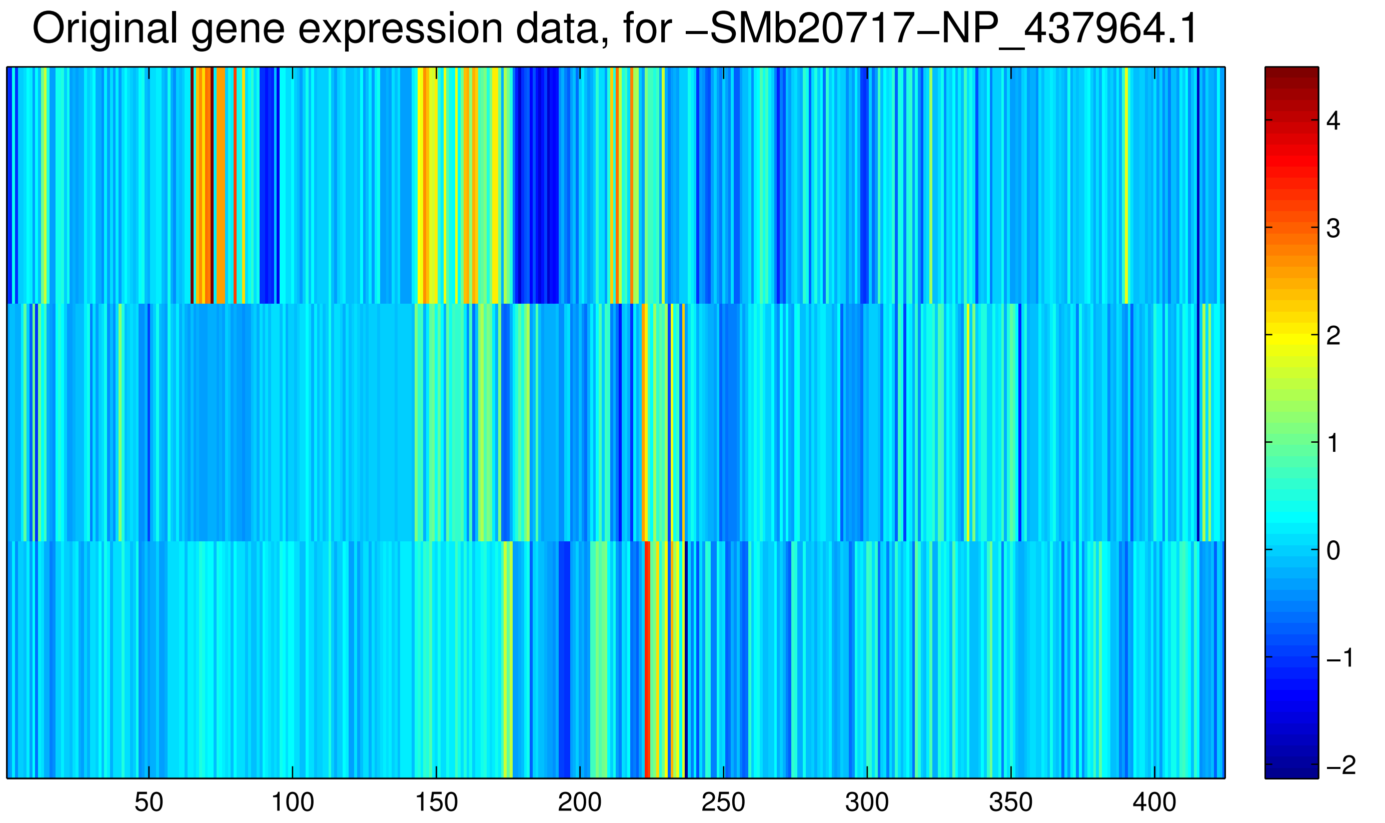
Original gene expression data, for −SMb20717−NP_437964.1
50
100
150
200
250
300
350
400
4
3
2
1
0
−1
−2

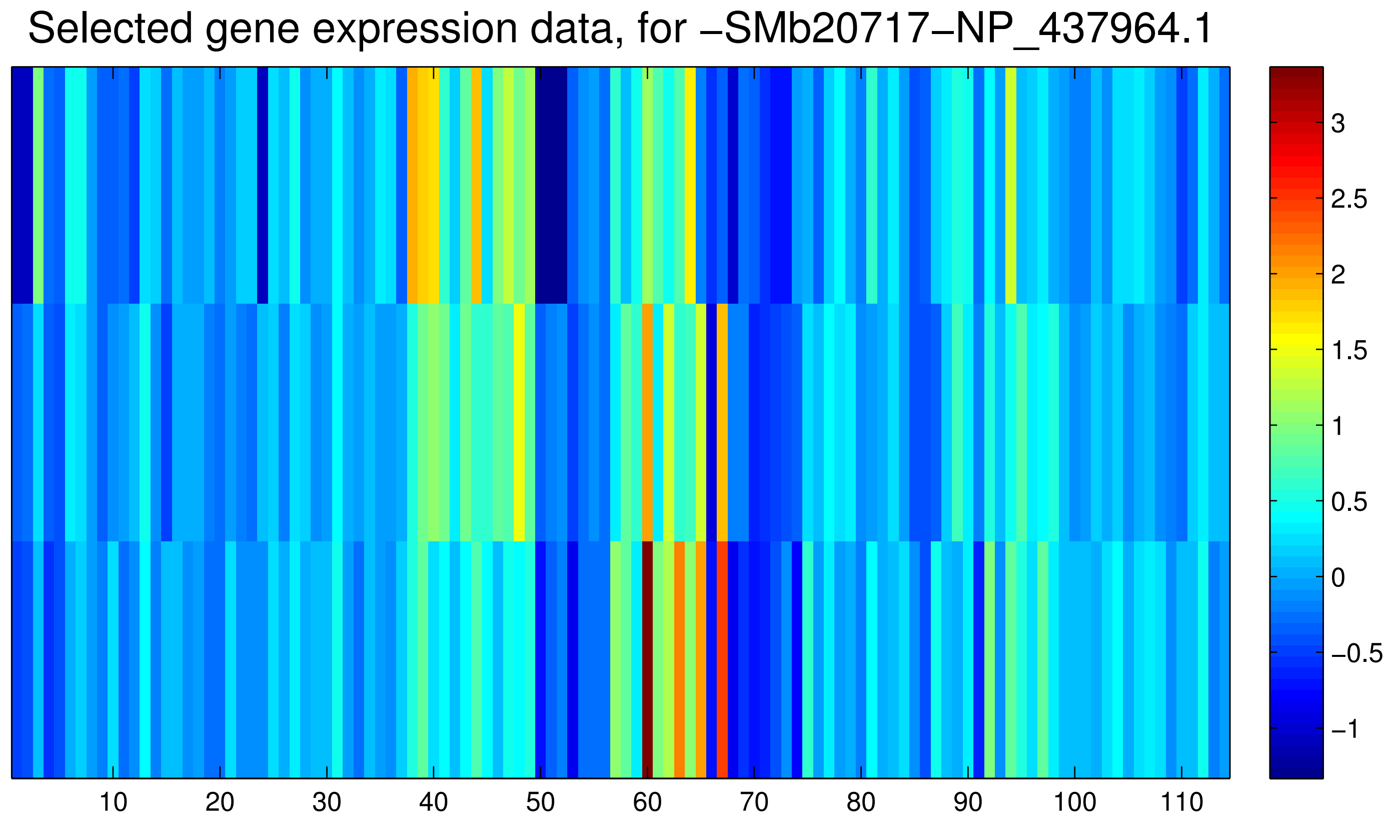
Selected gene expression data, for −SMb20717−NP_437964.1
10
20
30
40
50
60
70
80
90
100
110
3
2.5
2
1.5
1
0.5
0
−0.5
−1

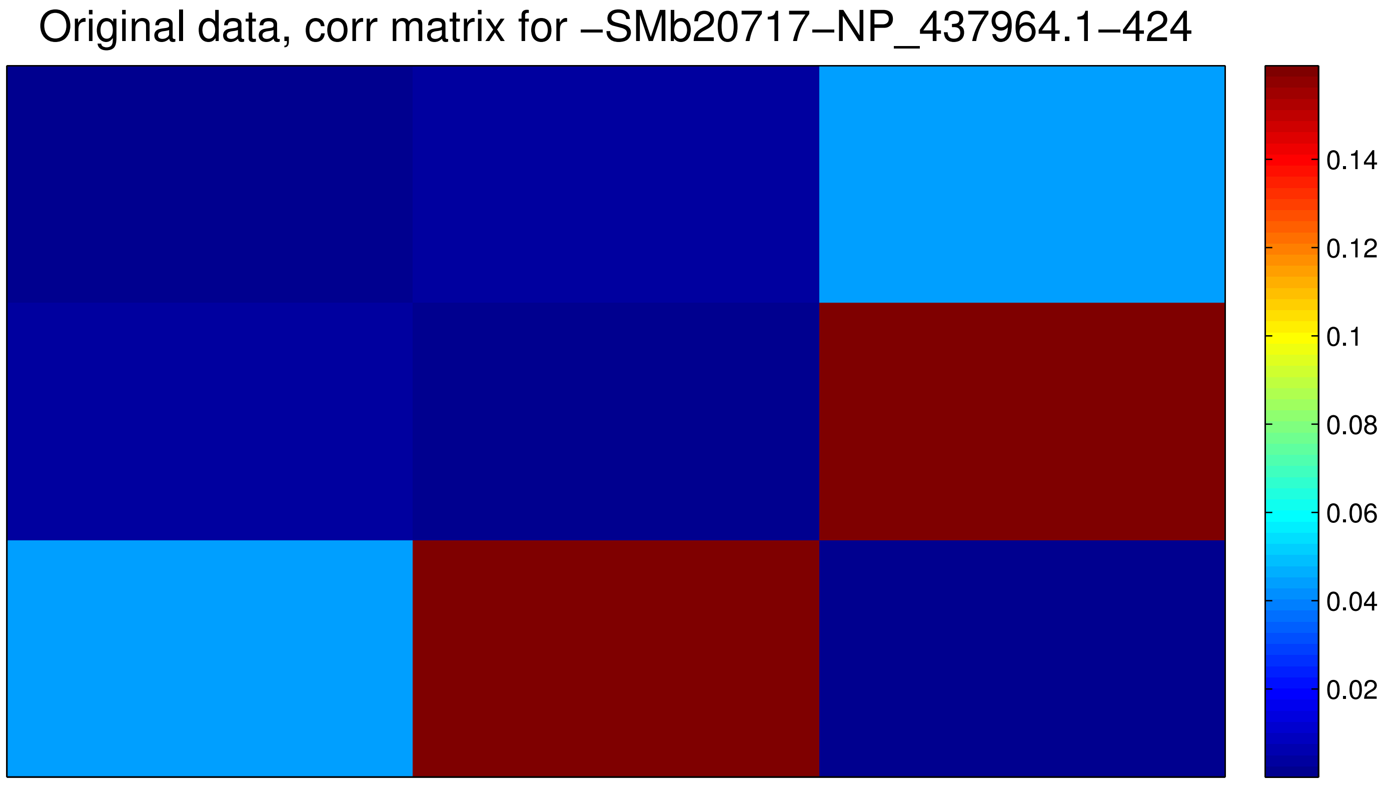
Original data, corr matrix for −SMb20717−NP_437964.1−424
0.14
0.12
0.1
0.08
0.06
0.04
0.02

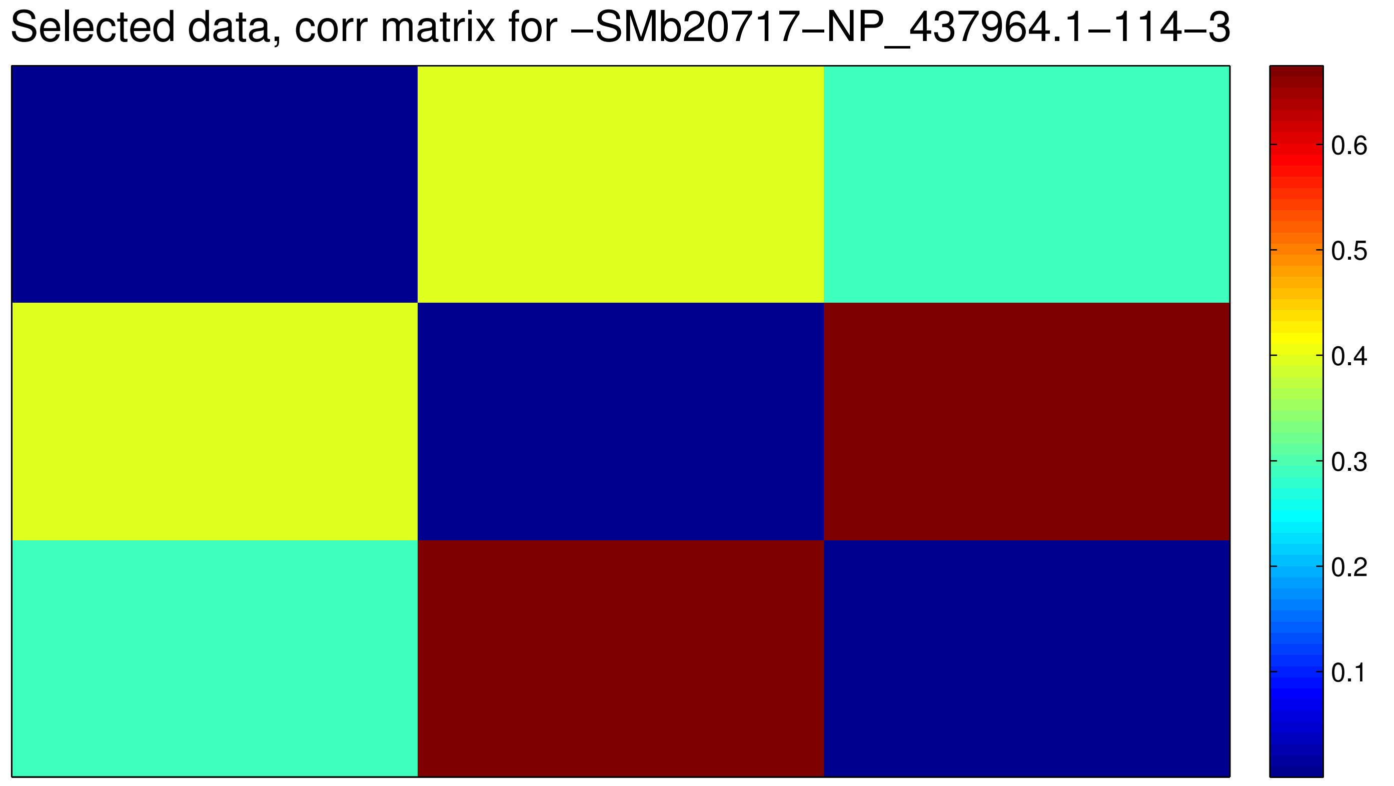
Selected data, corr matrix for −SMb20717−NP_437964.1−114−3
0.6
0.5
0.4
0.3
0.2
0.1
