# Supplementary material for: Evolution of Intra-specific Regulatory Networks in a Multipartite Bacterial Genome
Source: PLoS Comput Biol. 2015 Sep 4;11(9):e1004478. doi: 10.1371/journal.pcbi.1004478 (PMC4560400; doi:10.1371/journal.pcbi.1004478)
Supplement: S2 Material — (ZIP) [file pcbi.1004478.s002.zip › predictions_40_or_more/SMb20580.pdf]

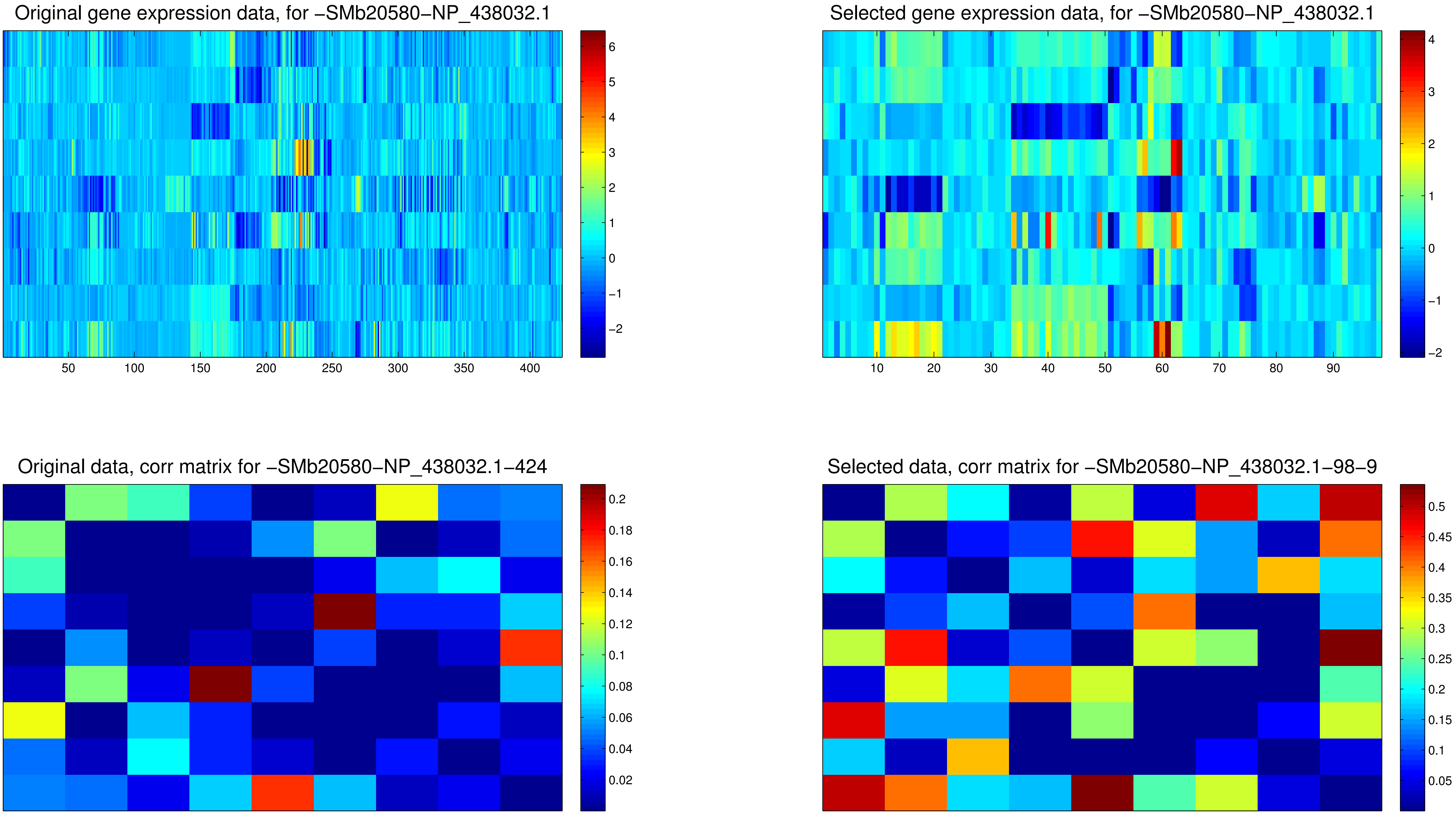
Original gene expression data, for –SMb20580–NP_438032.1
Selected gene expression data, for –SMb20580–NP_438032.1
Original data, corr matrix for –SMb20580–NP_438032.1–424
Selected data, corr matrix for –SMb20580–NP_438032.1–98–9
50 100 150 200 250 300 350 400
10 20 30 40 50 60 70 80 90
6 5 4 3 2 1 0 −1 −2
4 3 2 1 0 −1 −2
0.2 0.18 0.16 0.14 0.12 0.1 0.08 0.06 0.04 0.02
0.5 0.45 0.4 0.35 0.3 0.25 0.2 0.15 0.1 0.05
