# Supplementary material for: Evolution of Intra-specific Regulatory Networks in a Multipartite Bacterial Genome
Source: PLoS Comput Biol. 2015 Sep 4;11(9):e1004478. doi: 10.1371/journal.pcbi.1004478 (PMC4560400; doi:10.1371/journal.pcbi.1004478)
Supplement: S2 Material — (ZIP) [file pcbi.1004478.s002.zip › predictions_40_or_more/SMb20366.pdf]

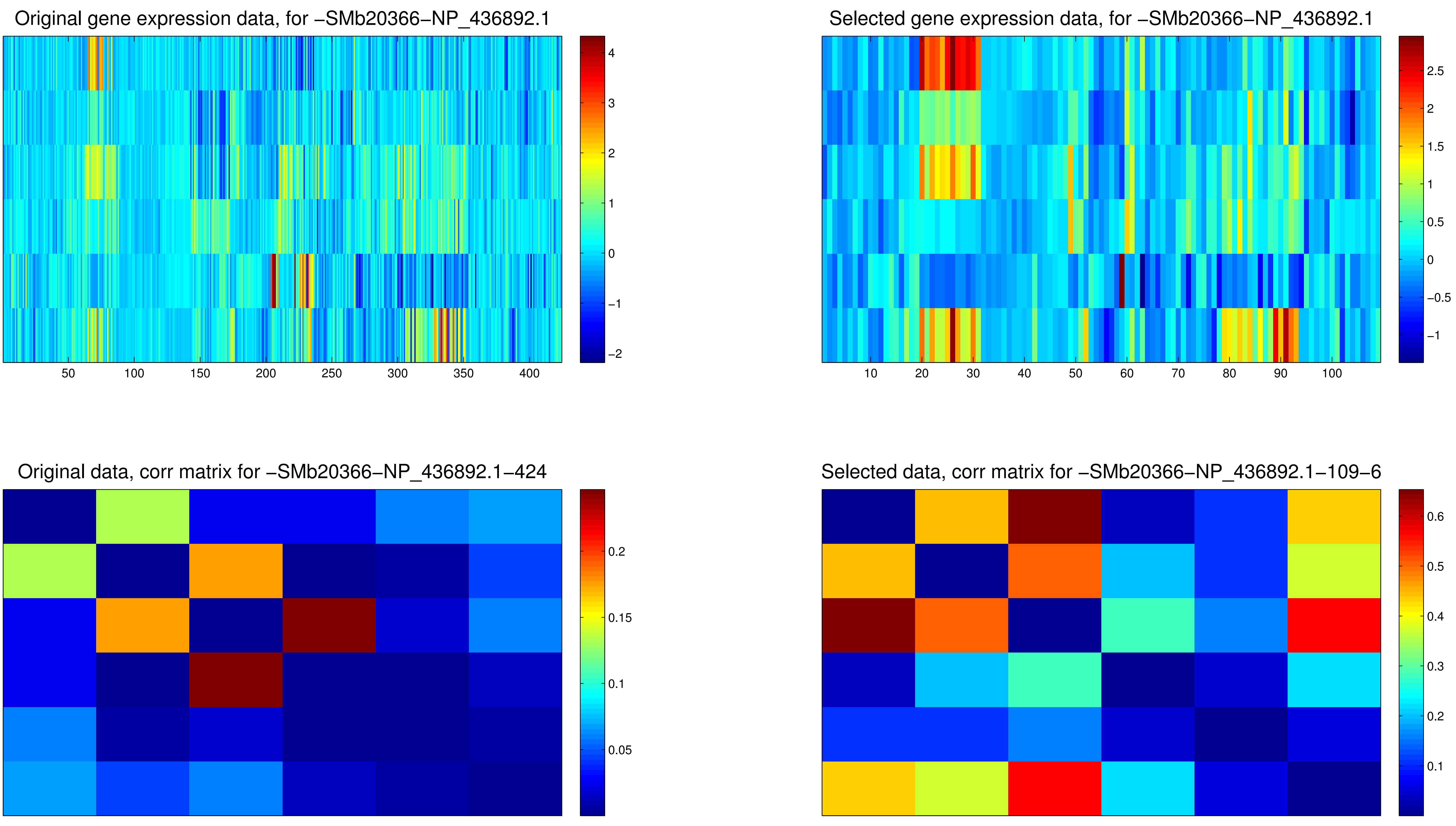

Original gene expression data, for −SMb20366−NP_436892.1
50 100 150 200 250 300 350 400
4 3 2 1 0 −1 −2
Selected gene expression data, for −SMb20366−NP_436892.1
10 20 30 40 50 60 70 80 90 100
2.5 2 1.5 1 0.5 0 −0.5 −1
Original data, corr matrix for −SMb20366−NP_436892.1−424
0.2 0.15 0.1 0.05
Selected data, corr matrix for −SMb20366−NP_436892.1−109−6
0.6 0.5 0.4 0.3 0.2 0.1
