# Supplementary material for: Evolution of Intra-specific Regulatory Networks in a Multipartite Bacterial Genome
Source: PLoS Comput Biol. 2015 Sep 4;11(9):e1004478. doi: 10.1371/journal.pcbi.1004478 (PMC4560400; doi:10.1371/journal.pcbi.1004478)
Supplement: S2 Material — (ZIP) [file pcbi.1004478.s002.zip › predictions_40_or_more/SMb20324.pdf]

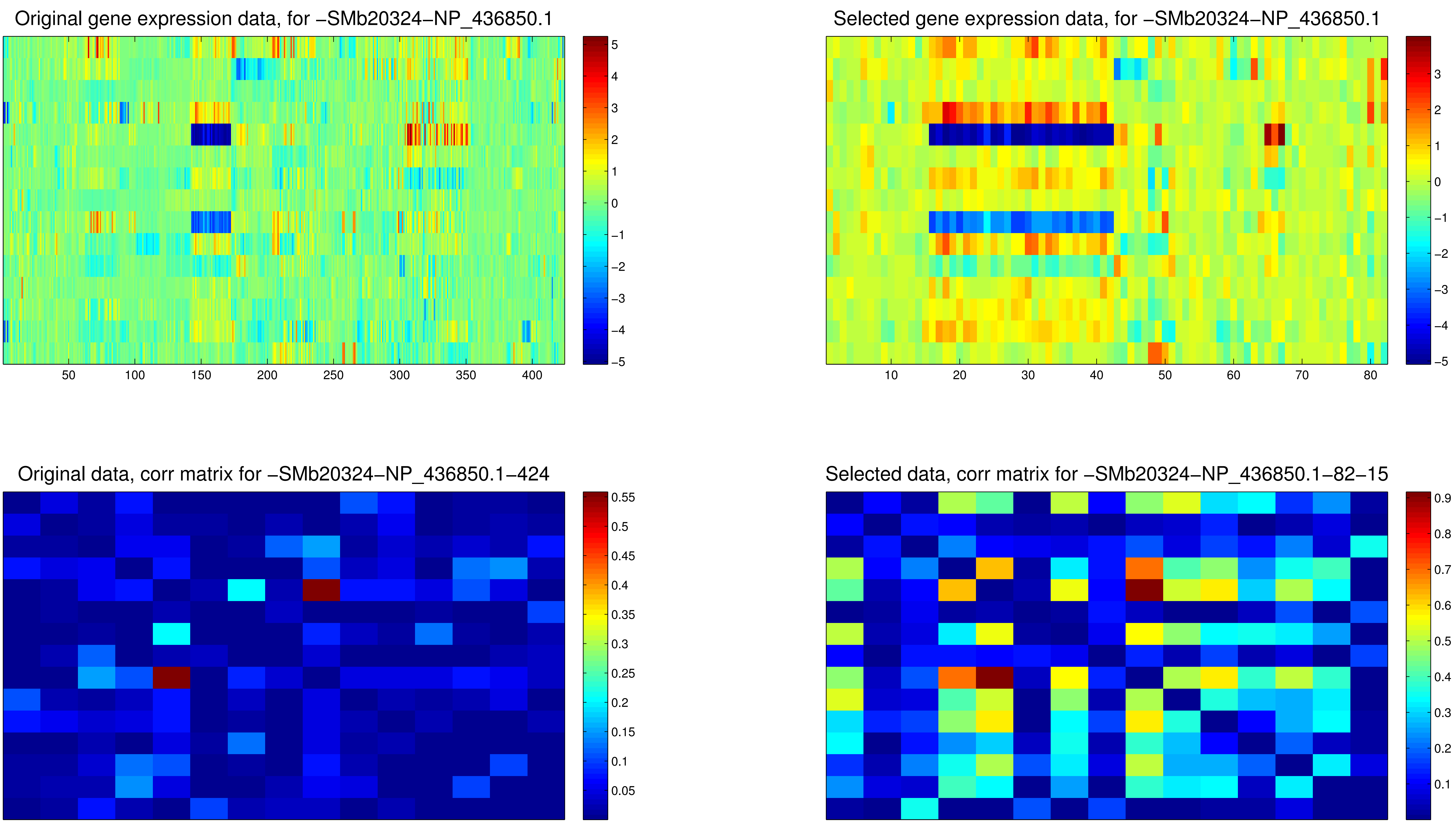
Original gene expression data, for −SMb20324−NP_436850.1
50 100 150 200 250 300 350 400
5 4 3 2 1 0 −1 −2 −3 −4 −5
Selected gene expression data, for −SMb20324−NP_436850.1
10 20 30 40 50 60 70 80
3 2 1 0 −1 −2 −3 −4 −5
Original data, corr matrix for −SMb20324−NP_436850.1−424
0.55 0.5 0.45 0.4 0.35 0.3 0.25 0.2 0.15 0.1 0.05
Selected data, corr matrix for −SMb20324−NP_436850.1−82−15
0.9 0.8 0.7 0.6 0.5 0.4 0.3 0.2 0.1
