# Supplementary material for: Evolution of Intra-specific Regulatory Networks in a Multipartite Bacterial Genome
Source: PLoS Comput Biol. 2015 Sep 4;11(9):e1004478. doi: 10.1371/journal.pcbi.1004478 (PMC4560400; doi:10.1371/journal.pcbi.1004478)
Supplement: S2 Material — (ZIP) [file pcbi.1004478.s002.zip › predictions_40_or_more/SMb20290.pdf]

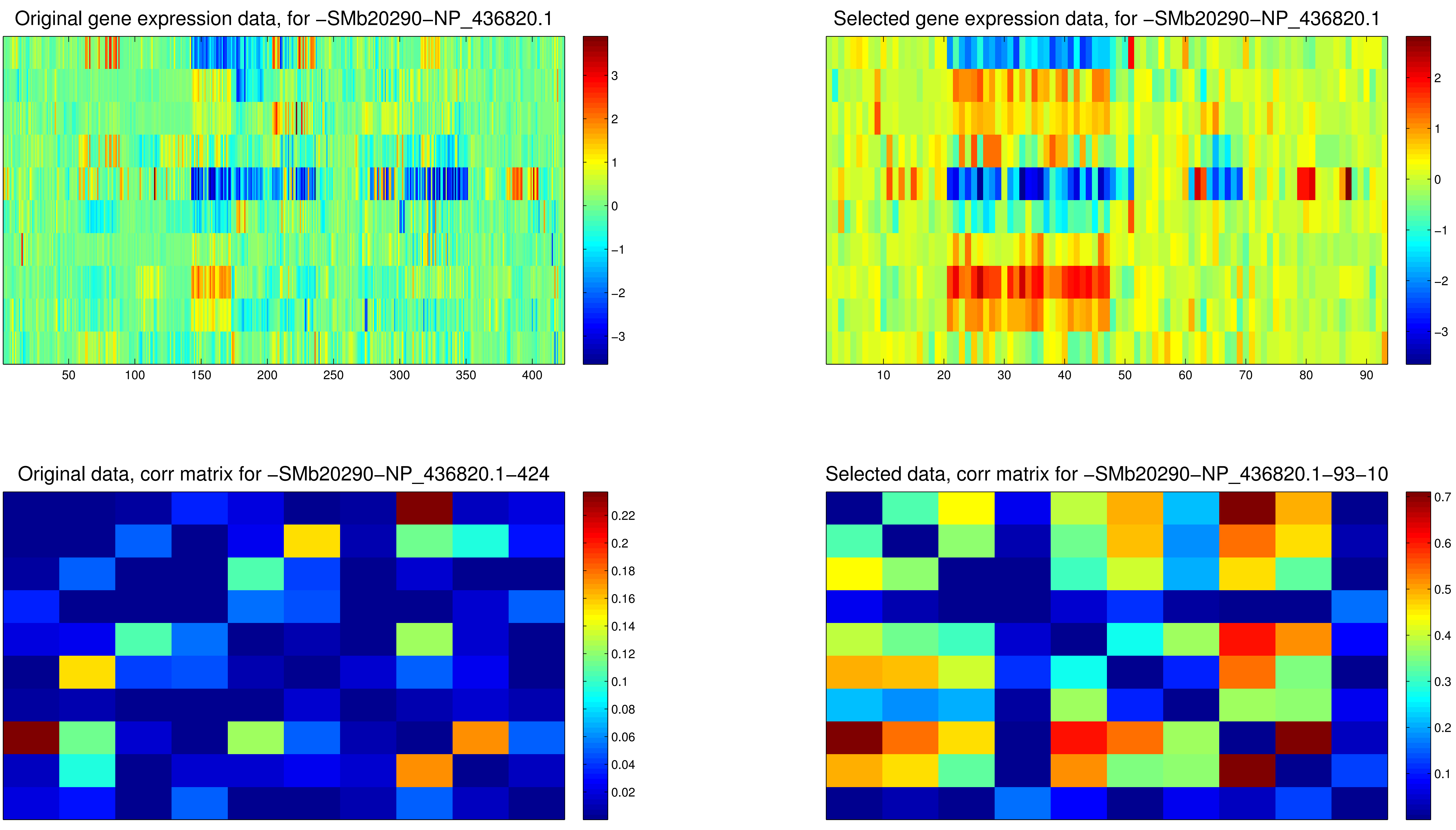
Original gene expression data, for –SMb20290–NP_436820.1
50
100
150
200
250
300
350
400
3
2
1
0
–1
–2
–3
Selected gene expression data, for –SMb20290–NP_436820.1
10
20
30
40
50
60
70
80
90
2
1
0
–1
–2
–3
Original data, corr matrix for –SMb20290–NP_436820.1–424
0.22
0.2
0.18
0.16
0.14
0.12
0.1
0.08
0.06
0.04
0.02
Selected data, corr matrix for –SMb20290–NP_436820.1–93–10
0.7
0.6
0.5
0.4
0.3
0.2
0.1
