# Supplementary material for: Evolution of Intra-specific Regulatory Networks in a Multipartite Bacterial Genome
Source: PLoS Comput Biol. 2015 Sep 4;11(9):e1004478. doi: 10.1371/journal.pcbi.1004478 (PMC4560400; doi:10.1371/journal.pcbi.1004478)
Supplement: S2 Material — (ZIP) [file pcbi.1004478.s002.zip › predictions_40_or_more/SMa1225.pdf]

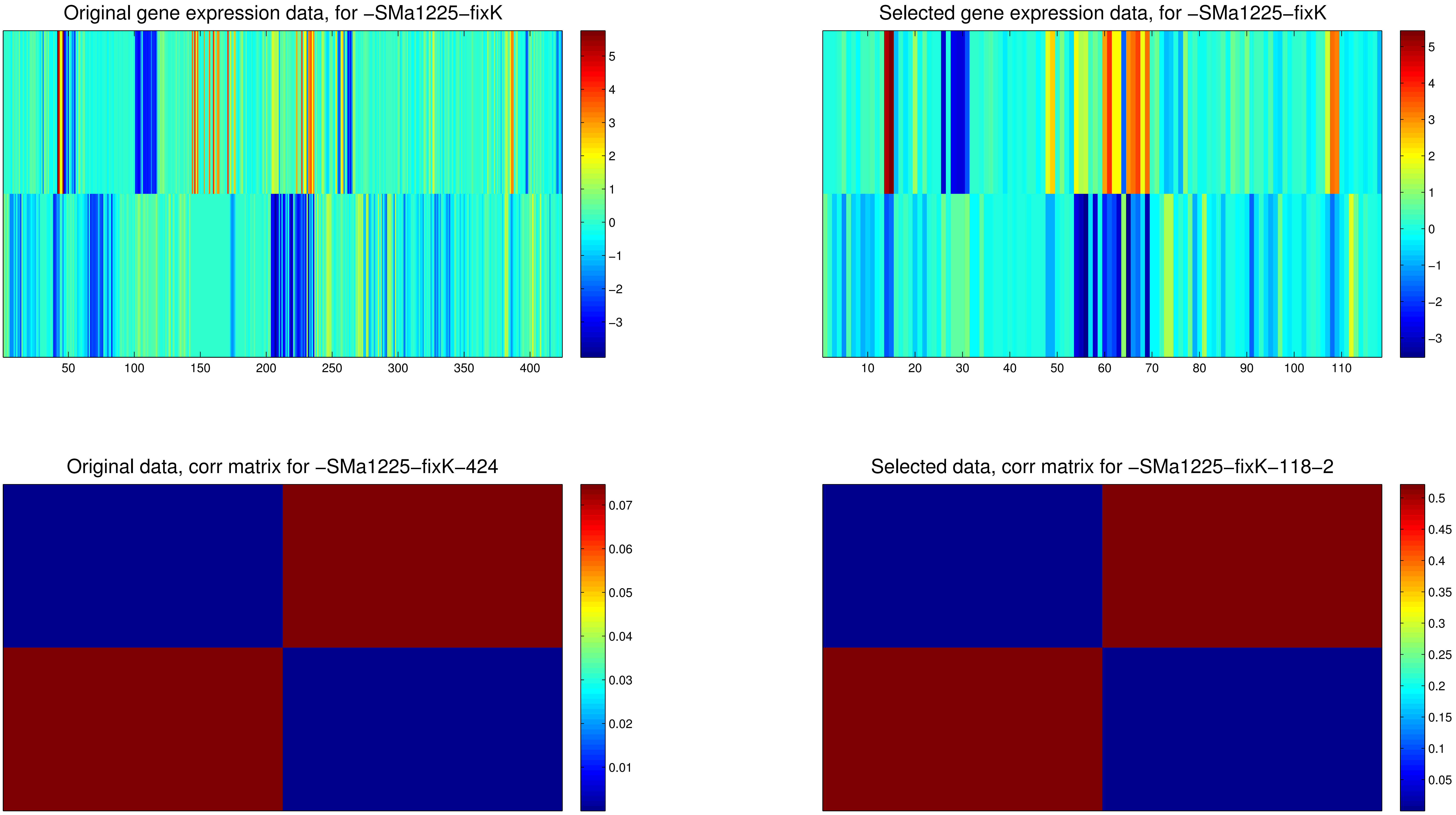

Original gene expression data, for −SMa1225−fixK
50 100 150 200 250 300 350 400
5 4 3 2 1 0 −1 −2 −3
Selected gene expression data, for −SMa1225−fixK
10 20 30 40 50 60 70 80 90 100 110
5 4 3 2 1 0 −1 −2 −3
Original data, corr matrix for −SMa1225−fixK−424
0.07 0.06 0.05 0.04 0.03 0.02 0.01
Selected data, corr matrix for −SMa1225−fixK−118−2
0.5 0.45 0.4 0.35 0.3 0.25 0.2 0.15 0.1 0.05
