# Supplementary material for: Evolution of Intra-specific Regulatory Networks in a Multipartite Bacterial Genome
Source: PLoS Comput Biol. 2015 Sep 4;11(9):e1004478. doi: 10.1371/journal.pcbi.1004478 (PMC4560400; doi:10.1371/journal.pcbi.1004478)
Supplement: S2 Material — (ZIP) [file pcbi.1004478.s002.zip › predictions_40_or_more/SMa0762.pdf]

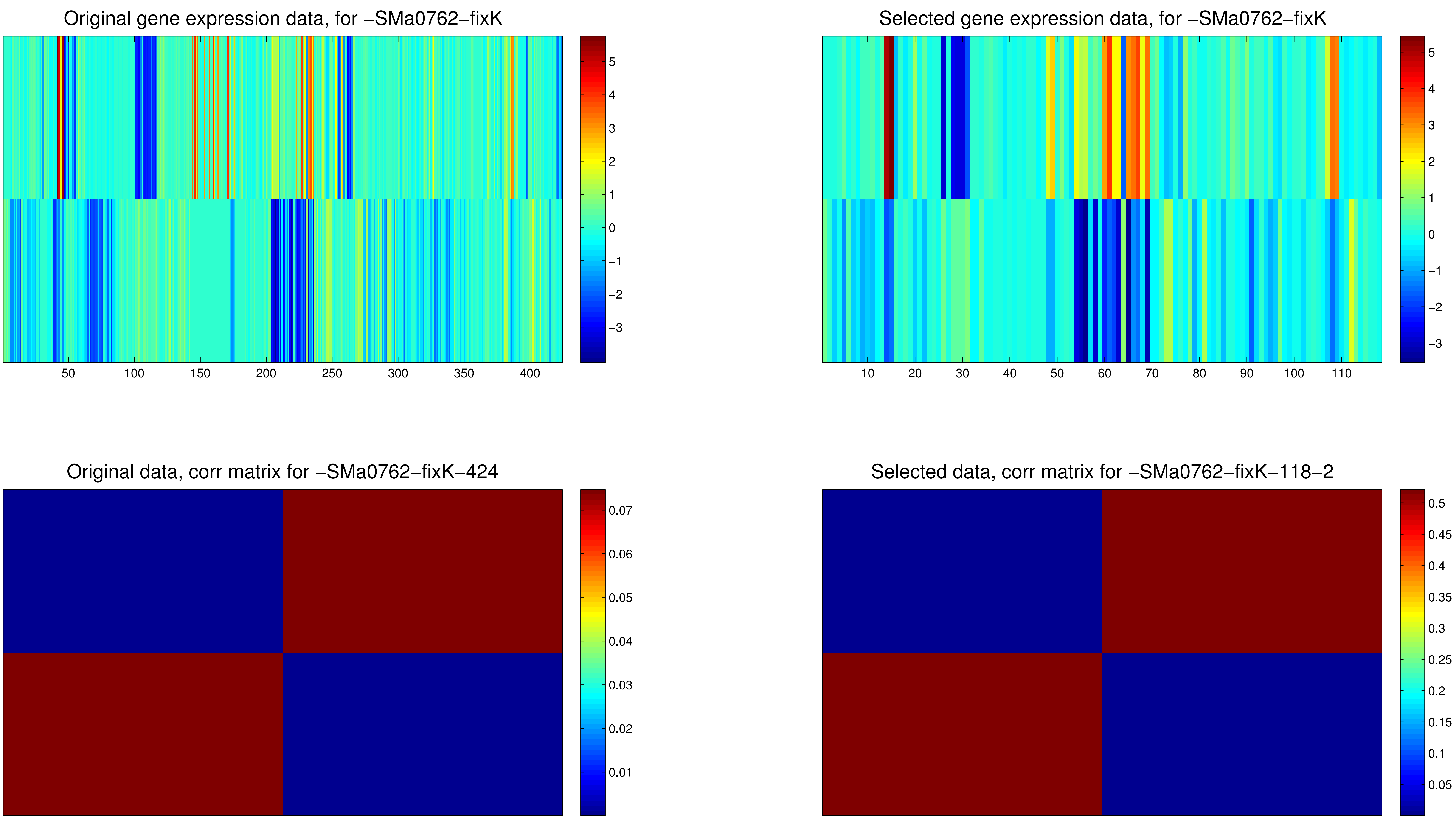

Original gene expression data, for −SMa0762−fixK
50
100
150
200
250
300
350
400
5
4
3
2
1
0
−1
−2
−3
Selected gene expression data, for −SMa0762−fixK
10
20
30
40
50
60
70
80
90
100
110
5
4
3
2
1
0
−1
−2
−3
Original data, corr matrix for −SMa0762−fixK−424
0.07
0.06
0.05
0.04
0.03
0.02
0.01
Selected data, corr matrix for −SMa0762−fixK−118−2
0.5
0.45
0.4
0.35
0.3
0.25
0.2
0.15
0.1
0.05
