# Supplementary figures and images for: Evolution of Intra-specific Regulatory Networks in a Multipartite Bacterial Genome
Source: PLoS Comput Biol. 2015 Sep 4;11(9):e1004478. doi: 10.1371/journal.pcbi.1004478 (PMC4560400; doi:10.1371/journal.pcbi.1004478)

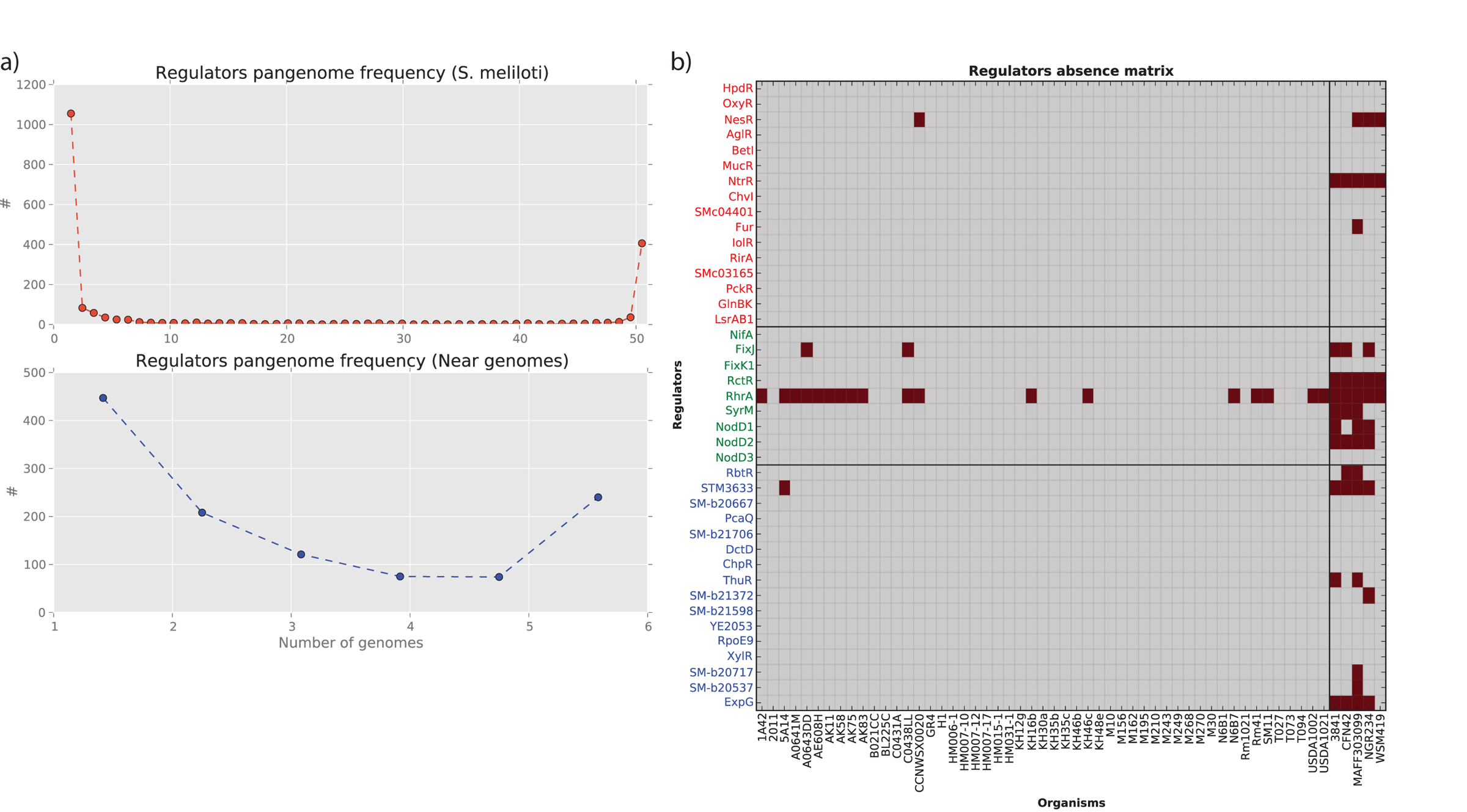

Supplement: S1 Fig — a) TFs frequency (expressed as the number of strains having the TF encoded in their genome) in S. meliloti and the other rhizobial genomes; b) TF presence/absence matrix in the strains analysed in this study: red indicates the TF absence. TFs are colored according to the replicon they belong to: red for chromosome, green for the pSymA megaplasmid and blue for the pSymB chromid. (TIF) [file pcbi.1004478.s008.tif]

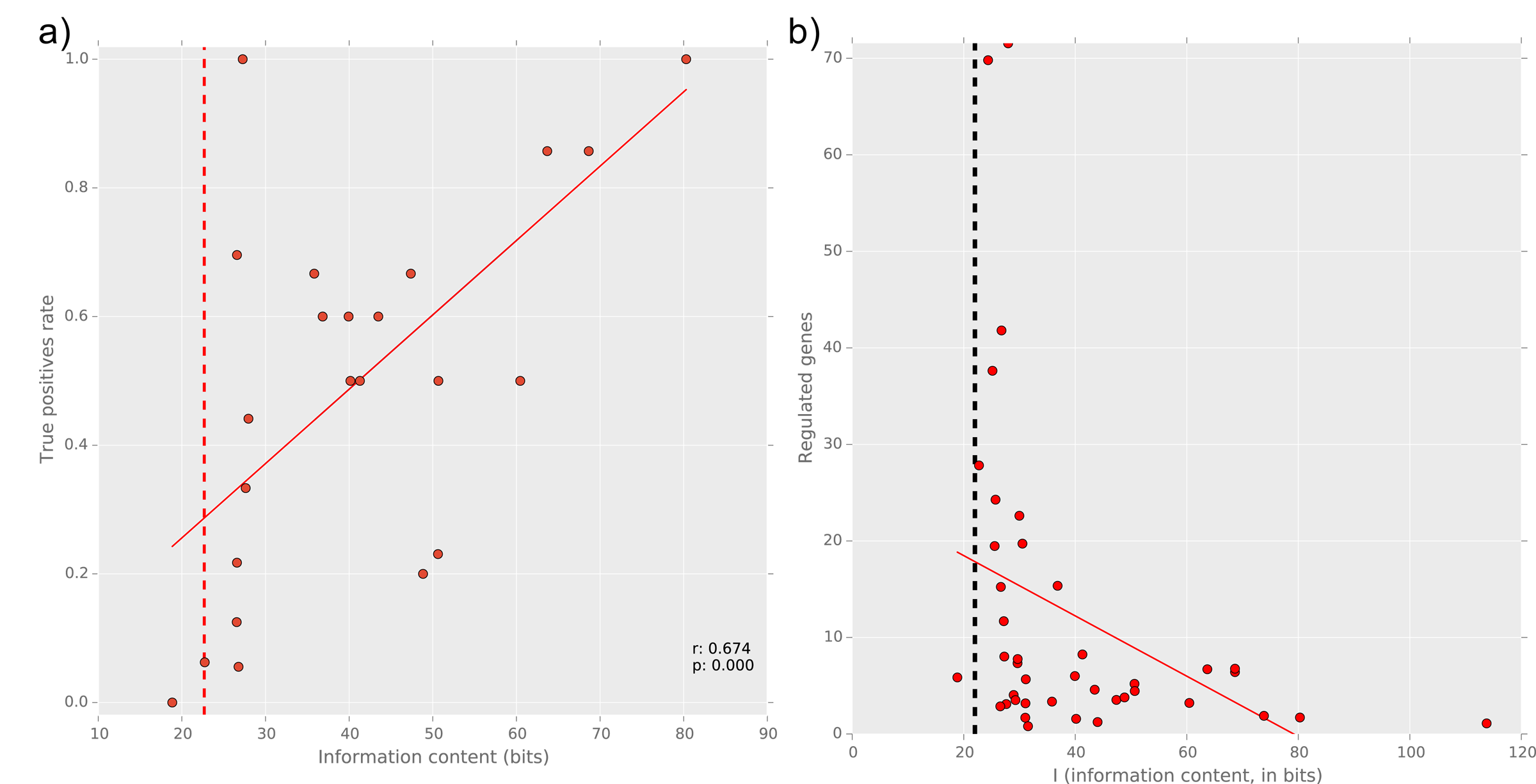

Supplement: S2 Fig — Vertical dashed line indicates the minimum information content for S. meliloti strain Rm1021. a) Correlation between predictions true positive rate and information content; b) Correlation between the number of predicted regulated genes and information content. (TIF) [file pcbi.1004478.s009.tif]

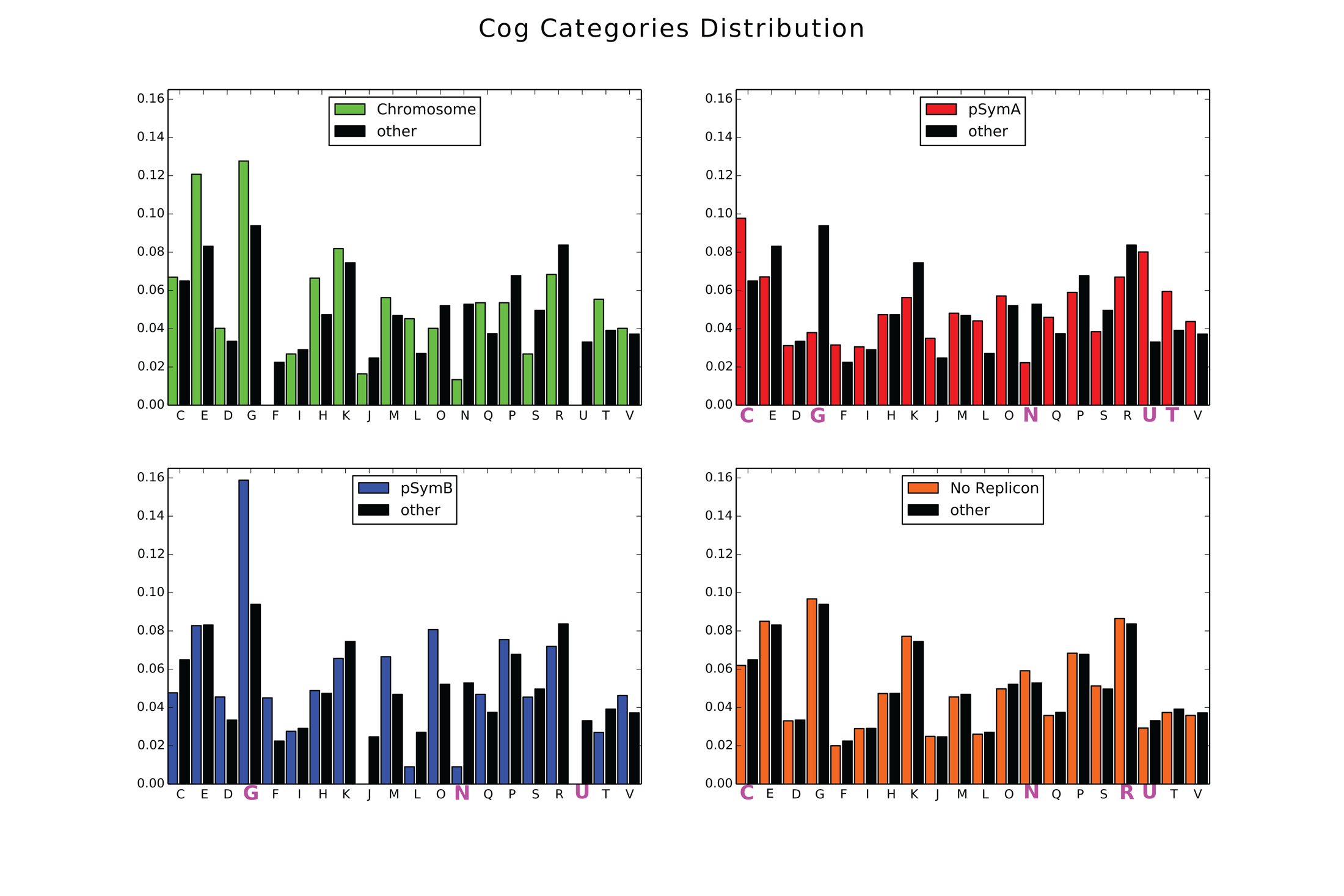

Supplement: S3 Fig — For each replicon, the proportion of regulated downstream genes belonging to each category is compared with the genes belonging to other replicons. Purple categories indicate a statistically significant enrichment. (TIF) [file pcbi.1004478.s010.tif]

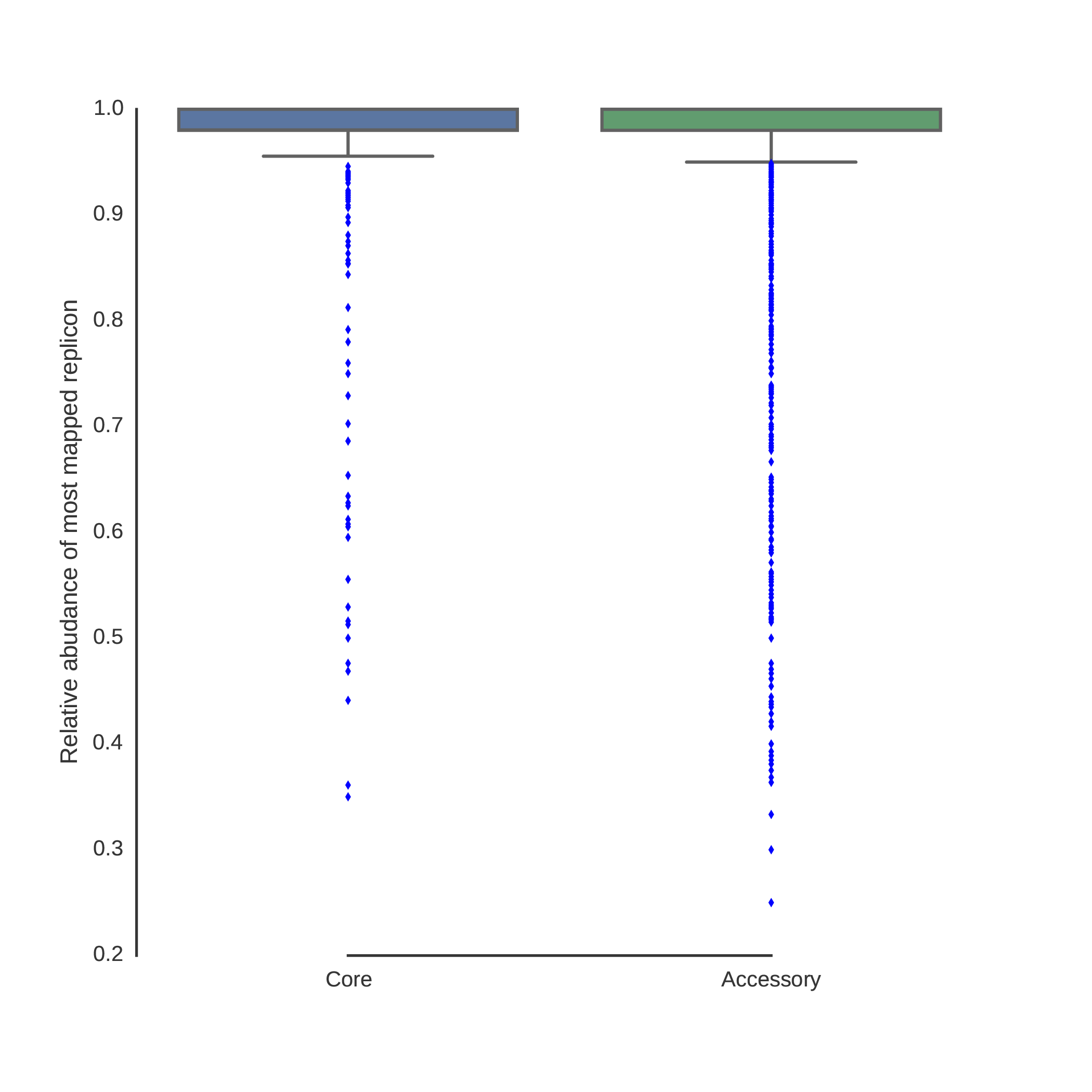

Supplement: S4 Fig — For each orthologous group in the S. meliloti pangenome, the abundance of the most mapped replicons has been computed as a proxy for the consistency of the replicon mappings. (TIF) [file pcbi.1004478.s011.tif]
